# An actin remodeling role for *Arabidopsis* processing bodies revealed by their proximity interactome

**DOI:** 10.15252/embj.2022111885

**Published:** 2023-02-06

**Authors:** Chen Liu, Andriani Mentzelopoulou, Amna Muhammad, Andriy Volkov, Dolf Weijers, Emilio Gutierrez‐Beltran, Panagiotis N Moschou

**Affiliations:** ^1^ Department of Plant Biology, Uppsala BioCenter Swedish University of Agricultural Sciences and Linnean Center for Plant Biology Uppsala Sweden; ^2^ Department of Biology University of Crete Heraklion Greece; ^3^ Institute of Molecular Biology and Biotechnology, Foundation for Research and Technology‐Hellas Heraklion Greece; ^4^ University Institute of Biochemistry and Biotechnology, PMAS‐Arid Agriculture University Rawalpindi Rawalpindi Pakistan; ^5^ Laboratory of Biochemistry Wageningen University and Research Wageningen The Netherlands; ^6^ Instituto de Bioquímica Vegetal y Fotosíntesis, Universidad de Sevilla and Consejo Superior de Investigaciones Científicas Seville Spain; ^7^ Departamento de Bioquímica Vegetal y Biología Molecular, Facultad de Biología Universidad de Sevilla Sevilla Spain

**Keywords:** ARP2–ARP3, condensates, LLPS, plasma membrane domains, SCAR–WAVE, Cell Adhesion, Polarity & Cytoskeleton, Plant Biology, RNA Biology

## Abstract

Cellular condensates can comprise membrane‐less ribonucleoprotein assemblies with liquid‐like properties. These cellular condensates influence various biological outcomes, but their liquidity hampers their isolation and characterization. Here, we investigated the composition of the condensates known as processing bodies (PBs) in the model plant *Arabidopsis thaliana* through a proximity‐biotinylation proteomics approach. Using *in situ* protein–protein interaction approaches, genetics and high‐resolution dynamic imaging, we show that processing bodies comprise networks that interface with membranes. Surprisingly, the conserved component of PBs, DECAPPING PROTEIN 1 (DCP1), can localize to unique plasma membrane subdomains including cell edges and vertices. We characterized these plasma membrane interfaces and discovered a developmental module that can control cell shape. This module is regulated by DCP1, independently from its role in decapping, and the actin‐nucleating SCAR–WAVE complex, whereby the DCP1–SCAR–WAVE interaction confines and enhances actin nucleation. This study reveals an unexpected function for a conserved condensate at unique membrane interfaces.

## Graphical abstract

Plant processing body component DCP1 interacts with the actin‐nucleating complex SCAR/WAVE at the plasma membrane to regulate cell shape and tissue growth.

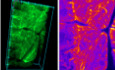

## Introduction

Proteins may participate in multivalent interactions with themselves or one another. Multivalency can depend on weak interactions between charged residues, dipoles, and/or aromatic groups often displayed by so‐called “intrinsically disordered regions” (IDRs). These weak interactions can promote their dissociation from the bulk protein pool to form droplet‐like assemblies through, for example, liquid–liquid phase separation (LLPS; Beutel *et al*, 2019). LLPS is a state favoring condensation through weak intra‐ or intermolecular interactions. We use the term “condensates” hereafter to describe such proteinaceous assemblies. LLPS occurs when the bulk concentration exceeds a threshold above which molecules spontaneously partition into condensates that can resemble droplets due to surface tension. Transient or more stable condensates form in the nucleus, cytoplasm, or plasma membrane (PM) interfaces, for example, the animal‐specific junction adherent molecules (Beutel *et al*, 2019; Zaccara & Jaffrey, 2020).

Condensates can coarsen, increase or decrease in size over time on membrane surfaces (Snead *et al*, 2022). Likewise, the archetypal and evolutionarily conserved condensates known as processing bodies (PBs), which are rich in proteins and RNA and modulate RNA silencing, decapping and decay, undergo fission at membrane surfaces of the endoplasmic reticulum (ER) through an unknown mechanism (Lee *et al*, 2020). In accordance with their functions, PBs contain decapping and exosome complexes, deadenylases, RNAs, and RNA‐binding proteins. In PBs, including in plants, proteins such as the RNA decapping complex comprising mainly DECAPPING 1 (DCP1) and DCP2, and the proteins DCP5, VARICOSE (VCS), Protein Associated with Topoisomerase II 1 (PAT1), as well as the EXORIBONUCLEASE 4 (XRN4) localize there (Xu *et al*, 2006; Xu & Chua, 2009; Rymarquis *et al*, 2011; Roux *et al*, 2015). Although systematic studies of plant PBs are lacking, animal PBs appear to be long‐term storage sites for mRNAs poised to be released for translation following specific cues related to stress, metabolism, and translation capacity.

Albeit readily visible in cells, as aforementioned, condensates such as PBs depend on a meshwork of weak interactions and lack a surrounding membrane, which makes their isolation challenging. Proximity‐dependent biotin ligation (or PDL) harnesses covalent biotinylation of interacting proteins with or near neighbors of a particular prey protein; we and others have recently established PDL approaches in various plants (reviewed in Zhang *et al*, 2022). By bypassing the need to retain native interactions for their identification, PDL holds promise to delineate the organizational and functional principles of condensates.

Inspired by PDL applications for the elucidation of condensates in non‐plant models (Youn *et al*, 2018), we established a PDL pipeline to determine the protein composition of condensates in plants, using PBs as a proof of concept. We show here that PBs are organized into both known and previously unknown functional modules. We further discovered that PBs components interface with PM domains, accumulating at cell edges or vertices. The suppressor of the cAMP receptor (SCAR)–WASP family verprolin homologous (WAVE) complex modulates this interface by recruiting DCP1. The SCAR–WAVE–DCP1 link enhances actin nucleation likely through the actin‐related protein complex 2/3 (ARP2–ARP3; Kim *et al*, 2021; Lee & Szymanski, 2021; Qin *et al*, 2021). This SCAR–WAVE–DCP1 nexus functions as a coordinated cellular system that culminates in growth regulation.

## Results

### A bait for PB proteome capture

**Figure 1:**
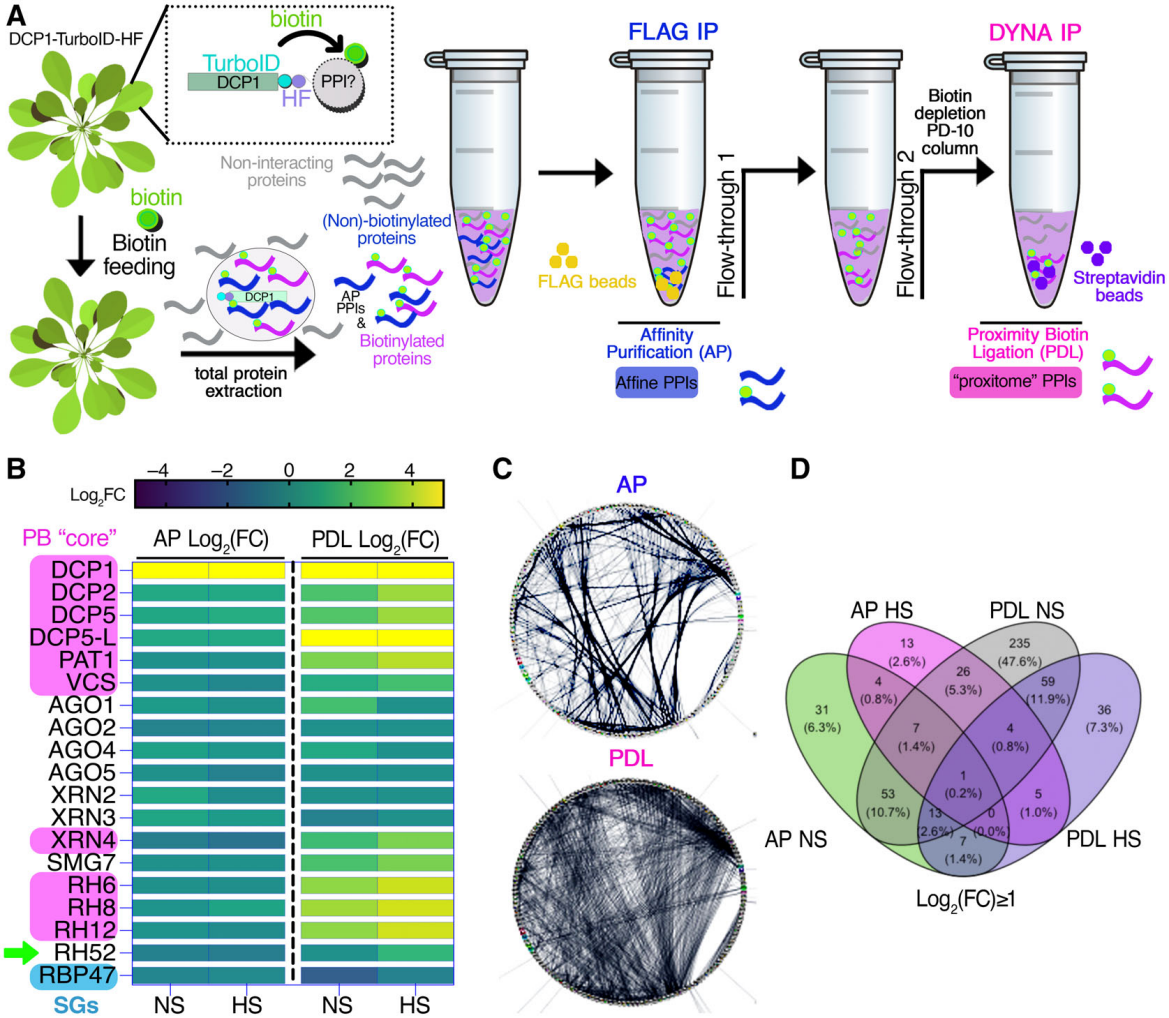
The pipeline of the APEAL approach Overview of the APEAL pipeline. Upon 24 h biotin feeding and treatment (supplied with 50 μM biotin directly into leaves of 4‐week‐old plants by syringe infiltration), total proteins are extracted from infiltrated leaves. The proteome is subjected to AP‐immunocapture of the FLAG tag. In the AP step, some of the captured proteins will be biotinylated. The PDL step uses the leftover supernatant from the AP step and captures biotinylated proteins with streptavidin beads. PPI, protein–protein interactions; AP, affinity purification; PDL, proximity‐dependent biotin ligation; FLAG IP, FLAG‐beads immunoprecipitation; DYNA‐IP, streptavidin‐bead immunoprecipitation. We use the term “proxitome,” to describe the proteins captured by the PDL step of APEAL. These proteins may not physically interact with DCP1.Heatmap showing the “core processing body (PB)” (magenta) components identified and other linked proteins. RBP47 is a stress granule marker (SGs; blue). RH52 is a new helicase identified as a PB component (green arrow). The scale on the right shows log_2_FC of protein abundance. Note that only the PB core components were enriched in the PDL (i.e., the proxitome, log_2_FC ~ 1 or above) but not in AP. Furthermore, heat stress (HS) increased the enrichment of some PB core components.Comparison of AP/PDL interacting networks produced from APEAL. STRING density plots of pairwise interactions between proteins obtained from the AP or PDL steps (combined interactions found in non‐stress [NS]/[HS]). Note that PDL produces an overall denser interaction network (under standard parameters, the same number of proteins was selected for AP/PDL).Venn diagram showing the proteins identified for PDL and AP in NS and HS samples (PPIs fulfilling the criterion log_2_FC > 1). Source data are available online for this figure.

**Figure EV1:**
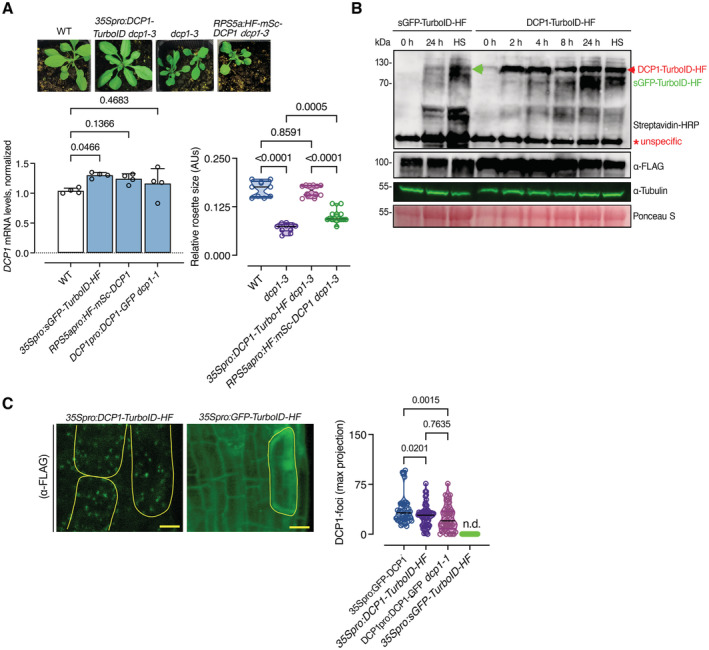
Establishment of a functional DCP1 bait for PDL The *DCP1‐TurboID‐HF* construct efficiently rescues the adult *dcp1‐3* mutant phenotype. Phenotypes (3‐week‐old) of adult plants expressing *35Spro:DCP1‐TurboID‐HF* and *RPS5apro:HF‐mScarlet‐DCP1* in the *dcp1‐3* mutant background. In a semi‐controlled greenhouse setting (temperature control 22°C), the *dcp1‐3* mutant showed a smaller adult stature. Lower: complementation quantification (rosette diameter), *N*, *biological replicates =* 4, *n* = (*pooled data of 3 biological replicates*) 5–7 rosettes, error bars are (mean + SD) and RT‐qPCR analyses for the quantification of *DCP1* expression levels. *PP2A* and *Actin7* were used for normalization (1‐week‐old) seedlings (*N*, *biological replicates =* 2, *n* (*pooled data of 3 biological replicates*) = 3, error bars are mean + SD). When DCP1 was driven by the RPS5a promoter (stem cell‐specific promoter), we observed partial complementation, suggesting that DCP1 is important also in non‐meristematic cells.Immunoblot analyses of lines expressing sGFP‐TurboID‐HF or DCP1‐TurboID*‐HF* upon NS or HS conditions (same as used for APEAL). The immunoblots also show the accumulation of auto‐biotinylated sGFP‐TurboID‐HF or DCP1‐TurboID‐HF in a time course of biotin administration (as detected with streptavidin‐HRP, which captures biotinylated proteins). 50 μM Biotin was delivered in leaves by syringe infiltration and diffusion. Note that HS did not increase the biotinylation efficiency of DCP1‐TurboID‐HF: at 24 h, compare samples “2” with “4” in the “DynaIP”. HF, 6xhis‐3xFLAG. The red arrowhead indicates the position of DCP1‐TurboID‐HF and green for sGFP‐TurboID‐HF (*N*, *biological replicates* = 2). We used the same scheme for biotin application as determined in (Arora *et al*, 2020). The 2 h NS/HS corresponds to the timing after the administration of biotin for 24 h (*t* = 0 corresponds to 24 h biotin administration in NS conditions). The red asterisk indicates non‐specific band. α‐FLAG was used for the detection of DCP1‐TurboID‐HF and sGFP‐TurboID‐HF (similar size ~ 130 kDa). α‐Tubulin and ponceau staining were used for loading control.Representative confocal micrographs showing that the localization of DCP1 (α‐FLAG, 5‐day‐old seedlings, root cap cells) to PBs is retained in lines expressing *DCP1‐TurboID‐HF*. As a control, the *GFP‐TurboID‐HF* line was used that does not show localization to PBs. Right: number of DCP1‐positive foci in the corresponding lines expressing *DCP1* (*N*, *biological replicates =* 1, *n* = 32–60 cells). Data information: In (A and B), *P* values were determined by ordinary one‐way ANOVA. Upper and lower lines in the violin plots when visible, represent the first and third quantiles, respectively, horizontal lines mark the median and whiskers mark the highest and lowest values. Source data are available online for this figure.

To establish an approach for determining the protein composition of condensates in plants, we focused on PBs as a proof of concept, as non‐plant PBs are omnipresent. A stepwise PDL‐based approach might be the most appropriate for determining the composition of condensates, as we assumed that incorporating a standard affinity purification (AP) step before a subsequent PDL step might help distinguish between strong or direct versus the more specific interactions for condensates which are transient and weak. We thus used DCP1 as a bait tagged with both the sequence encoding for FLAG peptide and the biotin ligase “TurboID” as a construct encoding a chimeric DCP1‐TurboID‐6x **H** is‐3x **F** LAG (driven by the *35S* promoter; *35Spro:DCP1‐TurboID‐HF*). The FLAG can be used for AP of DCP1 through α‐FLAG beads and the TurboID for PDL. We describe the pipeline in Fig 1A and the technical details in the Materials and methods. As a specificity control for our assays, we used lines expressing GFP fused to 3xFLAG and TurboID (*35Spro:GFP‐TurboID‐HF*); the TurboID was described in (Arora *et al*, 2020). DCP1‐TurboID‐HF was functional, showing higher expression levels (~ 25%, *P* = 0.047, ANOVA) than were seen in wild‐type (WT) seedlings, it could rescue the decreased size of adult plants of the *dcp1‐3* mutant (Martinez de Alba *et al*, 2015), and could lead to efficient proteome biotinylation (Fig EV1A–C). Therefore, this DCP1 fusion is a physiologically relevant bait with which to explore the PB interactome.

### APEAL captured the PB proteome

**Figure EV2:**
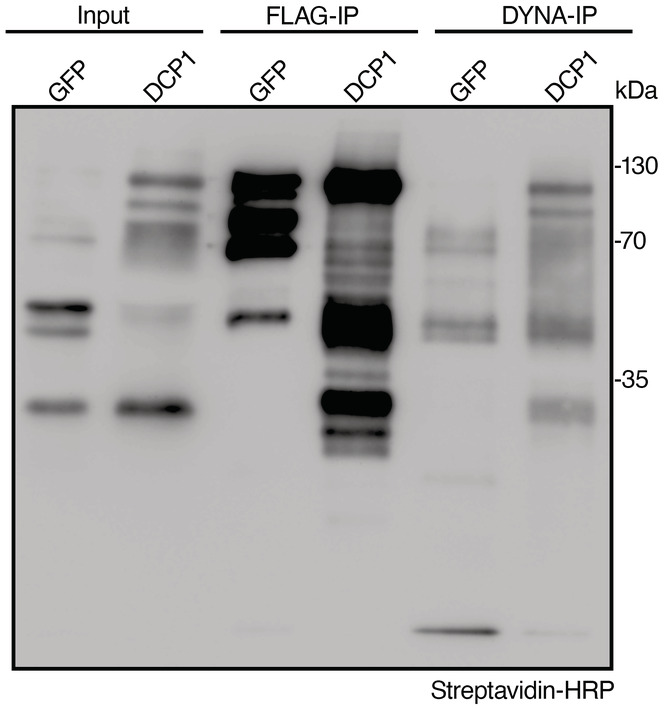
Biotinylated proteins evade purification from the AP step in APEAL Immunoblot analyses from lines expressing *GFP‐TurboID‐HF* or *DCP1‐TurboID‐HF* (denoted as GFP or DCP1, respectively) showing the presence of biotinylated proteins in the flow‐through after the AP‐step (for flow‐through 1, see DYNA‐IP here). The blot was overexposed to detect the faint streptavidin smear in flow‐through 1. The baits in these experiments undergo auto‐biotinylation, as reported previously. The results are representative of one experiment performed three times. Note that the immunodetectable biotinylation levels did not correlate well with the hits identified in Fig EV5 (compare GFP to DCP1). GFP and DCP1 are of similar molecular weight, while the bands below the upper band (~ 130 kDa) likely correspond to proteolytic cleavage products.

We asked whether the AP/PDL combination using the DCP1 bait could indeed capture both direct interactions but also weak ones. We define the PDL results hereafter as the PBs “proxitome.” After the AP step, we detected biotinylated proteins in the remaining supernatant (Figs 1A and EV2, corresponding to the “flow‐through 1”). We thus decided to further consider proteins obtained from both the AP and PDL steps; this tandem analytical approach is referred to hereafter as “APEAL” (for tandemly coupled **A** ffinity **P** urification with Proximity‐d **E** pendent **L** ig **A** tion steps). We coupled APEAL with nano‐liquid chromatography–tandem mass spectrometry (nano‐LC–MS/MS) in biological duplicates on DCP1 baits using 4‐week‐old rosettes grown under heat stress (HS) conditions (37°C for 2 h) and non‐stress (NS) conditions. We selected HS treatment to benchmark APEAL because HS induces a size and number increase in PBs and likely a change in their composition (Gutierrez‐Beltran *et al*, 2015), and it enhances RNA decapping through the increased association of DCP1–DCP2 with polysomes (Merret *et al*, 2013). We observed that HS does not significantly affect biotinylation levels of DCP1 (Fig EV1B, i.e., “24 h” vs. “HS”, the two samples obtained under the same setting: 24 h application of biotin with or without HS), suggesting that any differences in the PB proteome between NS and HS conditions in the APEAL may be of biological relevance.

**Figure EV3:**
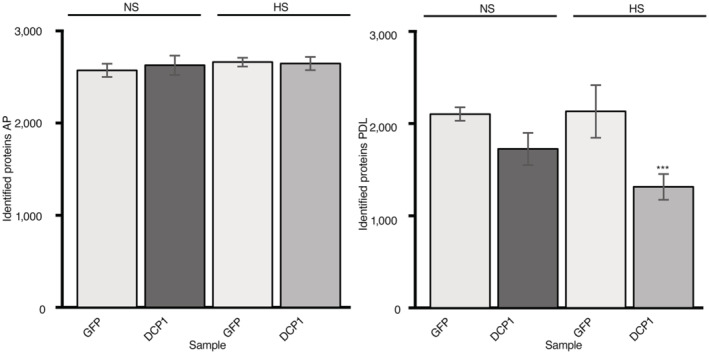
Hits from the APEAL approach (AP and PDL steps) Total protein hits from mass spectrometry under NS or HS conditions following the AP (left) or PDL (right) steps of APEAL. We used the same scheme for biotin application, as described (Arora *et al*, 2020). The results presented are unfiltered, containing the noisy portion of the proteome. Note that the free diffusion of GFP *in vivo* led to increased proteins identified. *sGFP‐TurboID‐HF*, GFP; *DCP1‐TurboID‐HF*, DCP1 (*N*, *biological replicates =* 3, error bars are mean ± SD). Note the increased numbers of hits in GFP/PDL reflect the noisy proteome. GFP is expected to produce more noise (translated as hits in the context of the proteome), due to the increased diffusion over the specifically and topologically restricted DCP1 (confirmed in Fig EV1, localizations). The decreased number of hits for HS conditions corresponds mainly to proteins of signal transduction and metabolism, as well as vesicle trafficking proteins (see also Fig 2 and below for an explanation: HS reduces the DCP1 association with the PM). Furthermore, DCP1, as described below, loses localization at the PM during HS. Data information: AP/PDL GFP samples did not differ at *P* < 0.005 (determined by an unpaired *t*‐test); ***P ≤ 0.05, as determined by an unpaired *t*‐test for comparison between HS and NS GFP samples.

Proteome analyses yielded almost equivalent numbers of protein hits for AP and PDL steps (Fig EV3). Unexpectedly, and despite the size increase in PBs noted above, HS led to fewer proteins being captured in PDL/HS from DCP1, suggesting that upon HS the DCP1 molecules may be spatially further confined interacting will fewer proteins (see below for a possible explanation). To test for enrichments across the different samples, we applied the same criteria described in our previous work to define the high‐confidence proximity interactions (Arora *et al*, 2020). Briefly, we considered only interactions present in both biological replicates (log_2_[fold change] ≥1, *n* = 2, false‐discovery rate [FDR] = 0.05; Source Files 1 and 2, for unfiltered and filtered data, respectively). To compare the AP and PDL datasets, we also assigned LFQ (label‐free quantitation) and iBAQ values (intensity‐based absolute quantification, Source Files 3 and 4; Cox *et al*, 2011) to the semi‐quantitative peptide enrichments and reiterated interactions. The LFQ/iBAQ assignments produced similar results to the semiquantitative analysis.

An inspection of protein hits in the PDL step confirmed an enrichment for conserved PB core components (Fig 1B; log_2_FC ˃ 1; Gutierrez‐Beltran *et al*, 2021). As a cautionary note, we do not define the PB core as a structural entity with topological essence (e.g., with the PB center formed as an immiscible liquid), but rather as an indispensable set of critical or accessory proteins that comprises part of the decapping machinery. Intriguingly, the AP step failed to enrich for PB core components, suggesting that many weak interactions are not retained, thus validating our dual approach (Fig 1B; “AP” vs. “PDL”). Importantly, APEAL showed no enrichment for proteins exclusively associated with the other condensate known as stress granules (SGs; e.g., RNA‐BINDING PROTEIN 47B [RBP47]), despite the putative physical links between the two condensates during HS (Gutierrez‐Beltran *et al*, 2021). One exception was the class of RNA helicases which are present in both SGs and PBs (Fig 1B; log_2_FC ≥ 1.5, RH6, RH8, RH12, and the newly discovered component of PBs RH52 herein). The absence of other SG components confirmed that APEAL, at least, in this case, can provide the required spatial resolution to specifically resolve the composition of PBs and highlights the functional differences between the PBs and SGs.

### Network analyses reveal that PBs interface with membranes

**Figure 2:**
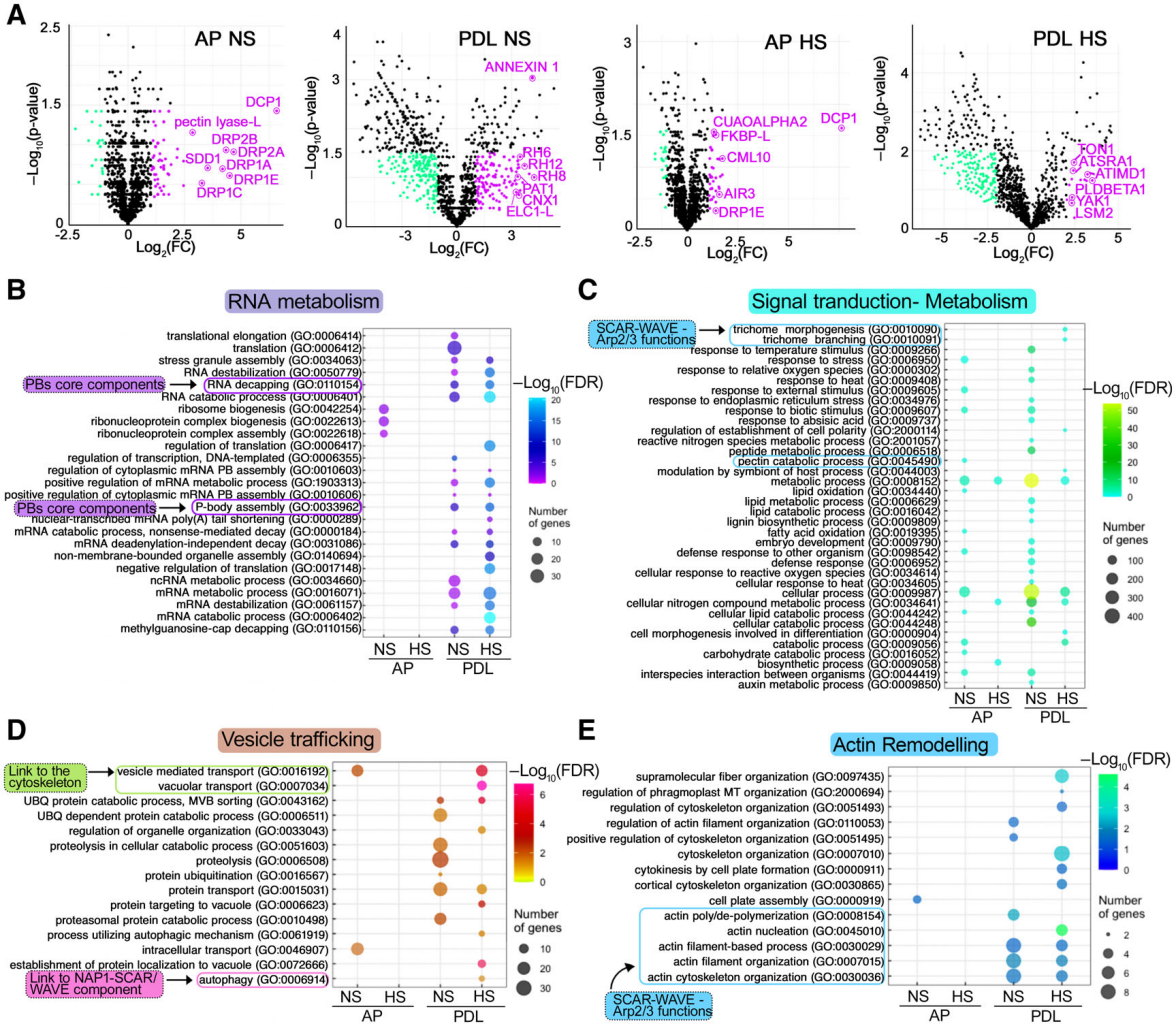
APEAL captured the PB proteome and proxitome AVolcano plots showing significantly enriched proteins in NS and HS conditions from the AP and PDL APEAL steps. Selected proteins are indicated in magenta and are encoded by genes that belong to the identified subnetworks described in (C) and (D). Magenta indicates enrichment in HS samples; cyan indicates depletion in HS samples.B–EGene Ontology (GO) enrichment analyses of the APEAL results, divided into four subnetworks. Note the terms related to vesicle trafficking and actin remodeling. A more detailed description is provided in Fig EV5 and in the Appendix text. Note that signal transduction proteins, metabolism‐related proteins, and vesicle trafficking proteins evade PBs during HS (Fig EV5, reduced hit number), while the opposite pattern is observed for actin and RNA metabolism subnetworks. FDR, false discovery rate. Important links described below are indicated (i.e., to the SCAR–WAVE/ARP2–ARP3 (and the component NAP1 which is part of the SCAR–WAVE and links to autophagy), cytoskeleton, PB core, and cell‐wall‐related metabolism). Source data are available online for this figure.

**Figure EV4:**
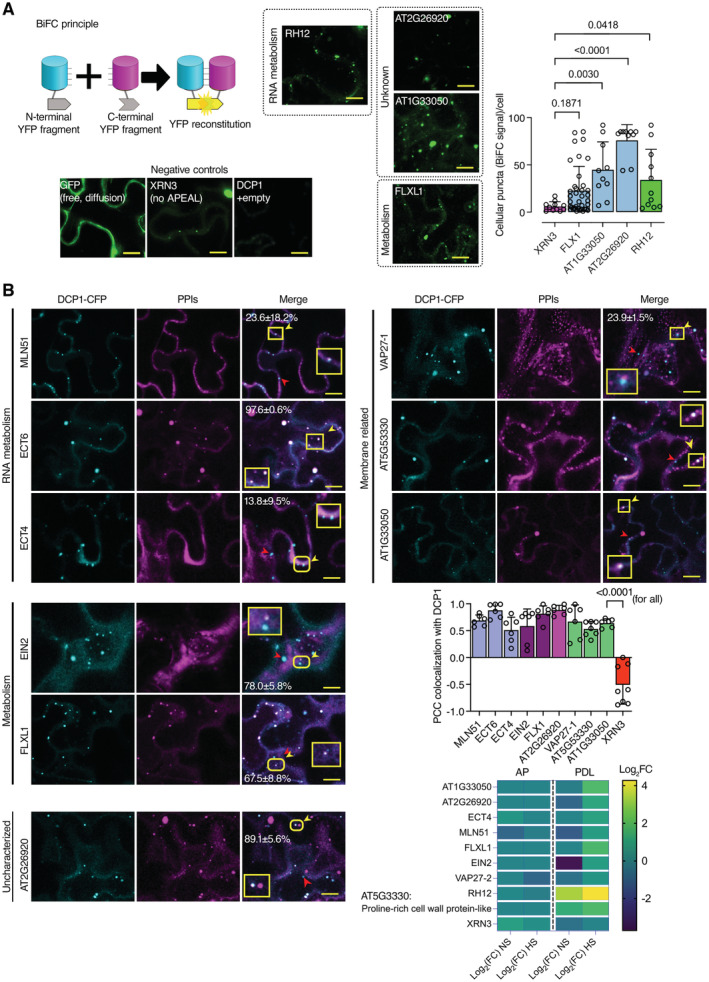
DCP1 colocalization and association with novel and known interactors in transient expression of N. benthamiana leaves Bimolecular fluorescence complementation (BiFC) assay of the indicated proteins. Left: the cartoon depicts the BiFC concept and YFP reconstitution caused by protein–protein interaction *in vivo*. When two proteins interact, the cYFP and nYFP halves are brought in proximity and produce a fluorescent signal. For protein selection, we classified the associations with DCP1, in both AP and PDL steps, according to their relative enrichment (log_2_FC). We selected proteins presenting moderate relative enrichment in the AP step, while having high predictability in the PDL step (log_2_FC > 0 in both steps). As an additional filter, we selected proteins rich in IDRs (half of the proteins from the list have high prion‐like domain (PRD) scores and high Finum [aa]/total [aa] ratios as defined through the PLAAC algorithm; Source File 8), and as such proteins would be likely to localize to condensates. We tested the association between PBs (using DCP1 as one representative component) and selected five proteins from these bins using BiFC and colocalization assays (Source File 8). These results suggested that the APEAL approach may help identify PB components or DCP1 interactors. These interactions should be further studied in *Arabidopsis*. BiFC efficiency was estimated from the reconstituted YFP raw signal intensity and YFP‐positive puncta per cell in maximum projections (*N*, *biological replicates =* 3, *n* = 4–12 cells). Lower left: representative confocal micrographs showing that YFP signal is reconstituted at cellular puncta that most likely correspond to PBs. XRN3 represents negative control (see also B), as it localizes in the nucleus. Right: number of cellular puncta per total cell volume (in maximum projection images; *N*, *biological replicates =* 3, *n* (*pooled data of 3 biological replicates*) = 20 cells, error bars are mean + SD). Scale bars: 10 μm.Colocalization of selected proteins with DCP1‐CFP‐positive puncta (*35Spro:DCP1‐CFP* transgene). The coding sequences of the corresponding “interactors” (PPIs; direct or indirect, defined in APEAL) were driven by the *35Spro* and cloned in frame with *mCherry* at their 5′ end. Two‐color colocalizations were estimated by Pearson's correlation coefficients (PCC) using ultra‐fast super‐resolution microscopy combined with image deconvolution (~ 120 nm axial resolution). Numbers in “merge” indicate colocalization frequency between DCP1‐CFP and the corresponding interacting protein (*N*, *biological replicates =* 2, *n* = 5 cells). Yellow arrowheads indicate colocalization and red arrowheads lack of colocalization. Lower right: PCC of pixel intensities between DCP1‐CFP and the corresponding putative interacting protein (*N*, *biological replicates =* 3, *n* = 4–12 cells, error bars are mean + SD). We confirmed the ECT domain‐containing proteins, MLN51, FLXL1, EIN2, VAP27‐1, uncharacterized AT1G33050 (hypothetical protein), and AT5G53330/AT2G26020 (ubiquitin‐associated/translation elongation factors EF1B) as novel PB components. By applying a pre‐selection criterion of enrichment (log_2_FC > 0.5) for the selection of prey, we significantly increased the probability of identifying successful binary interactions between PBs (i.e., cYFP‐DCP1) and the identified proteins. All preys were confirmed as PB components, while PDL had higher interaction predictive power than the AP step irrespective of whether proteins were enriched in NS or HS. XRN3 was used as a threshold control (log_2_FC = −0.58, PDL/NS conditions). The heatmap shows the enrichment of these proteins in the different conditions; the scale at right shows log_2_FC. Data information: In (A and B), *P* values were determined by ordinary one‐way ANOVA (differences were all calculated compared to XRN3). Source data are available online for this figure.

We integrated our PDL hits with delimited direct protein–protein interaction (PPI) data from the STRING (Search Tool for the Retrieval of Interacting Genes/Proteins) database (Jensen *et al*, 2009), revealing that the PDL dataset forms a denser PPI network than that obtained with AP. The average numbers of interactions from PDL per protein were 5.4 (*P* = 5.6e^−11^) and 4.1 (*P* = 2.1e^−10^) for NS and HS, respectively (Fig 1C). The PDL dataset also showed a different profile in terms of interactions from AP, while different interactions were observed when HS and NS were compared (Figs 1D and 2A). Consistent with the expected incremental formation of PBs during HS, the term “PΒs” was more enriched in the PDL dataset upon HS when compared to NS (*P* = 6.9e^−2^ vs. 6.9e^−22^). We validated the results of the PDL step using bi‐fluorescence complementation (BiFCs; concept described in Fig EV4A, negative control XRN3, *P* = 0.19 to < 5.7e^−4^) and colocalization analyses in the heterologous *Nicotiana benthamiana* transient expression system, showing the successful identification of novel components of PBs or DCP1 interacting proteins (Fig EV4A and B; nine out of nine proteins tested). We detected a partial overlap between our datasets and other published proteomics datasets that analyzed PM composition by AP with various baits (Source Files 5 and 6; overlap 27.1%; Fernandez‐Calvino *et al*, 2011; Bernfur *et al*, 2013; Rutter & Innes, 2017), and with endomembranes (overlap 9–32.8%; Heard *et al*, 2015), suggesting that PBs may interface with membranes. In comparison with a recent study examining DCP1 and DCP2 interactions through AP, we found an overlap of up to 36.3% (Schiaffini *et al*, 2021). Notably, this comparison showed a lower‐than‐expected overlap with our datasets, while in this previous study similar membrane‐related proteins were missing. Although in this previous study, the stabilizer of weak interactions formaldehyde was used, because it has the shortest span (~ 2–3 Å) of any known crosslinker (Nadeau & Carlson, 2007), it may not be the best option to stabilize interactions at the interface between condensates and membrane proteins. More crosslinkers with various spans will need to be tested, but we speculate for now that proxitomes may expand identifications of new proteins in PPI studies. Furthermore, our datasets suggested that PB may interface with membranes; we clarify this in detail below.

**Figure EV5:**
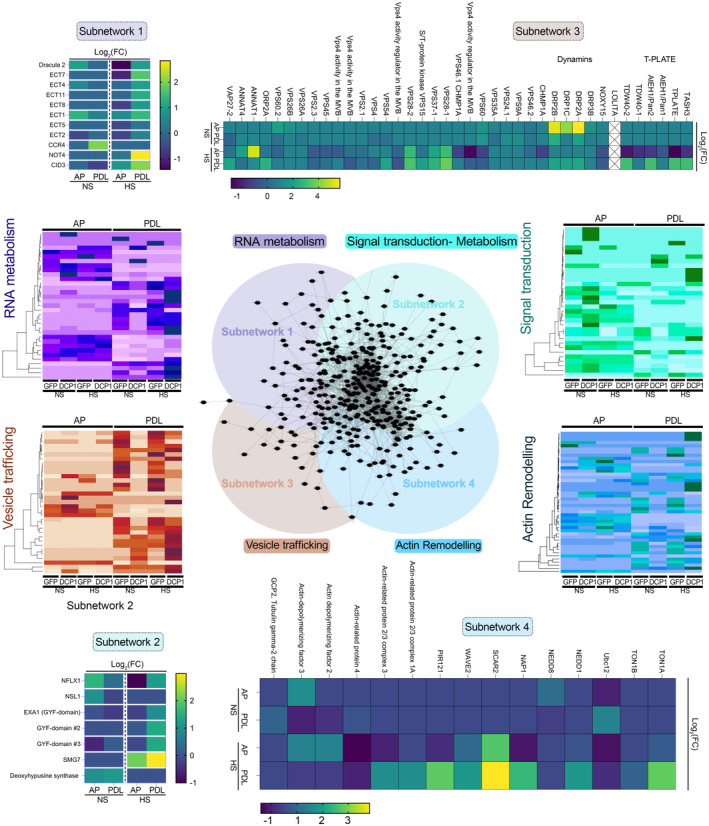
Structure and density of the four interconnected networks Subcluster analyses of APEAL reveal four interconnected subnetworks (center). The four networks were as follows: proteins related to RNA metabolism; signal attenuation, translation and metabolism; vesicle trafficking and actin remodeling. Heatmaps depict the abundance of selected proteins from the four subnetworks. Notably, the AP step in the “purple” heatmap did not lead to the identification of RNA metabolism proteins. In Appendix text, we summarize interesting hits from each network.Source data are available online for this figure.

We tested hierarchical linkages between the networks we generated by integrating the data from STRING with our datasets using hypergeometric tests (FDR = 0.05). We assigned four dense and interconnected subnetworks to PBs according to overrepresentations in AP and PDL datasets from both HS and NS conditions (Figs 2 and EV5; note that in volcano plots in PDL the DCP1 was not the most abundant). Subnetwork 1 is related to RNA metabolism (Fig 2B). In subnetwork 2, we obtained hits for phytohormone signaling, defense attenuation, translation, and metabolism (Fig 2C). Subnetwork 3 comprises an unexpected network for PBs, with hits linked to membrane remodeling and trafficking (Fig 2D); finally, subnetwork 4 includes another surprising network of proteins linked to the cytoskeleton and in particular actin remodeling (Fig 2E and Source File 7). In the Appendix text, we succinctly describe these networks, from which testable hypotheses may arise for future studies.

### DCP1 interfaces with plasma membrane domains at the cellular face independently of its role in decapping

**Figure 3:**
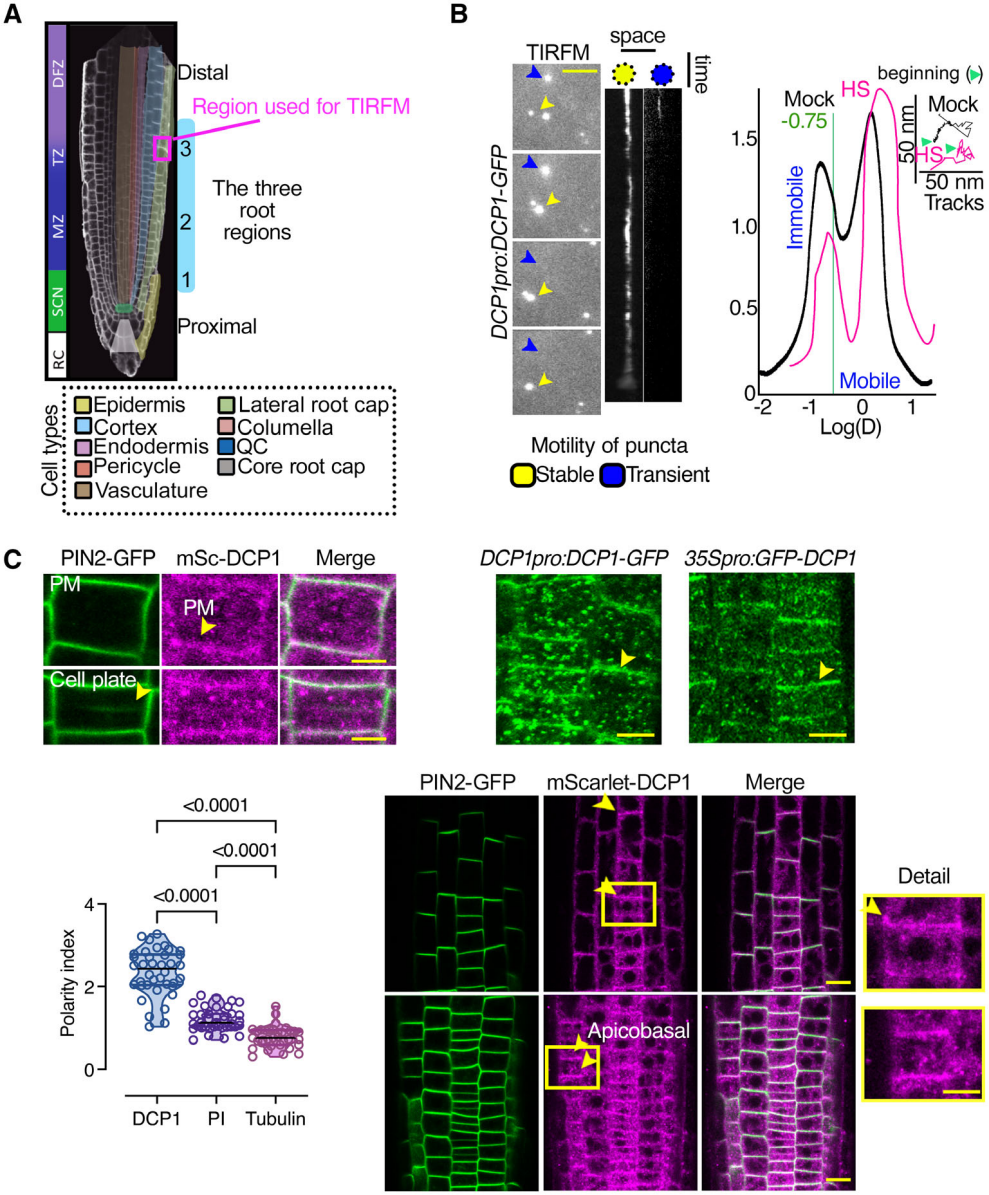
DCP1 protein interfaces with the plasma membrane Diagram of a root showing the three developmental regions (1–3) under examination in this study: stem cell niche (SCN; region 1), meristematic zone (MZ; region 2), and transition zone (TZ)‐differentiation zone (DFZ; region 3). The different cell types are color‐coded. The region used in TIRF‐M experiments is highlighted by the dashed magenta rectangle (region 3, see also B).Representative TIRF‐M of DCP1‐GFP (*DCP1pro:DCP1‐GFP* transgene) in the lateral PM of epidermal cells showing the transient attachment of DCP1‐positive puncta to the PM. Yellow arrowheads denote a PB that shows motility at the PM focal plane; blue arrowheads show a PB that transiently localizes to the PM. Scale bars: 2 μm. The corresponding kymographs are shown to the right. Right: distribution of immobile and mobile DCP1 molecules relative to the motility log(D) value of −0.75 (threshold; see Materials and Methods for details), in NS or HS conditions (D, diffusion coefficient). Inset: individual trajectories of mobile DCP1‐GFP in NS and HS conditions (500 frames, *n* = 120), showing a combination of directional and Brownian motion for both NS/HS. The green arrowheads denote the beginning of the NS and HS tracks for DCP1‐GFP.Representative confocal micrographs from lines co‐expressing *RPS5apro:HF‐mScarlet‐DCP1* and *PIN2pro:PIN2‐GFP* (epidermal cells, region 3, Scale bars: 5 μm). Bottom: polarity index of DCP1 in root meristematic cells (compared to propidium iodide (PI) and tubulin staining of root cells; *N*, *biological replicates =* 3 roots, *n* = 13 cells). Polarity index is calculated as the ratio of average of apical and basal VS lateral side of fluorescence signal intensity of the root epidemies cells. The arrowhead in PIN2 indicates the cell plate or PM in DCP1. Right: representative confocal micrographs showing that PM localization is independent of the promoter used (*DCP1pro*, *35Spro*; region 2, epidermal cells, or *RPS5apro* on the lower right). The details from the inset show increased localization at the cell edge (discussed later). mSc, mScarlet. Scale bars: 7 μm. Data information: In C, *P* values were determined by Wilcoxon. Upper and lower lines in the violin plots when visible, represent the first and third quantiles, respectively, horizontal lines mark the median and whiskers mark the highest and lowest values. Source data are available online for this figure.

The interfacing between condensates and membranes can be functionally important by regulating protein activities, as has been shown for animal cells (Snead *et al*, 2022). We thus asked whether the links between PBs and subnetwork 3, which suggested membrane functions, might relate to the decapping function of PBs. To observe membranes in detail and expedite analyses, we focused on the PM and used total internal reflection microscopy (TIRF‐M), which is suitable for viewing cell surface processes due to shallow illumination penetration (decay constant ~ 100 nm). TIRFM allowed us to observe occasional stalling and fusion of DCP1‐GFP‐positive droplets with one another at the lateral PM interface of meristematic root epidermal cells (Fig 3A and B). Though encouraging, we note here that these results should not be interpreted as a direct association between DCP1 and membrane lipids. As a cautionary note also, the lateral PM used for imaging due to the TIRF‐M inherent limitations which do not allow observation of apicobasal membranes, may not reflect the exact situation of these membranes. Furthermore, due to the swallow illumination depth, TIRFM does not allow observation of cells below the epidermis or the root cap in roots (e.g., cortex). Mean squared displacement (MSD) analyses, which track particles in two dimensions, showed that DCP1‐positive droplets become more stable with time, after an initial Brownian‐motion diffusion at the PM (Fig 3B, inset; track analyses). This tracking also showed that stable DCP1‐positive droplets lose signal with time (Fig 3B, kymograph). This result suggested that DCP1‐positive droplets may diffuse to form 2D domains at the inner face of the PM and highlights that subnetwork 3 may be of biological relevance.

High‐speed super‐resolution confocal microscopy (~ 120 nm axial resolution and 40 frames per second) and fluorescence partition analyses of the obtained micrographs between the PM and the overall signal at the cytoplasm (in PBs or the dilute phase) detected a fraction of mScarlet‐DCP1 at the PM (marked here with the auxin efflux carrier PINFORMED2 [PIN2]), regardless of the promoter driving the DCP1 (Fig 3C, the constitutive in meristems *RPS5a*, the strong *35Spro* or the native *DCP1pro*; both *N‐* or *C‐*tagged lines). Other core PB components were associated with the PM either very transiently (in dividing cells) or not at all (Appendix Fig S1A). We further observed that DCP1 can exhibit a polar behavior localizing at apical or basal domains of the PM, which was not observed for other core PB components (Fig 3C, lower right for polarity, and Appendix Fig S1). Moreover, the addition of cycloheximide (CHX), known to dissolve PBs as they are in dynamic equilibrium with polysomes (Gutierrez‐Beltran *et al*, 2015), decreased PB numbers and led to greater DCP1 localization at the PM (Appendix Fig S1B). By generating and using DCP1 tagged with the photoconvertible molecule EosFP to track *in vivo* DCP1 molecules (Wiedenmann *et al*, 2004; *UBQ10pro:EosFP‐DCP1*; photoactivatable green‐to‐red; Appendix Fig S1C caption for method details), we confirmed CHX results suggesting that DCP1 can shuttle between PBs and the PM (Appendix Fig S1C, and edges as described below). We cannot though discount the possibility that a fraction of DCP1 is targeted to the PM independently from PBs. Taken together, these results indicate that DCP1 localization at the PM is in a dynamic equilibrium with PBs.

**Figure 4:**
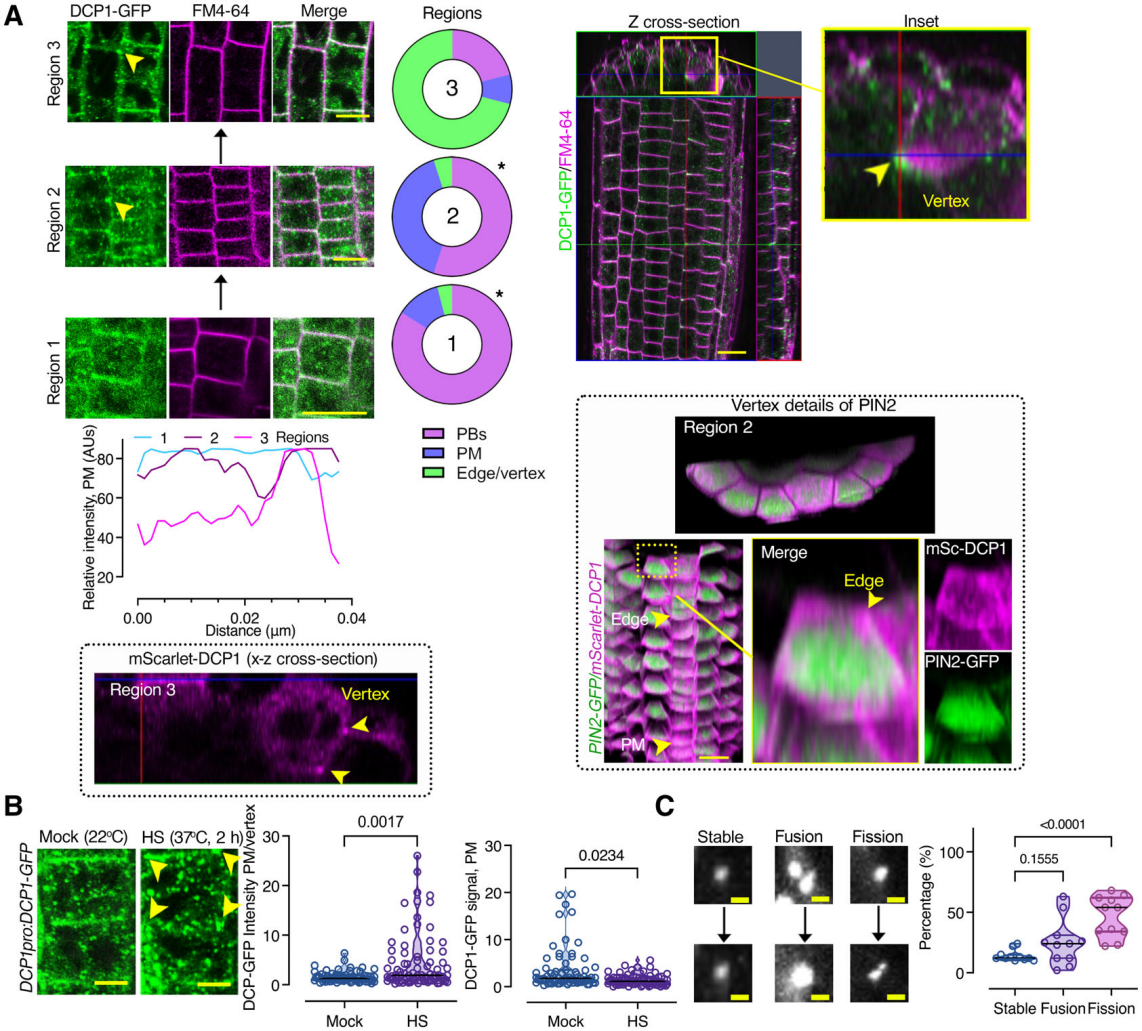
DCP1 protein accumulates at edges and then vertices during development Left: Gradual edge or vertex accumulation of DCP1‐GFP in three different root regions. DCP1 signal intensity among the three different regions, at the edge/vertex (epidermis). Right: z cross‐sectional images of DCP1‐GFP (green) in the whole root compared to FM4‐64 staining (magenta, staining membranes). The circular plots indicate the average DCP1 localization (regions 1–3; *N*, *biological replicates =* 3, *n* = 10 cells; comparing regions 1–2 and 2–3). The 3D‐rendered images (PIN2‐GFP vs. mSCarlet‐DCP1) show the localization of mScarlet‐DCP1 at edges/vertices in two different regions (in comparison to PIN2‐GFP signal which decorated almost evenly the PM). Scale bars: 20 μm. Arrowhead denotes the edge (region 2) and the vertex (region 3) decorated by mScarlet‐DCP1 (also in the z cross‐sectional image). Note in a single cell file, how the localization from the PM changes to the edge, as indicated, along the proximodistal axis.Representative confocal micrograph of DCP1‐GFP (*DCP1pro:DCP1‐GFP*, transgene) in root meristematic cells under NS/HS conditions (region 2). Scale bars, 5 μm. Note the depletion of DCP1 from the PM upon HS, but the increased edge/vertex signal (yellow arrowheads denote the vertex signal). Right: DCP1 signal intensity at the PM or edge/vertex (*N*, *biological replicates =* 3, *n* (*pooled data of 3 biological replicates*) = 18–23 PMs or edges/vertices).Representative confocal images showing fusion (coarsening), fission, and growth of PBs (DCP1‐positive) at the PM (region 3). Right: states of PBs (dynamic: fusion and fission and non‐dynamic: stable; *N*, *biological replicates =* 2, *n* (*pooled data of 3 biological replicates*) = 6–8 PBs). As a cautionary note, the “stable” PBs may not show dynamicity in the imaging time used (~ 3–5 min) but later, may do. Data information: In (A), *P < 0.05 was determined by a nested *t*‐test. In (B), *P* values were determined by the Kolmogorov–Smirnoff, while in (C) by one‐way ANOVA. Upper and lower lines in the violin plots when visible, represent the first and third quantiles, respectively, horizontal lines mark the median and whiskers mark the highest and lowest values. Source data are available online for this figure.

Notably, some cells accumulated more overall DCP1‐GFP at the PM than others, while in some cases we observed a spatial restriction of the signal at cell edges not seen for other PBs proteins or PIN2 (e.g., Fig 3C and Appendix Fig S1B). We use the term “edge” in the geometric sense of an intersection between two faces of a polyhedron, rather than of a periphery or front (Kirchhelle *et al*, 2016). This variability of localization prompted us to examine in detail in which cells DCP1 can be found on membranes, including the edges. To expedite the analyses here, we divided the roots into three regions (see also Fig 3A). Dynamic 3D imaging of DCP1‐GFP in these regions showed that the probability to find DCP1 at cell edges was higher far from the quiescent center (QC) along the proximodistal root axis (as defined in Fig 3A), where DCP1 can be further confined at the inner face of the PM at vertices (Fig 4A, e.g., in region 3). Like “edge”, the term “vertex” here is used in the geometric sense to define the internal angular point of a polygon and is defined as an intersection between the three cell edges. Under HS conditions, DCP1‐GFP abundance decreased at the PM at the expense of PBs but increased along the cell edges (Fig 4B). Our results suggested a dynamic competition between PBs and vertices exclusively for the DCP1 component.

As DCP1 localization at the edge or vertex was unaffected by the presence of CHX (Appendix Fig S1B, arrowhead), this result implied that DCP1 did not form decapping complexes at PM interfaces. To further validate this result, we used ultra‐fast live‐cell imaging super‐resolution microscopy showing that DCP1–decorated PBs undergo fission at the PM plane, probably to remodel PBs and release DCP1 (Fig 4C). This result is consistent with the diffusible loss of material from PBs associated with the PM observed by TIRF‐M (Fig 3B; at the same regions, 2 and 3) and likely implied that DCP1 dissociates from DCP2, although whether this dissociation is a prerequisite for the further loss of material requires further studies. Likewise, in animals, PBs undergo fission by the membranous ER sheets (Lee *et al*, 2020), but it is also unclear whether ER plays a role in splitting PBs at the PM. Accordingly, when we tested the dynamic interaction between DCP1 and DCP2, by sensitized emission Förster resonance energy transfer (SE–FRET), we did not detect DCP1–DCP2 interaction at the PM, vertex, or edge (Appendix Fig S2A). However, DCP1 did show a weak and transient association with the PBs core protein PAT1 at the PM (but not at the edge or vertex; Appendix Fig S2A). As the decapping complex comprises DCP1 and DCP2 as minimal components (Charenton *et al*, 2016), together with the CHX data, these results argued against the formation of a decapping complex at the PM or on edges/vertices.

**Figure 5:**
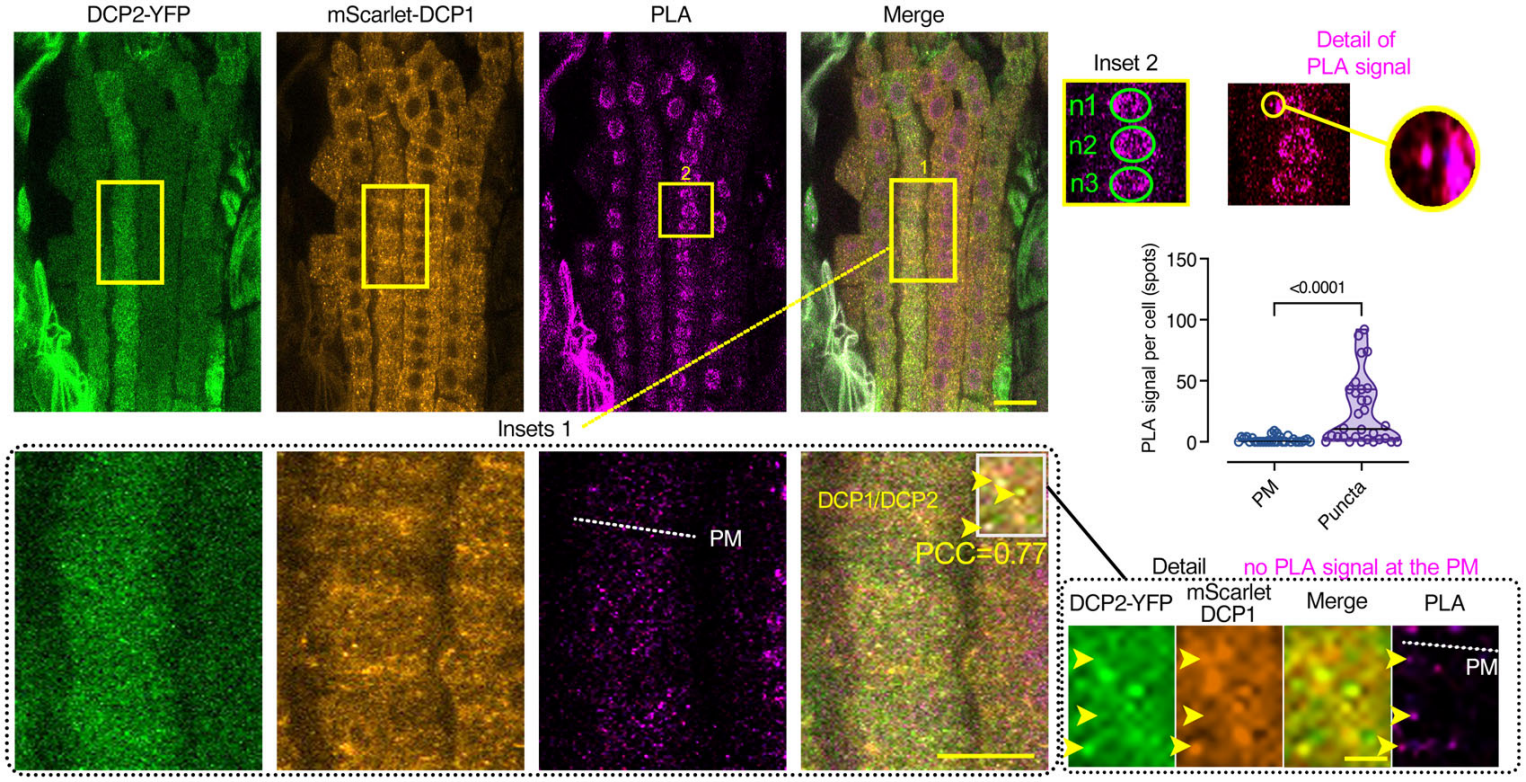
DCP1 interacts with DCP2 in a PLA assay occasionally in the cytoplasm but not at the PM Confocal micrographs showing single optical sections PLA‐assays producing signal that resembles spots. The antibodies used were anti‐FLAG/anti‐GFP detecting the *HF‐mScarlet‐DCP1/ DCP2‐YFP*, respectively (*RPS5apro:HF‐mScarlet‐DCP1* and *35Spro:DCP2‐YFP*). Inset 1: magnification showing the colocalization of PLA spots with DCP1 or DCP2 signals (colocalization and Pearson's correlation coefficient PCC value for the spots shown). The dotted white line in the PLA channel corresponds to the PM plane. Inset 2: positive PLA signal for DCP1 and DCP2 in the nucleus. “n1‐n3” correspond to nuclei regions (green circles). On the right, note the PLA spot nearby the nucleus (“detail of PLA signal”). The chart shows the quantification of PLA spots per cell at puncta (cytoplasm) or on the PM (*N*, *biological replicates =* 3, *n* (*pooled data of 3 biological replicates*) = 16–33 cells). As a cautionary technical note, the cytoplasmic, nuclear or PM “spots” do not connote physiologically relevant puncta, condensates, or PM clusters. Scale bar: 20 μm / 10 μm for the insets. Data information: In (A), *P < 0.05 was determined by one‐way ANOVA. Upper and lower lines in the violin plots when visible, represent the first and third quantiles, respectively, horizontal lines mark the median and whiskers mark the highest and lowest values.

To validate the lack of association between DCP1 and DCP2 at membranes, as FRET is sensitive to stoichiometry and may also fail to capture very transient interactions, we exploited the power of quantitative 3D proximity ligation assays (PLAs; Teale *et al*, 2021; preprint: Liu *et al*, 2022). PLA uses complementary oligonucleotides fused to specific antibodies to determine the frequency with which proteins of interest find themselves nearby (< 40 nm). When the proteins of interest interact or are nearby, PLA leads to a spot‐like signal (Appendix Fig S2B, left). In the PLA assay, DCP1 and DCP2 interacted or were nearby only in the cytoplasm but not at the PM (Fig 5). We also tested DCP1 against PAT1 (positive control) and the auxin efflux carrier PINFORMED7 (PIN7; negative control) to assess the reliability of PLA in *Arabidopsis* roots, with the prediction that fewer interactions should occur when target protein pairs have increasingly discrete accumulation domains (i.e., PIN7; absent in APEAL, Appendix Fig S2B and C). Unexpectedly, through this approach, we established that DCP1 and DCP2 form complexes also in the nucleus (Fig 5, inset 2), as was shown in yeast (*Saccharomyces cerevisiae*), where they act as a decapping reservoir (Tishinov & Spang, 2021). Together, PLA and SE–FRET analyses speak against the existence of a localized decapping activity at the PM. Furthermore, our analyses show that PLA assays can help in validating proxitomes.

### SCAR–WAVE recruits DCP1 at the cell edge/vertex

Given the reversible association of DCP1 with the PM (e.g., in HS), we postulated that DCP1 is not inserted there. We first tested whether DCP1 accumulates at the PM, edges, or vertices, through the secretory pathway by incubating seedlings with brefeldin A (BFA, 50 μM), a fungal toxin that prevents vesicle formation for exocytosis by inhibiting ADP ribosylation factor GEFs (ARF‐GEFs; Satiat‐Jeunemaitre *et al*, 1996). DCP1‐GFP did not accumulate intracellularly upon BFA treatment (Appendix Fig S3), and it lacks a signal peptide or a transmembrane domain, suggesting that it is not inserted in the PM and likely is not a secreted protein.

The accumulation of DCP1 during development at cell edges and vertices prompted us to examine the underlying mechanism by which DCP1 is recruited there. We postulated that proteins with which DCP1 interacts could regulate this localization. To this end, we compared DCP1 localization to that of other proteins that localize at cell edges/vertices and are enriched in the APEAL (i.e., SOSEKI3 [SOK3], which regulates a cellular coordinate geometric system (van Dop *et al*, 2020) with log_2_FC = 0.58 for NS and 2.0 for HS; and the SCAR–WAVE complex (Dyachok *et al*, 2008) that regulates actin nucleation). In addition, as a negative control, we used the edge‐localizing Ras‐related protein Rab‐A5c (Kirchhelle *et al*, 2019), which we did not identify by APEAL. SOK3 localized at the cell division zone (the cell plate fusion site), unlike DCP1, which was absent from this site (Appendix Fig S4A and B, region 1). Later in development, SOK3 showed some signal collinearity with DCP1 at edges/vertices (Pearson's correlation coefficient = 0.87, region 3). In contrast, Rab‐A5c showed little colocalization with DCP1 at all stages examined (Appendix Fig S4C). We further observed that neither the loss of SOK3 function (from a *sok3* mutant), the simultaneous loss of SOK1/SOK3 functions (from genome editing in the *sok3* mutant; details in Materials and methods), nor Rab‐A5c depletion (through a dominant‐negative dexamethasone‐inducible expression of inactive Rab‐A5c; Kirchhelle *et al*, 2016) resulted in changes in DCP1 localization at the PM or the edge/vertex (Appendix Fig S4B and C, in live imaging or through detection by α‐DCP1; details for the antibody in Materials and methods). These data suggested that neither SOK3 nor Rab‐A5c recruit DCP1 at vertices or edges.

**Figure 6:**
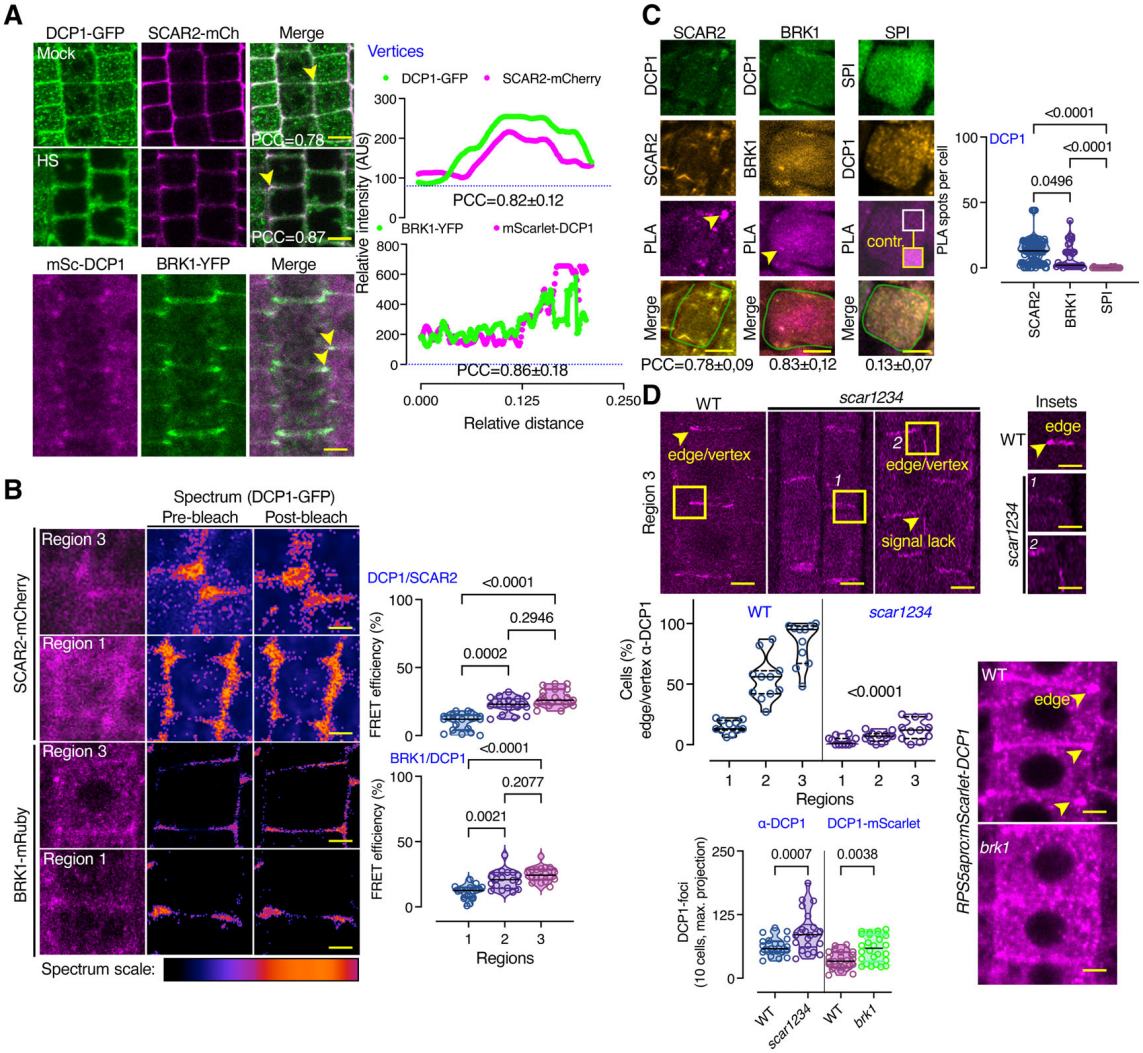
DCP1 cooperates with the SCAR–WAVE complex DCP1 colocalizes with the SCAR–WAVE components SCAR2 and BRK1. Representative confocal micrographs showing colocalization between DCP1 and SCAR2 or DCP1 and BRK1 (in lines coexpressing *DCP1pro:DCP1‐GFP* and *SCAR2pro:SCAR2‐mCherry* or *RPS5apro:HF‐mScarlet‐DCP1* and *BRK1pro:BRK1‐YFP*; arrowheads indicate colocalization at cell edges or vertices) in root epidermal cells (regions 2–3). Right: relative signal intensity profiles of DCP1 or SCAR2 at vertices. Colocalization at these regions was also calculated (PCC; *N*, *biological replicates =* 2, *n* = 4–10 edges/vertices in regions 2–3; a slight increase was observed in HS, 0.78 in NS vs. 0.87 in HS). Scale bars: 10 μm.Representative confocal micrographs with acceptor photobleaching‐FRET efficiency between SCAR2‐mCherry (up) or BRK1‐mRuby (down) and DCP1‐GFP (epidermal cells, regions 1 and 3). Right: normalized FRET efficiency between SCAR2 or BRK1 and DCP1, respectively, among the different developmental root regions (*N*, *biological replicates =* 2, *n* = 16 cells). Scale bars: 3 μm.Representative confocal micrographs showing PLA spots produced by α‐GFP/α‐RFP in *DCP1‐GFP/SCAR2‐mCherry* lines and *DCP1‐GFP/BRK1‐mRuby* lines, α‐FLAG/α‐GFP in *HF‐mScarlet‐DCP1/SPI‐YPet* (SPIRRIG [SPI] is a negative control as it localizes to PBs only during salt stress and was not found in the APEAL). In the SPI PLA, a high contrast inset is presented. Right: number of PLA spots per cell (*N*, *biological replicates =* 3, *n* (*pooled data of 3 biological replicates*) = 14–33 cells). In the merged images, the cell contours are shown (light green transparent). Scale bars: 5 μm.Representative confocal micrographs showing DCP1 localization detected by α‐DCP1 in the wild type (WT) or the *scar1 scar2 scar3 scar4* (*scar1234*) quadruple mutant or in live‐cell imaging of mScarlet‐DCP1 (*RPS5apro:HF‐mScarlet‐DCP1*) in WT or *brk1* mutant (bottom right; epidermal cells, region 3 for α‐DCP1 and 2 for live‐cell imaging). The arrowheads denote the lack of robust DCP1 localization in *scar1234* at the edge/vertex. Small panels (insets) at right show details corresponding to the regions delineated by dashed lines, where arrowhead denotes the edge signal of DCP1 in WT; scale bars, 1 μm. Bottom: percentage of cells with proper edge/vertex localization and quantification of PB numbers (DCP1‐foci; *N*, *biological replicates =* 3, *n* (*pooled data of 3 biological replicates*) = 18–35 cells). Scale bars, 5 μm. Data information: In (B), *P* values were determined by Kruskal–Wallis, and the comparisons are among the *scar1234* to the corresponding WT samples. In (C and D), *P* values were determined by Wilcoxon. PCCs are means ± s.d. Upper and lower lines in the violin plots when visible, represent the first and third quantiles, respectively, horizontal lines mark the median and whiskers mark the highest and lowest values. Source data are available online for this figure.

In sharp contrast with Rab‐A5c or SOK3, we observed almost perfect signal collinearity at vertices/edges for mCherry and GFP signals in cells co‐expressing *SCAR2‐mCherry* (with log_2_FC = 2.32 for NS and 3.62 for HS, respectively) and *DCP1‐GFP* or *BRK1‐YFP* (BRICK1; components of the SCAR–WAVE complex; Dyachok *et al*, 2008) with *mScarlet‐DCP1* (Fig 6A). The SCAR2 signal though at the cell plate was weak and we were unable to draw conclusions about colocalizations there. Of note, other SCAR–WAVE components like PIR121/SRA1, NAP1, and GRL/NAP125 were also highly enriched in PDL datasets, especially upon HS (log_2_FC ≥ 2 for NS and 3.5 for HS; Fig EV5 and Source Data 7). Accordingly, upon HS, as DCP1 association with the PM decreased, we observed a stronger colocalization at edges/vertices between SCAR2 and DCP1 (Fig 6A). This result explains well the increase in DCP1 signal at edges/vertices in HS and the APEAL results suggesting a stronger association of SCAR–WAVE/DCP1 under HS.

We also investigated whether the colocalization between DCP1 and SCAR–WAVE, reflected an interaction. Indeed, DCP1 and SCAR2, or DCP1 and BRK1, interacted in FRET assays in edges/vertices but not in PBs (Fig 6B, region 3). To ascertain these interactions, we used the quantitative 3D PLA assay (Fig 6C). PLA determined that DCP1 and SCAR–WAVE components mainly interact at edges/vertices, although we occasionally observed PLA spots in the cytoplasm, where SCAR–WAVE can also be found (Wang *et al*, 2016). We note here that AP step of the APEAL failed to retain the interaction between DCP1 and SCAR–WAVE, indicating that co‐immunoprecipitation experiments are ineffective in this case (Source File 5). These results are specific, as DCP1 failed to produce positive PLA spots when combined with SOK3 at the PM or with SPIRRIG (SPI), a protein that associates with SCAR–WAVE in root hairs or with PBs only during salt stress (Steffens *et al*, 2015; Fig 6C and Appendix Fig S4A).

Furthermore, in the quadruple loss‐of‐function mutants *scar1234* and *brk1* that show a lack of SCAR–WAVE activity (Djakovic *et al*, 2006; Dyachok *et al*, 2008; Chin *et al*, 2021), DCP1 localization at the edge/vertex was almost completely lost in both a live imaging setting or through detection by α‐DCP1 (Fig 6D). In *scar1234* and *brk1*, the DCP1 localization domain size at edges/vertices was expanded, in the few cells that DCP1 localized there (Fig 6D), suggesting that SCAR–WAVE is required for the confinement of DCP1 at edges/vertices. Furthermore, this reduced DCP1 localization at edges/vertices in *scar1234* and *brk1* was associated with an increase in the number of PBs (Fig 6D), further confirming the competition for DCP1 between PBs and membranes.

SCAR–WAVE activates the ARP2–ARP3 complex to nucleate actin through polymerization and the organization of filaments into y‐branched networks (Huang *et al*, 2019). ARP2–ARP3 complex components were slightly enriched in the APEAL datasets, again mainly under HS like the SCAR–WAVE components likely due to the local restriction of ARP2–ARP3 there (log_2_FC ~ 1.8 for HS; Fig EV5). The major ARP2–ARP3 component ARPC5 expressed as a tagRFP fusion (the smallest subunit of the ARP2–ARP3 complex), colocalized partially with DCP1 and showed a significant interaction with it in FRET only at vertices (Appendix Fig S5A). Notably, the loss of ARP2–ARP3 function (in the “crooked” mutant allele), *arpc5* (Pratap Sahi *et al*, 2017) or the ARP2–ARP3 inhibitor CK‐666, did not deplete DCP1 from the vertex/edge, but rather led to a more variable DCP1 localization domain size at edges/vertices (Appendix Fig S5B). These results suggested that SCAR–WAVE may be involved in the recruitment of DCP1 molecules at the vertex, while the SCAR–WAVE effector ARP2–ARP3 may contribute to the confinement of the SCAR–WAVE–DCP1 at vertices.

### DCP1 Phosphostatus defines its localization at the edge or vertex where it regulates Actin remodeling

**Figure 7:**
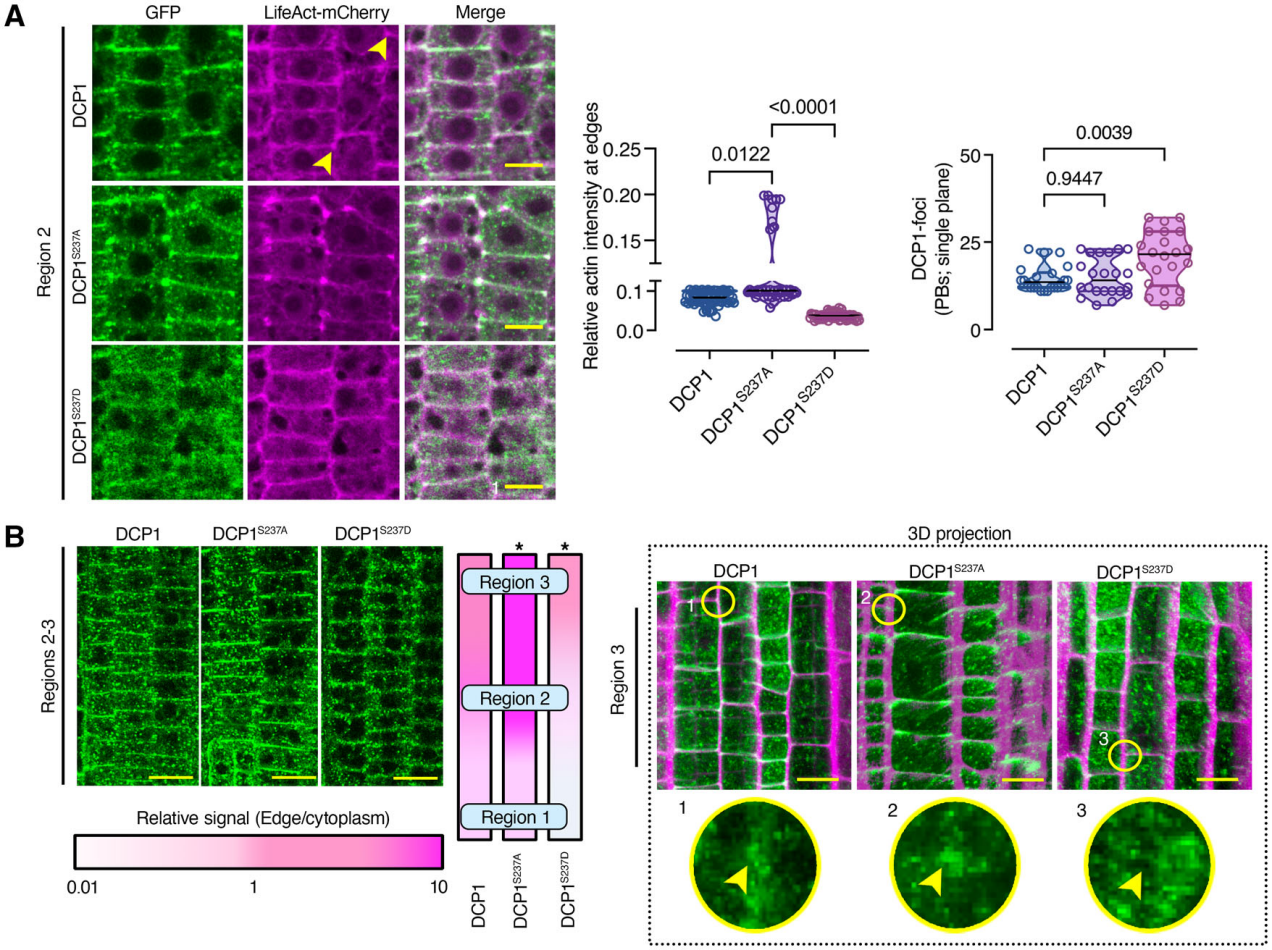
DCP1 Phosphostatus defines its localization at the edge or vertex where it regulates Actin remodeling Representative confocal micrographs showing colocalization between DCP1 or two DCP1 phosphovariants with LifeAct‐mCherry in lines co‐expressing *DCP1pro:DCP1‐GFP* (or variants) and *UBQ10pro:LifeAct‐mCherry* (cell edges are indicated by yellow arrowheads). Right: relative signal intensity of actin at cell edges/vertices (normalized to the PM) in epidermal cells (*N*, *biological replicates =* 3, *n* = 15–30, regions 2–3). Scale bars: 7 μm.Representative high‐resolution confocal micrographs showing the localization of DCP1‐GFP or phosphovariants (regions 2–3, epidermal cells). Left: signal at the vertex, expressed as a color‐coded edge/cytoplasmic signal ratio. Right: representative 3D projection from super‐resolution (120 nm axial, FM4‐64 counterstaining of PM) images of root meristematic cells captured from *DCP1pro:DCP1‐GFP* and phosphovariants (*N*, *biological replicates = 4*, *n = 8*). Circular insets show the differential vertex localization of DCP1 (absent in DCP1^S237D^‐GFP line), and arrowheads denote the vertex. Note the enhanced accumulation of DCP1^S237A^‐GFP at the vertex. Scale bars: 15 μm. Data information: In (A), *P* values were determined by Kruskal–Wallis, while in (B), *P < 0.005 by ordinary one‐way ANOVA relative to unmutated DCP1. Upper and lower lines in the violin plots when visible, represent the first and third quantiles, respectively, horizontal lines mark the median and whiskers mark the highest and lowest values. Source data are available online for this figure.

As DCP1 interacted with SCAR–WAVE and ARP2–ARP3, we aimed at testing whether DCP1 might affect actin remodeling. DCP1 showed almost perfect signal collinearity with cortical F‐actin (decorated by the *UBQ10pro:LifeAct‐mCherry*; Fig 7A, top). The actin‐depolymerizing drugs cytochalasin D or latrunculin B strongly enhanced DCP1 localization at edges or vertices (Appendix Fig S6, *P <* e^−4^), suggesting that actin does not recruit DCP1 there. In contrast, treatment with the microtubule (MT)‐depolymerizing drug amiprophos‐methyl (APM; Riedl *et al*, 2008) led to a more variable DCP1 domain size at the edge/vertex (Appendix Fig S7A). Furthermore, MTs were depleted from DCP1‐rich vertices, while the actin signal was enhanced (Appendix Fig S7B and C). Intriguingly, in the apex of trichome branches, a similar MT‐depletion zone confines ARP2–ARP3 and actin (Yanagisawa *et al*, 2018), implying that narrow membrane domains tend to accumulate these complexes. Whether DCP1 accumulates in the trichome apex remains to be shown. Although MTs affected DCP1 confinement at edges/vertices where actin accumulated, we opted against using MTs as a tool to affect DCP1 localization, as this could lead to pleiotropic effects.

**Figure 8:**
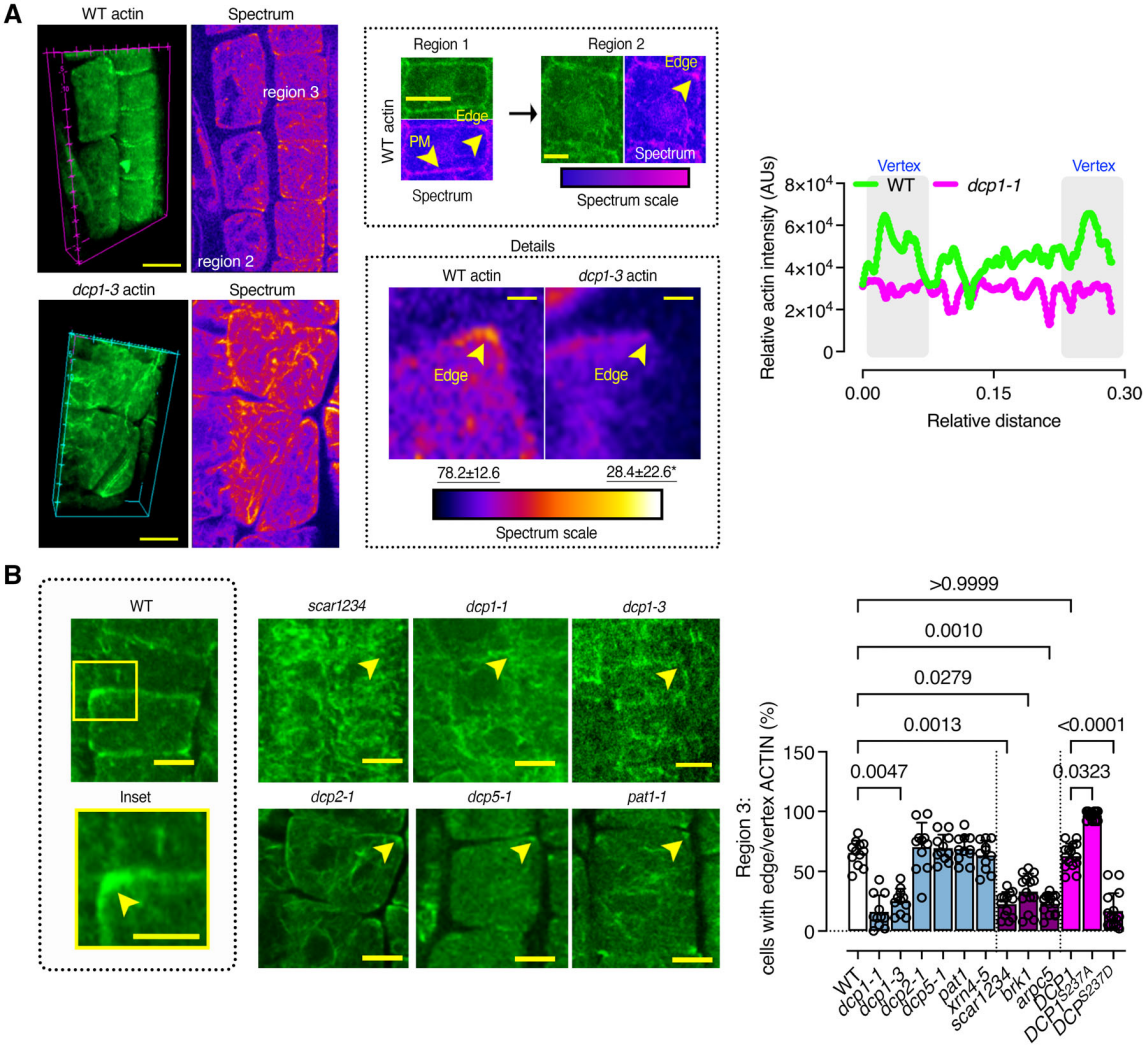
DCP1 Phosphostatus regulates Actin remodeling at the edge Representative confocal 3D rendering micrographs of root meristematic cells from the WT or the *dcp1‐3* mutant stained with phalloidin for actin visualization (*N*, *biological replicates = 3*, *n = 4*). Scale bars: 5 μm (*z*‐scale is 4 μm). Upper middle: the “Spectrum” micrographs indicate the maximum color‐coded signal intensity (scale on the right, middle inset). Note that the signal is evenly distributed in region 1 (left upper micrograph), whereas it mostly accumulates at the edge or vertex in regions 2 and 3 (see arrowheads; images on top). Lower middle: a detail of the higher actin accumulation at edges/vertices in region 2 (compare regions 1 and 2, Scale bars: 7 μm). Insets (details, Scale bars: 2 μm) indicate the loss of vertex actin accumulation in *dcp1‐3*. Right: plot profile from the actin signal in the WT or *dcp1‐3*. The vertices are indicated.Representative confocal micrographs showing actin localization in WT, *dcp1‐1*, *dcp1‐3*, *scar1234*, *dcp2‐1*, *dcp5*, and *pat1* upon phalloidin staining and graph (right) indicating the percentage of cells in region 3 with an accumulation of actin at edges in various genotypes (*N*, *biological replicates =* 3, *n* = 7–9 roots, bars show means + s.d.). Scale bars: 7 μm. Data information: In A, *P* values were determined by Kruskal–Wallis, while in (B), the exact *P* values were determined by Brown–Forsythe and Welch ANOVA. Source data are available online for this figure.

We thus looked for another finer approach to affect DCP1 localization at the cell edge. As the phosphorylation of residue Ser237 of DCP1 modulates its function in PBs (Yu *et al*, 2019), we asked whether DCP1 phosphorylation status might also modulate DCP1 abundance at the edges/vertices. We thus introduced a construct expressing the non‐phosphorylatable variant *DCP1* ^*S237A*^ *‐GFP* in *dcp1‐1* (Yu *et al*, 2019), a stronger allele than *dcp1‐3*, under the control of the *DCP1* promoter. We noticed an earlier and increased accumulation of fluorescence at edges/vertices in the resulting transgenic plants, compared to *DCP1pro*:*DCP1‐GFP* in the *dcp1‐1* (Fig 7A and B). Conversely, the introduction of the phosphomimetic variant *DCP1* ^*S237D*^ *‐GFP* in *dcp1‐1* showed a prevalent localization to PBs, alongside a pronounced inability to localize in a timely fashion to cell edges/vertices (Fig 7A and B). In the lines expressing *DCP1* ^*S237D*^, F‐actin largely failed to accumulate at the cell edges/vertices but not on PM (Fig 7A), whereas *DCP1* ^*S237A*^ exerted the opposite effect, enhancing actin restriction at the edge/vertex (Fig 7A). We confirmed that the *scar1234*, *dcp1‐1* and *dcp1‐3* mutants all display a similar lack of actin accumulation at edges/vertices, further demonstrating that actin restriction at edges/vertices increases along the developmental root axis (Fig 8Α, region 1 vs. 3). On the other hand, the core decapping mutants (e.g., *dcp2‐1*, *dcp5‐1*, and *pat1*) did not display this phenotype (Fig 8Β, right panel). Altogether, these data establish that the SCAR–WAVE–DCP1 pathway controls actin at edges/vertices in a process that can be modulated by the phosphorylation status of DCP1 Ser237 and independently of decapping.

### Mutual potentiation of DCP1 and SCAR‐WAVE localization at the edge/vertex

**Figure 9:**
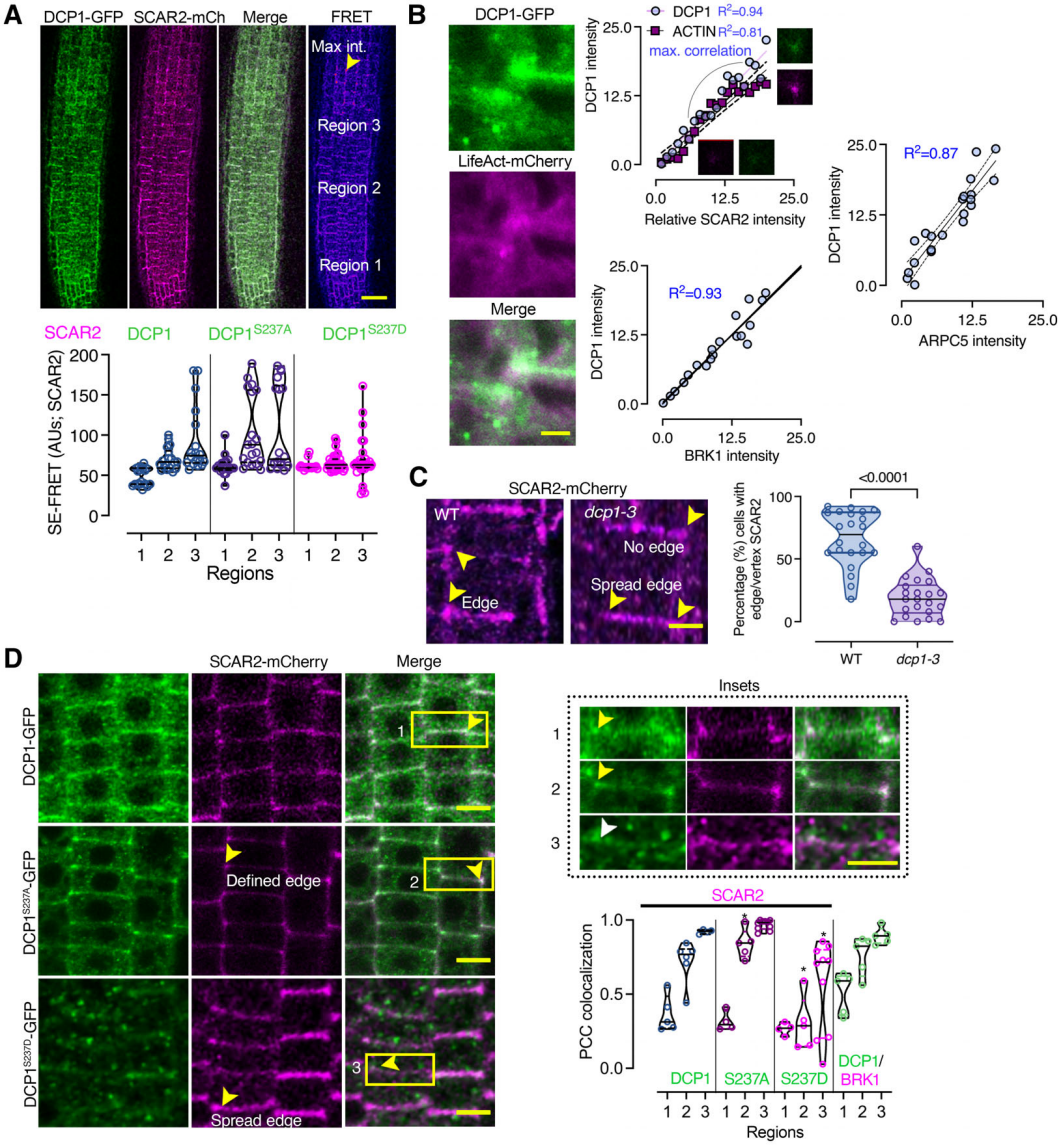
Mutual potentiation of DCP1 and SCAR‐WAVE localization at the edge/vertex SE‐FRET efficiency between DCP1‐GFP or its phosphovariants with SCAR2‐mCherry (among the three different root regions; mainly epidermal cells). Scale bar: 50 μm. The arrowhead denotes high FRET efficiency at edges/vertices of region 3. Right: signal quantification of SE‐FRET efficiency between the indicated combinations at the epidermis of 3 regions (*N*, *biological replicates =* 6, *n* (*pooled data of 3 biological replicates*) = 10).Actin nucleation site at an edge/vertex, as indicated by DCP1‐GFP and LifeAct‐mCherry localization. Right: correlation between DCP1 intensity, ACTIN, SCAR2, BRK1 and ARPC5 intensities (simple regression model). The *R* ^2^ values are shown, along with representative micrographs for DCP1/SCAR2 (*N*, *biological replicates =* 3, *n* = 6 for each point).Representative confocal micrographs showing SCAR2‐mCherry localization in WT and *dcp1‐3* mutant, respectively (root region 2, epidermal cells) and quantification of edge/vertex with SCAR2 a confined signal (*N*, *biological replicates =* 3, *n* = 5–8 cells).Representative confocal micrographs showing the colocalization between DCP1‐GFP or phosphovariants and SCAR2‐mCherry in root meristematic cells (root region 2, epidermal cells). Scale bars: 10 μm. The insets show details of colocalization; the white arrowhead denotes the expansion of the SCAR2/DCP1 domain, while the yellow arrowheads the restricted edge/vertex SCAR2/DCP1 domains. Scale bars: 3 μm. The graph indicates the relative signal intensity for the indicated combinations (as Pearson's correlation coefficient; *N*, *biological replicates =* 3, *n* = 5 at edges/vertices: spread edges were not considered in calculations). Data information: In (A and B), *P < 0.005 were determined by nested one‐way ANOVA relative to the WT in the respective region. In (B), a simple linear regression (best‐fitted model) with a 95% confidence interval is shown with dashed lines. In (C and D), *P* values were determined by an unpaired *t*‐test. Upper and lower lines in the violin plots when visible, represent the first and third quantiles, respectively, horizontal lines mark the median and whiskers mark the highest and lowest values. Source data are available online for this figure.

As DCP1 levels and phosphostatus at the vertex correlate well with actin nucleation, we asked if DCP1 reciprocally can affect SCAR–WAVE and ARP2–ARP3 levels there. Accordingly, we conducted FRET assays using either acceptor photobleaching or sensitized emission, along the proximodistal root axis between SCAR2 and DCP1 phosphovariants (Figs 6B and 9A). As expected, due to the increased colocalization between DCP1^S237A^ and SCAR2, DCP1^S237A^‐GFP exhibited increased FRET with SCAR2‐mCherry, and a faster response to the developmental increment, unlike DCP1^S237D^ (Fig 9A and C). Importantly, using signal regression analyses we managed to fit a simple regression model and observed a good correlation between DCP1 levels, SCAR2, BRK1, ARPC5, and actin signal intensity at the vertex (Fig 9B, *R* ^2^ > 0.80). Remarkably, while DCP1^S237D^‐GFP could still localize at the edge/vertex (albeit later than the WT; ~ 30 μm along the root axis, see also 6B quantifications), showed an expansion of the SCAR2 and ARPC5 domains (Fig 9C, insets, and Appendix Fig S8). As expected, in *dcp1‐3* due to the reduced levels of DCP1, the SCAR2 signal decreased at the vertex (Fig 9C). Furthermore, in DCP1^S237A^ the ARPC5 increased at the edges, in contrast to DCP1^S237D^ which showed less recruitment of ARPC5 there (Appendix Fig S8). Hence, while SCAR–WAVE recruits DCP1 at edges/vertices, DCP1 reinforces SCAR–WAVE/ARP2–ARP3 localization which may promote actin remodeling and nucleation.

### The SCAR–WAVE–DCP1 nexus at the vertex can modulate growth anisotropy

**Figure 10:**
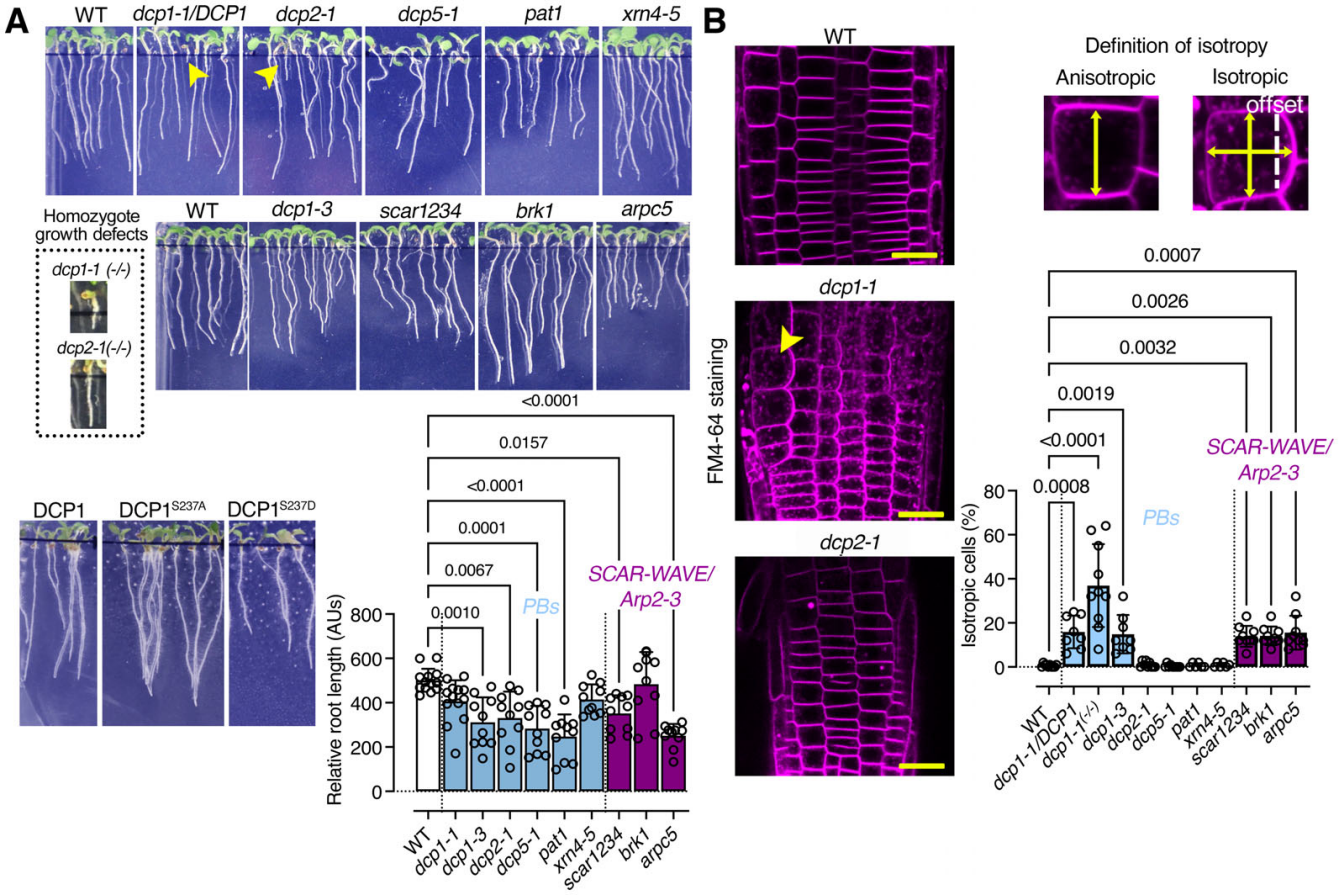
The SCAR–WAVE‐DCP1 nexus at the vertex can modulate growth anisotropy Representative images showing the phenotypes of *dcp1* mutants and mutants in other PB core components or SCAR–WAVE components (5‐day‐old seedlings). The arrowheads show the growth defects of homozygous *dcp1‐1* or *dcp2‐1* mutants (denoted −/−; heterozygous denoted *dcp1‐1*/DCP1; details are also shown). Lower: graph showing relative root length (*N*, *biological replicates =* 3, *n* (*pooled data of 3 biological replicates*) = 3–4 roots, bars show means + s.d).DCP1 regulates cell expansion anisotropy. Representative confocal micrographs showing FM4‐64 staining of the WT, *dcp1‐1* and *dcp2‐1* mutants (2 μM, 10 min). Scale bars, 20 μm. Right: percentage of isotropic cells per root meristem (%, epidermal cells) in each genotype (*N*, *biological replicates =* 3, *n* (*pooled data of 3 biological replicates*) = 3–5 roots, bars show means ± s.d). Examples of isotropic or anisotropic cells are shown, along with the developmental axis offset at the *x*‐ and *y*‐axes. Data information: In (A and B), *P* values were determined by ordinary one‐way ANOVA (Kruskal–Wallis produced similar results, with Dunn's or FDR corrections). Source data are available online for this figure.

The SCAR–WAVE/ARP2–ARP3 module specifies leaf pavement cell shape and trichome development, light‐dependent and auxin‐dependent root growth, stomatal opening, gravitropism, salt stress responses and immunity (Chin *et al*, 2021 and references therein). We thus explored the possible consequences of the SCAR–WAVE–DCP1 interaction, considering also that edges are likely associated with growth anisotropy (Kirchhelle *et al*, 2019). Anisotropy, in terms of differential growth, is the relative change in principal dimensions over time, for example, the young hypocotyl elongates more than it widens (Kirchhelle *et al*, 2016). SCAR–WAVE regulates growth patterns by impinging on cell wall properties at sharp cell edges or apexes (e.g., roots or trichome; Dyachok *et al*, 2008; Wang *et al*, 2019). We thus asked whether DCP1 or SCAR–WAVE mutants showed defects in their anisotropic expansion. To attenuate the known growth perturbations of SCAR–WAVE mutants and focus on anisotropy and not on pleiotropic growth defects, we used vertically grown plates with a high concentration of gelation agent (1.5% [w/v] vs. 0.5% gelrite), as described previously (Dyachok *et al*, 2011). The roots of seedlings expressing *DCP1* ^*S237D*^, or the progeny from *dcp1‐1/DCP1* plants (as the homozygote cannot survive past the early seedling stage), and of the *dcp1‐3* mutant, had slightly shorter roots than the WT (Fig 10A). However, seedlings expressing *DCP1* ^*S237A*^ had longer roots than WT seedlings (Fig 10A). Albeit with mild developmental defects, *dcp1‐1* exhibited a significant loss of anisotropy like that seen in the *scar1234*, *brk1*, or *arpc5* mutants (Fig 10B). This phenotype correlated well with actin accumulation at edges, as *dcp2‐1* (including the homozygous mutant, inset in Fig 10A), *dcp5‐1*, *pat1‐1*, *or xrn4‐5* or mutants lacked similar defects in anisotropy, although they did exhibit reduced overall growth (Fig 10A and B). These results suggested that SCAR–WAVE–DCP1 regulates anisotropy likely through actin.

To consolidate the link between DCP1 and anisotropy, we used isoxaben, a cellulose biosynthesis inhibitor that promotes isotropic growth (Tateno *et al*, 2015). Long treatments with isoxaben increased the accumulation of DCP1‐positive PBs (Appendix Fig S9A), perhaps by activation of a stress‐related pathway linked to cell wall integrity, so we determined a concentration and incubation time resulting in minimal effects on DCP1 localization for the following experiments (either 10 μM for 8 h or 40 μM for 1 h). Under this setting, isoxaben induced a marked isotropic cell expansion in DCP1^S237D^ cells mutants compared to WT, in contrast to DCP1^S237A^ which were more tolerant to this treatment (Appendix Fig S9B, note the isotropy graph). Furthermore, isoxaben redistributed SCAR–WAVE and DCP1 in a similar and expanded domain before isotropy and radial growth took place (Appendix Fig S9B). Notably, the DCP1^S237A^ cells elongated faster than those of the WT (Appendix Fig S9B, length graph). If DCP1 did not have a function in isotropy, one would expect DCP1^S237A^ cells to simply swell more due to higher expansion propensity, which was not the case (Appendix Fig S9B). Because of this restriction of the radial growth by DCP1^S237A^, this result along with the observed defects of anisotropy in *scar1234*, *arpc5*, and *dcp1* mutants support the notion that the SCAR–WAVE–DCP1 link regulates, drives, and targets expansion anisotropy.

## Discussion

We propose a framework describing how the composition of a cellular condensate (PBs here) can be determined and use up this information to delineate new pathways, such as those relevant to growth. We further exemplify how a condensate is dissolved at an unappreciated membrane interface and how a fraction of the condensate can be repurposed as a cellular coordinate geometric system through the formation of another condensate (i.e., the SCAR–WAVE–DCP1 link). Unlike other coordinate systems like that driven by SOSEKI that appears important early in development (van Dop *et al*, 2020), the system suggested herein might be important later during development to regulate anisotropy and directional expansion.

The plasticity of PBs due to the inherent properties of condensates that depend on weak interactions allows a dynamic competition between PBs and membrane surfaces for the same components (e.g., DCP1). The subcellular positioning of DCP1 at certain membrane surfaces, and particularly at vertices, may be instrumental in driving phase transitions (i.e., phase separation), by further reducing the radius within which proteins can diffuse, thereby promoting condensation (Freeman Rosenzweig *et al*, 2017). This mechanism may further expand our comprehension of how condensation is promoted by reducing diffusion dimensionality (3D to 2D) from the cytoplasm to the plane of membranes. The dynamic spatial restriction of condensation also influences the material states of condensates in neurons (Gopal *et al*, 2017). Material state transitions (e.g., liquid‐to‐solid) of condensates depend on post‐translational modifications, raising the intriguing possibility that a combination of vertex confinement for DCP1 (reduced diffusion and spatial restriction), together with DCP1 dephosphorylation, may entropically favor transitions that stabilize SCAR–WAVE–DCP1.

Moreover, cell edges are sites of actin nucleation in plants (Ambrose *et al*, 2011 and results herein), making the link between DCP1 and SCAR–WAVE highly relevant. Similarly, in animal cells, LLPS promotes the clustering of receptors with WASP to potentiate the ARP2–ARP3 complex assembly at the PM for an efficient downstream signaling amplification (Case *et al*, 2019). In animal cells, many actin‐nucleation‐promoting factors, such as WHAMM (WASP homolog‐associated protein with actin, membranes, and microtubules), JMY (Junction Mediating And Regulatory Protein), the WASH complex, and the SCAR–WAVE complex, can activate ARP2–ARP3 (Wang *et al*, 2019). However, of the above list, only the SCAR–WAVE complex has been identified in plants thus far. Plants may thus employ other regulators to fulfill their needs for SCAR–WAVE condensation and actin nucleation. Vertices may therefore promote the activation of SCAR–WAVE through DCP1.

There are also some thought‐provoking parallels between plant edge/vertex condensation and animal epithelial sheets, where tight junctions (showing geometric similarities to vertices) are formed by zonula occludens condensates which maintain epithelial functions (Bosveld *et al*, 2018; Beutel *et al*, 2019). Notably, although the edge‐decorating plant SOKEKI proteins contain a PDZ domain (also known as DHR domains or GLGF repeats) that is also associated with tight junctions (Beutel *et al*, 2019), we did not find at the moment links to SOSEKIs, whose exact functions remain to be determined. On the other hand, the links to actin nucleation are more solid, although further studies are needed to delineate the exact molecular mechanism by which SCAR–WAVE–DCP1 modulates actin dynamics.

Perhaps the most puzzling contradiction in our data is the variable edge/vertex decoration by SCAR–WAVE or DCP1. The lack of robustness of this process may relate to a seemingly stochastic condensation of SCAR–WAVE–DCP1 at regions closer to the QC, which can bring about local asymmetries in anisotropy at the cellular level. We accordingly show that cells with edges/vertices well defined by this complex follow a highly predictable anisotropic growth pattern, while cells with less determined SCAR–WAVE–DCP1 vertices have more diffusible growth patterns. Symmetry breakage, therefore, may entail a randomized condensation step that can bring about local asymmetries. Intriguingly, such cellular asymmetries underpin symmetries at the tissue level (Bou Daher *et al*, 2018). Lastly, feedback between the cell wall and SCAR–WAVE–DCP1 may add more complexity to this system. We thus cannot discount links between SCAR–WAVE–DCP1 and the cell wall, which can rigidify the rich in pectin middle lamella, the region between tricellular junctions (see also Fig 2C, pectin). The ARP2–ARP3 may also play a role in this context by transducing cell wall changes, as branched actin networks could be mechanosensitive (Papalazarou & Machesky, 2021).

## Materials and Methods

### Reagents and Tools table

**Table:** 

Reagent/Resource	Reference or Source	Identifier or Catalog Number
**Experimental Models**		
One Shot® ccdB Survival™ 2 T1R Competent Cells	ThermoFisher Scientific	A10460
Subcloning Efficiency DH5alpha chemically competent cells	ThermoFisher Scientific	18265‐017
NEB® 10‐beta Competent E. coli	New England BioLabs	C3019I
NEB® 5‐alpha F'Iq Competent E. coli (High Efficiency)	New England BioLabs	C2992I
Agrobacterium tumefaciens GV3101	N/A	N/A
*Arabidopsis thaliana* Columbia	35Spro:sGFP‐TurboID‐HF/WT	Arora *et al* (2020)
*Arabidopsis thaliana* Columbia	35Spro:DCP1‐TurboID‐HF/WT	This paper
*Arabidopsis thaliana* Columbia	35Spro:GFP‐DCP1/WT	Gutierrez‐Beltran *et al* (2015)
*Arabidopsis thaliana* Columbia	DCP1pro:DCP1‐GFP/*dcp1‐1*	Yu *et al* (2019)
*Arabidopsis thaliana* Columbia	DCP1pro:DCP1^S237A^‐GFP/*dcp1‐1*	Yu *et al* (2019)
*Arabidopsis thaliana* Columbia	DCP1pro:DCP1^S237D^‐GFP/*dcp1‐1*	Yu *et al* (2019)
*Arabidopsis thaliana* Columbia	RPS5apro:Arpc5‐tagRFP/DCP1‐GFP	This paper
*Arabidopsis thaliana* Columbia	RPS5apro:Arpc5‐tagRFP/DCP1^S237A^‐GFP	This paper
*Arabidopsis thaliana* Columbia	RPS5apro:Arpc5‐tagRFP/DCP1^S237D^‐GFP	This paper
*Arabidopsis thaliana* Columbia	*dcp1‐1*	GABI‐844B03 (Hoffmann *et al*, 2022)
*Arabidopsis thaliana* Columbia	*dcp1‐3*	SAIL_377_B10 (Hoffmann *et al*, 2022)
*Arabidopsis thaliana* Columbia	*dcp2‐1*	SALK_000519.52.10.x (Hoffmann *et al*, 2022)
*Arabidopsis thaliana* Columbia	*dcp5‐1*	SALK_008881 (Hoffmann *et al*, 2022)
*Arabidopsis thaliana* Columbia	*xrn4‐5*	SAIL_681_E01 (Hoffmann *et al*, 2022)
*Arabidopsis thaliana* Columbia	*pat1‐1*	SALK_040660 (Roux *et al*, 2015)
*Arabidopsis thaliana* Columbia	*arpc5*	SALK_123936.41.55 (Pratap Sahi *et al*, 2017)
*Arabidopsis thaliana*	*scar1234*	Dyachok *et al* (2008), Chin *et al* (2021)
*Arabidopsis thaliana* Columbia	*brk1*	Dyachok *et al* (2008), Chin *et al* (2021)
*Arabidopsis thaliana* Columbia	*sok1 sok3*	CRISPR double mutant/this paper
*Arabidopsis thaliana* Columbia	SCAR2pro:SCAR2‐mCherry	Chin *et al* (2021)
*Arabidopsis thaliana* Columbia	BRK1pro:BRK1‐mRuby3	Chin *et al*, 2021
*Arabidopsis thaliana* Columbia	BRK1pro:BRK1‐YFP	Chin *et al*, 2021
*Arabidopsis thaliana* Columbia	UBQ10pro:Lifeact‐mRuby	Chin *et al*, 2021
*Arabidopsis thaliana* Columbia	UBQ10pro:Lifeact‐mCherry	Chin *et al* (2021)
*Arabidopsis thaliana* Columbia	PAT1pro:PAT1‐GFP	Roux *et al* (2015)
*Arabidopsis thaliana* Columbia	SOK3pro:SOK3‐YFP	van Dop *et al* (2020)
*Arabidopsis thaliana* Columbia	35Spro:DCP2‐YFP	Jang *et al* (2019)
*Arabidopsis thaliana* Columbia	VCSpro:VCS‐GFP	Roux *et al* (2015)
*Arabidopsis thaliana* Columbia	UBQ10pro:DCP5‐GFP	Chicois *et al* (2018)
*Arabidopsis thaliana* Columbia	DEX˃RAB‐A2c^DN^	Kirchhelle *et al* (2016)
*Arabidopsis thaliana* Columbia	RAB‐A5cpro:RAB‐A5c	Kirchhelle *et al* (2016)
*Arabidopsis thaliana* Columbia	UBQ10pro:EosFP‐DCP1	This paper
*Arabidopsis thaliana* Columbia	PIN2pro:PIN2‐GFP	Xu & Scheres (2005)
*Arabidopsis thaliana* Columbia	PIN7pro:PIN7‐GFP	Zhou *et al* (2010)
**Recombinant DNA**		
pICSL86900‐OD	Addgene	86178
pICSL13002	Addgene	50266
pICH47751	Addgene	48002
pICH41414	Addgene	50337
pGWB560	Nakagawa *et al* (2007)	
pG5 (RPS5apro:HF‐GW‐tagRFP)	This paper	
pIF1 (RPS5apro:HF‐mScarlet‐GW)	This paper	
pIF22 (RPS5apro:mNeon‐GW)	This paper	
Codon‐optimized TurboID	Arora *et al* (2020)	
35Spro:sGFP‐TurboID‐HF/WT	Arora *et al* (2020)	
35Spro:DCP1‐TurboID‐HF/WT	This paper	
35Spro:GFP‐DCP1/WT	Gutierrez‐Beltran *et al* (2015)	
RPS5apro:ARPC5‐tagRFP	This paper	
RPS5apro:mNeon‐ARPC5	This paper	
pUbiCAS9‐Red	Durr *et al* (2018)	
pSITE17 (nYFP‐GW)	Chakrabarty *et al* (2007)	
pSITE18 (cYFP‐GW)	Chakrabarty *et al* (2007)	
pSITE18 (nYFP‐GW)‐DCP1	This paper	
pSITE17 (cYFP‐GW)‐AT1G33050	This paper	
pSITE17 (cYFP‐GW)‐AT2G26920	This paper	
pSITE17 (cYFP‐GW)‐RH12	This paper	
pSITE17 (cYFP‐GW)‐FLXL1	This paper	
pSITE17 (cYFP‐GW)‐XRN3	This paper	
pG5‐ECT6	This paper	
pG5‐ECT4	This paper	
pG5‐VAP27‐1	This paper	
pG5‐AT2G26920	This paper	
pG5‐AT1G33050	This paper	
pG5‐AT5G53330	This paper	
pG5‐FLXL1	This paper	
pG5‐MLN51	This paper	
pG5‐EIN2	This paper	
**Antibodies**		
Mouse α‐DCP1	This paper	
Polyclonal mouse α‐PAT1	Roux *et al* (2015)	
Monoclonal α‐FLAG® M2‐Peroxidase (HRP)	Sigma‐Aldrich	A8592
Monoclonal mouse α‐FLAG® M2	Sigma‐Aldrich	F1804
Polyclonal Rabbit α‐Green Fluorescent Protein (GFP)	Millipore	AB10145
α‐streptavidin‐HRP	Sigma‐Aldrich	GERPN1231
Monoclonal Mouse α‐streptavidin	Sigma‐Aldrich	189730
Monoclonal Mouse α‐GFP antibody	Sigma‐Aldrich	SAB2702197
Monoclonal Mouse α‐Red Fluorescent Protein (RFP) (clone RF5R)	Agrisera	AS15 3028
Monoclonal Rat α‐tubulin (YL1/2)	Santa Cruz Biotechnology	sc‐53029
Alexa Fluor® 488 phalloidin	ThermoFisher Scientific	A12379
Rhodamine Red™‐X (RRX) 570 AffiniPure Donkey α‐Mouse IgG (H + L)	Jackson ImmunoResearch	715‐295‐151
Rhodamine (TRITC) AffiniPure Donkey α‐Rat IgG (H+L)	Jackson ImmunoResearch	712‐025‐153
IRDye® 680LT Goat α‐Mouse IgG (H + L)	LI‐COR	925‐68020
IRDye® 800CW Goat α‐Rat IgG (H + L)	LI‐COR	925‐32219
Amersham ECL Rabbit IgG, HRP‐linked whole Ab (from donkey)	Amersham	NA934
Amersham ECL Mouse IgG, HRP‐linked whole Ab (from sheep)	Amersham	NA931
Polyclonal rabbit α‐His		
**Oligonucleotides and sequence‐based reagents**		
Primers used for cloning	This study	Appendix Table S1
Primers used for verification of T‐DNA mutants	This study	Appendix Table S2
Primers used for generating CRISPR mutants	This study	Appendix Table S3
**Chemicals, enzymes, and other reagents**		
α‐FLAG® M2 Magnetic Beads	Sigma‐Aldrich	M8823
Dynabeads® M‐280 Streptavidin	Thermo Fisher Scientific	11205D
PD‐10 Desalting Columns	Cytiva	17085101
Glutathione Sepharose 4B	Cytiva	17075601
IgG sepharose	Cytiva	170969‐01
Ni‐NTA Agarose	Qiagen Ab	30210
HisPur™ Cobalt Resin	ThermoFisher Scientific	89964
Duolink® In Situ PLA® Probe α‐Rabbit PLUS Affinity purified Donkey anti‐Rabbit IgG (H + L)	Sigma‐Aldrich	DUO92002‐100RXN
Duolink® In Situ PLA® Probe α‐Mouse MINUS Affinity purified Donkey anti‐Mouse IgG (H + L)	Sigma‐Aldrich	DUO92004‐100RXN
Duolink® In Situ PLA® Probe α‐Goat PLUS Affinity purified Donkey anti‐Goat IgG (H + L)	Sigma‐Aldrich	DUO92003‐100RXN
Duolink® In Situ Detection Reagents Red	Sigma‐Aldrich	DUO92008‐100RXN
Duolink® In Situ Detection Reagents FarRed	Sigma‐Aldrich	DUO92013‐100RXN
Duolink® In Situ Probemaker PLUS	Sigma‐Aldrich	DUO92009‐1KT
Duolink® In Situ Wash Buffers, Fluorescence	Sigma‐Aldrich	DUO82049
Gentamycin sulfate	Saveen Werner AB (Duchefa)	G0124.0005
Ampicillin	Saveen Werner AB (Duchefa)	A0104.0010
Spectinomycin	Saveen Werner AB (Duchefa)	S0188.0005
Rifampicin	Saveen Werner AB (Duchefa)	R0146.0005
Kanamycin	Saveen Werner AB (Duchefa)	K0126.0005
Biotin	Sigma‐Aldrich	B4501
Amiprofos methyl (APM)	Sigma‐Aldrich	3992
Latrunculin B (Lat B)	Sigma‐Aldrich	L5288
Cytochalasin D (Cyt D)	Santa Cruz Biotechnology	sc‐201442
Dithiothreitol (DTT)	ThermoFisher Scientific	R0861
Protease inhibitors cocktail	Sigma‐Aldrich	P9599
PhosSTOP	Roche	4906845001
Propidium Iodide	Sigma‐Aldrich	P4170
IGEPAL CA‐630	Sigma‐Aldrich	I8896
Isopropyl‐b‐D‐1thiogalactopyranoside (IPTG)	Sigma‐Aldrich	I6758
Imidazole	Sigma‐Aldrich	56748
TRIzol® Reagent	ThermoFisher Scientific	15596018
30% Acrylamide/Bis Solution, 29:1	Biorad	161‐0156
4× Laemmli Sample Buffer	Biorad	1610747
RiboLock Rnase Inhibitor	ThermoFisher Scientific	EO0381
DNase I, RNase‐free	ThermoFisher Scientific	EN0521
Mesh	SefarNitex	03‐25/19
VectaShield	Vector Laboratories	H‐1200
FM4‐64	ThermoFisher Scientific	T13320
Cycloheximide (CHX)	Sigma‐Aldrich	C7698
Isoxaben	Sigma‐Aldrich	36138
DynaMag™‐2 Magnet	ThermoFisher Scientific	112321D
Phenol: chloroform: iso‐amyl alcohol (25:24:1)	VWR	136112‐00‐0
Acetosyringone	Sigma‐Aldrich	D134406
PageRuler™ Plus Prestained Protein Ladder, 10 to 250 kDa	ThermoFisher Scientific	26619
Eppendorf 1.5 ml Protein LoBind Microcentrifuge tubes	VWR	525‐0133
Bovine serum albumin (BSA)	Sigma‐Aldrich	A7030
Deionized Formamide	Sigma‐Aldrich	S4117
m‐maleimidobenzoyl‐N‐hydroxysuccinimide ester (MBS)	ThermoFisher Scientific	22311
2‐fluoro‐N‐[2‐(2‐methyl‐1H‐indol‐3‐yl)ethyl]‐benzamide (CK‐666)	Sigma‐Aldrich	SML0006
Phusion™ High‐Fidelity DNA Polymerase & dNTP Mix	ThermoFisher Scientific	F530N
SuperScript III First‐Strand Synthesis System for RT‐PCR	ThermoFisher Scientific	18080‐051
Maxima SYBR Green/Flouorescein qPCR Master Mix	ThermoFisher Scientific	K0243
Gateway® BP Clonase™ II Enzyme Mix	ThermoFisher Scientific	11789‐020
Gateway® LR Clonase® II Enzyme mix	ThermoFisher Scientific	11791020
Colloidal blue staining	ThermoFisher Scientific	LC6025
ECL Prime Western Blotting Detection Reagent	Cytiva	GERPN2232
**Software**		
Cytoscape v3.4.0	http://www.cytoscape.org	Shannon *et al* (2003)
FIJI	https://fiji.sc/	Schindelin *et al* (2012)
RootTrace	http://www.plant‐image‐analysis.org/software/roottrace	French *et al* (2008)
JACoP	https://imagej.nih.gov/ij/plugins/track/jacop.html	Bolte and Cordelieres (2006)
Graphpad Prism v9	https://www.graphpad.com/scientific‐software/prism/	Commercial
Adobe Photoshop 2023	https://www.adobe.com	Commercial
Search Tool for the Retrieval of Interacting Genes/Proteins	https://string‐db.org	Freeware
R v4.4.2	https://www.r‐project.org	Freeware
**Other**		
In Fusion HD Cloning Kit	Clonetech/Takara	638909
SuperSignal Western Blot Enhancer	ThermoFisher Scientific	46641
SuperSignal™ West Femto Maximum Sensitivity Substrate	ThermoFisher Scientific	34094
SP8 LIGHTINING MODE CONFOCAL MICROSCOPE	https://www.leica‐microsystems.com	Commercial
Phire Plant Direct PCR Master Mix	ThermoFisher Scientific	F160S
pENTR™/D‐TOPO™ Cloning Kit	Thermo Fisher Scientific	K240020
GeneJET Plasmid Miniprep Kit	ThermoFisher Scientific	K0503
GeneJET Gel Extraction Kit	ThermoFisher Scientific	K0692
Thermo Scientific™ Orbitrap Fusion™ Lumos™ Tribrid™ Mass Spectrometer	https://www.thermofisher.com/order/catalog/product/IQLAAEGAAPFADBMBHQ	Commercial

### Methods and Protocols

#### Plant materials

All the plant lines used in this study were in the *Arabidopsis* Columbia‐0 (Col‐0) accession except the ones indicated below. Primers used for genotyping and cloning are described in Appendix Tables. Except for the Transfer (T)‐DNA insertion mutants obtained from the NASC (The Nottingham Arabidopsis Stock Centre) as indicated in the Reagents and Tools table above, the following mutants and transgenic lines used in this study were described previously: *dcp1‐1* (Xu *et al*, 2006), *dcp1‐3* (Martinez de Alba *et al*, 2015), *dcp2‐1* (Chantarachot *et al*, 2020), *dcp5‐1* (Xu & Chua, 2009), *pat1‐1* (Roux *et al*, 2015), *xrn4‐5* (Souret *et al*, 2004), *brk1* (Dyachok *et al*, 2008), *scar1234* (Dyachok *et al*, 2008), *arpc5* (Pratap Sahi *et al*, 2017), *35S:GFP‐DCP1* (Gutierrez‐Beltran *et al*, 2015), *35Spro:DCP2‐YFP* (Jang *et al*, 2019), *dcp5‐1 DCP5pro:DCP5‐3HA* (Xu & Chua, 2009), *RH12pro:RH12‐GFP* (Jang *et al*, 2019), *DCP1pro:DCP1‐GFP* (Yu *et al*, 2019), *DCP1pro:DCP1* ^*S237A*^ *‐GFP* and *DCP1pro:DCP1* ^*S237D*^ *‐GFP* (Yu *et al*, 2019), *SOK3pro:SOK3‐YFP* (van Dop *et al*, 2020), *SCAR2pro:SCAR2‐mCherry* (preprint: Chin *et al*, 2020), *BRK1pro:BRK1‐mRuby* (preprint: Chin *et al*, 2020), *BRK1pro:BRK1‐YFP* (Dyachok *et al*, 2008), *UBQ10pro:LifeAct‐mCherry* (preprint: Chin *et al*, 2020), *SPIpro:SPI‐Ypet* (preprint: Chin *et al*, 2020), *PIN2pro:PIN2‐GFP* (Xu & Scheres, 2005), *PIN7pro:PIN7‐GFP* (Zhou *et al*, 2010), *DEX˃RAB‐A2c* ^*DN*^ and *RAB‐A5cpro:RAB‐A5c* (Kirchhelle *et al*, 2016). In all experiments, plants from T1/F1 (co‐localization experiments), T2/F2, or T3 (for physiological experiments) generations were used.

#### Construction of the sok1 sok3 CRISPR deletion mutant

EC1‐driven *SOK1* deletion constructs were made as follows: single guide RNAs (sgRNAs) were synthesized with respective overhangs. The two complementary oligos were annealed and inserted into pEN‐2xChimera using BpiI and BsmBI restriction enzymes. The sgRNAs were then transferred into pUbiCAS9‐Red (for protoplasts) or pEciCAS9‐Red (for stable transformation in *A. thaliana*) by Gateway^®^ single‐site LR recombination‐mediated cloning. The efficiency of the sgRNAs was tested in protoplasts: Arabidopsis mesophyll protoplasts for transient expression of the CRISPR/Cas9 construct (pUbiCAS9‐Red) were prepared as described previously with minor modifications (Yoo *et al*, 2007). Approximately, 80,000 protoplasts were transformed with 16 μg of plasmid (pUbiCAS9‐Red) and incubated for 48 h at 22°C under long photoperiod conditions (150 μmol/m^2^/s and 16‐h‐light/8‐h‐dark cycles). Genomic DNA was isolated, and concentrations were adjusted before performing semi‐quantitative PCR using oligonucleotides flanking the region targeted for deletion (Liu *et al*, 2017; Labun *et al*, 2019; Ursache *et al*, 2021). To create the *SOSEKI3* (AT2G28150) deletion mutant, we used a previously described multiplexed editing approach (Ursache *et al*, 2021). We designed sgRNAs using the CRISPR‐P 2.0 (http://crispr.hzau.edu.cn/CRISPR2; Liu *et al*, 2017) and CHOP‐CHOP (https://chopchop.cbu.uib.no/; Labun *et al*, 2019) web tools. To delete the complete coding sequence of the gene, we targeted two sites near the start codon of *SOK3*, and one site downstream of the stop codon, along with equivalent sgRNAs for *SOK2* (AT5G10150). Following the published protocol, we cloned all sgRNAs into a single pRU292 destination vector. This vector allows for zCas9i expression under the *UBi4.2* promoter, and expression of the sgRNAs and has a FastGreen selection marker. sgRNAs and oligonucleotide sequences used for cloning are shown in Source Table 1. The resulting binary vector was transformed into the *sok1* mutant background using the floral dipping method. We selected green fluorescing seeds and screened the resulting seedlings for large deletions in *SOK3* and the absence of large deletions in *SOK2* using PCR. We validated the mutations in *SOK3* by direct gene‐specific sequencing and crossed out the transgene by selecting non‐fluorescent seeds in the T2 generation. We confirmed that *SOK2* was not deleted in this line, but there were small deletions in this gene. In the T3 generation, we selected double homozygous *sok1 sok3* plants.

#### Plant growth conditions

Arabidopsis seedlings were sterilized and germinated on half‐strength Murashige and Skoog (MS) agar medium under long‐day conditions (16 h light/8 h dark). In all experiments involving the use of mutants or pharmacological treatments, the medium was supplemented with 1% (w/v) sucrose or as otherwise specified. Arabidopsis plants for crosses, phenotyping of the above‐ground part, and seed collection were grown on soil in a plant chamber at 22°C/19°C, 14‐h‐light/10‐h‐dark or 16‐h‐light/8‐h‐dark cycles, and light intensity 150 μE/m^2^/s. Arabidopsis and *Nicotiana benthamiana* plants were grown in Aralab or Percival cabinets at 22°C, 16‐h‐light/8‐h‐dark cycles, and a light intensity of 150 μE/m^2^/s.

#### Phenotypic analysis and drug treatments

For quantification of phenotypes, seedlings were surface sterilized and grown on half‐strength MS medium plates with 1% (w/v) sucrose. For a given genotype, differential contrast interference (DIC) images were captured on a Leica DM2500, Leica DM6B, or Leica DM6000. To define root length, images were captured from the plates using a Leica DM6000 with a motorized stage and computationally compiled together. Root length or size was determined using Image J/Fiji by comparing the measurement with the WT or mock conditions (National Institute of Health). The stock solutions of 50 mM biotin, 2 mM FM4‐64, 2 mM dexamethasone, 10 mM cytochalasin D (Cyto D), 10 mM Amiprofos methyl (APM), 1 mM latrunculin B, 50 mM brefeldin A (BFA), 200 mM CK666 and 50 mM cycloheximide (CHX) were dissolved in dimethyl sulfoxide (DMSO), while 40 mM isoxaben was dissolved in ethanol. 1.0 mg/ml Propidium iodide (PI) was in water. These inhibitors or drugs were diluted in half‐strength MS medium with corresponding concentration and duration, and the final DMSO concentration was ≤ 0.1% (v/v) in all experimental analyses. Vertically grown 4‐ to 5‐day‐old Arabidopsis seedlings were incubated in a half‐strength liquid MS medium containing the corresponding drugs for each specific time course treatment as indicated.

#### Bacterial strains, cloning, and constructs

Electrocompetent Agrobacterium (*Agrobacterium tumefaciens*) strain C58C1 Rif^R^ (pMP90) or GV3101 Rif^R^ (i.e., a cured nopaline strain commonly used for infiltration) was used for electroporation and *Nicotiana benthamiana* infiltration. The Goldengate‐compatible TurboID vector (*35Spro:sGFP‐TurboID‐HF*) was previously described (Arora *et al*, 2020). Cloning was done according to standard Goldengate cloning procedures. In brief, TurboID was synthesized with BsaI overhangs using the codon optimization tool of Integrated DNA Technologies for codon‐optimized expression in Arabidopsis (Eurofins). The coding sequence of *DCP1* was PCR amplified using Phusion™ High‐Fidelity DNA Polymerase & dNTP Mix (Thermo Fisher Scientific, F530N) using the GFP‐DCP1 vector as a template. The 35S promoter carrying level 0 vector (pICSL13002) and all PCR parts with BsaI overhands were ligated to the Level1/2 vectors pICSL86900 and pICSL86922. Other constructs of DCP1 were generated by Gateway cloning with pENTR‐DCP1 into different destination vectors which have different tags in N or C termini as indicated in the text or legends. The coding sequence of *ARPC5* was PCR amplified using Phusion™ High‐Fidelity DNA Polymerase & dNTP Mix (Thermo Fisher Scientific, F530N) using the cDNA from 7‐day‐old seedling of WT plants with pENTR™/D‐TOPO™ Cloning Kit (Thermo Fisher Scientific, K240020). For cloning, the bacterial strain NEB10 (New England Biolabs #C3019H) or NEB stable (New England Biolabs C3040H; CRISPR constructs) was used. For BiFC and colocalization constructs, At1g33050, At2g26920, *ECT4*, *ECT6*, *MLN51*, *FLXL1*, *EIN2*, *VAP27‐1*, and At5g53330 were generated by Gateway cloning. pENTR vectors were generated via BP reaction with pDONR221 (Invitrogen) and PCR product amplicons from RT‐PCR using cDNA from 1‐week‐old seedlings. As destination vectors, pSITE17 (nYFP‐GW), pSITE18 (cYFP‐GW), and pG5 vector (custom‐made Gateway vector with tagRFP) were used.

#### DCP1 antibody production

The *DCP1* cDNA in pGAT4 (hexahistidine‐tagged vector) was transformed in BL21 (DE3) Rosetta or BL21 (DE3) Rosetta II *Escherichia coli* cells. Bacterial cultures were grown in 800 ml of Luria Bertani (LB) medium supplemented with 100 mg/l of ampicillin and 25 mg/l of chloramphenicol. Protein production was induced at OD_600_ = 0.5 with 0.05 to 1 mM isopropyl β‐D‐1‐thiogalactopyranoside (IPTG). After 3 h, the cells were harvested by centrifugation at 2,500 *g* for 20 min at room temperature and frozen overnight at −80°C. Preparation of hexahistidine‐tagged recombinant DCP1 was performed according to the manufacturer's instructions in Tris‐buffer (Qiagen, 30210). The abundance of proteins was estimated by coomassie brilliant blue (CBB) staining by SDS‐PAGE or on immunoblots using α‐his. The DCP1 protein was dialyzed overnight in assay buffer (2 l) and was used to immunize four mice. The antibodies were further purified by solid‐phase absorption using columns with DCP1. Pre‐immune sera were also collected and used as an additional negative control, producing no signal.

#### Immunoblotting

Infiltrated *N. benthamiana* leaves or Arabidopsis leaves and seedlings were harvested, and their proteins were extracted. The tissue samples were flash‐frozen in liquid Ν_2_ and kept at −80°C until further processing. The samples were crushed using a liquid Ν_2_‐cooled mortar and pestle, and the crushed material was transferred to a 1.5‐ml or 15‐ml tube. Extraction buffer (EB; 50 mM Tris–HCl pH 7.5, 150 mM NaCl, 10% [v/v] glycerol, 2 mM ethylenediamine tetra acetic acid [EDTA], 5 mM dithiothreitol [DTT], 1 mM phenylmethylsulfonyl fluoride [PMSF], Protease Inhibitor Cocktail [Sigma‐Aldrich, P9599] and 0.5% [v/v] IGEPAL CA‐630 [Sigma‐Aldrich]) was added according to the plant material used. The lysates were pre‐cleared by centrifugation at 16,000 *g* at 4°C for 15 min, and the supernatant was transferred to a new 1.5‐ml tube. This step was repeated two times and the protein concentration was determined by the RC DC Protein Assay Kit II (Bio‐Rad, 5000122). Two times Laemmli buffer was added, and equivalent amounts of protein (~ 30 μg) were separated by Sodium dodecyl‐sulfate polyacrylamide gel electrophoresis (SDS‐PAGE; 1.0 mm thick 4–12% [w/v] gradient polyacrylamide Criterion Bio‐Rad) in 3‐(N‐Morpholino) propane sulfonic acid (MOPS) buffer (Bio‐Rad) at 150 V. Subsequently, proteins were transferred onto a polyvinylidene fluoride (PVDF; Bio‐Rad) membrane with 0.22‐μm pore size. The membrane was blocked with 3% (w/v) BSA fraction V (Thermo Fisher Scientific) in phosphate buffered saline‐Tween 20 (PBS‐T) for 1 h at room temperature (RT), followed by incubation with horseradish peroxidase (HRP)‐conjugated primary antibody at RT for 2 h (or primary antibody at RT for 2 h and corresponding secondary antibody at RT for 2 h). The following antibodies were used: streptavidin‐HRP (Sigma‐Aldrich; 1:25,000, N100), mouse α‐FLAG‐HRP (Sigma‐Aldrich, A8592, 1:2,000), rat α‐tubulin (Santa Cruz Biotechnology, 1:1,000), rabbit α‐GFP (Millipore, AB10145, 1:10,000), mouse α‐RFP (Agrisera, AS15 3028, 1:5,000), α‐mouse (Amersham ECL Mouse IgG, HRP‐linked whole Ab [from sheep], NA931, 1:10,000), α‐rabbit (Amersham ECL Rabbit IgG, HRP‐linked whole Ab [from donkey], NA934, 1:10,000), α‐rat (IRDye® 800 CW Goat α‐Rat IgG [H + L], LI‐COR, 925‐32219, 1:10,000), and α‐rabbit (IRDye ® 800 CW Goat α‐Rabbit IgG, LI‐COR, 926‐3221, 1:10,000). Chemiluminescence was detected with the ECL Prime Western Blotting Detection Reagent (Cytiva, GERPN2232) and SuperSignal™ West Femto Maximum Sensitivity Substrate (Thermo Fisher Scientific, 34094). The bands were visualized using an Odyssey infrared imaging system (LI‐COR).

#### APEAL approach details

After 24 h treatment (syringe infiltration) with 50 μM biotin (diluted in 10 mM MgCl_2_, 10 mM MES pH 5.7), 2.5 ml pulverized Arabidopsis leaves or seedlings (~ 0.5 g FW) were extracted in 5 ml EB (50 mM Tris–HCl pH 7.5, 150 mM NaCl, 10% [v/v] glycerol, 0.5 mM EDTA, 1 mM DTT, 0.5% [v/v] IGEPAL CA‐630 [Sigma‐Aldrich] and Protease inhibitor Cocktail [1:100 dilution, Sigma‐Aldrich, P9599]). Extracts were incubated on a shaker at 4°C for 10 min and then centrifuged at 4°C, 13,000 *g* for 30 min. Five milliliters of clarified supernatants were incubated with 100 μl magnetic FLAG beads (Sigma‐Aldrich, A8592) at 4°C for 2 h with gentle rotation, then the FLAG beads were precipitated with DynaMag™‐2 Magnet (Thermo Fisher Scientific, 112321D) and washed four times with 1 ml EB (5 min each time). The supernatants (“flow‐through1”) were filtered through PD‐10 columns (Cytiva, 17085101), then the biotin‐depleted “flow‐though2” was incubated with 100 μl Dynabeads™ M‐280 Streptavidin (Thermo Fisher Scientific, 11205D) at 4°C for 2 h with gentle rotation. The FLAG beads and Dynabeads were washed five times with 1 ml EB and eight times with 1 ml 50 mM NH_4_HCO_3_ (5 min each time). The beads were then collected on a magnetic rack and subjected to on‐beads digestion followed by mass spectrometry. For immunoblot analysis, the proteins were eluted from the FLAG beads with 60 μl 2× Laemmli buffer (Bio‐Rad, 1610747) supplemented with 10 mM DTT and incubated at 95°C for 10 min. The proteins were eluted from the Dynabeads with 60 μl 2× Laemmli buffer supplemented with 5 mM biotin, 2% (w/v) SDS, and 10 mM DTT and incubated at 95°C for 20 min.

#### On‐beads digestion

After immunoprecipitation and extensive washing with 25 mM NH_4_HCO_3_, 0.1 μg trypsin in 10 μl of 2 mM CaCl_2_, 10% (v/v) acetonitrile, and 25 mM NH_4_HCO_3_ was added to beads and incubated at 37°C overnight. Fresh trypsin (0.1 μg) in 10 μl 25 mM NH_4_HCO_3_ was added to beads and incubated for another 4 h. The digested supernatant was transferred into a clean centrifuge tube, and acetonitrile was evaporated under a vacuum. The samples were then acidified and desalted using a C18 stage tip before being analyzed by nano‐liquid chromatography–tandem mass spectrometry.

#### Liquid chromatography–tandem mass spectrometry

Samples were analyzed by LC–MS using a Nano LC–MS/MS apparatus (Dionex Ultimate 3000 RLSCnano System) interfaced with an Eclipse Tribrid mass spectrometer (Thermo Fisher Scientific). Samples were loaded onto a fused silica trap column (Acclaim PepMap 100, 75 μm × 2 cm; Thermo Fisher Scientific). After washing for 5 min at 5 μl/min with 0.1% (v/v) trifluoroacetic acid (TFA), the trap column was brought in‐line with an analytical column (Nanoease MZ peptide BEH C18, 130 A, 1.7 μm, 75 μm × 250 mm, Waters) for LC–MS/MS. Peptides were fractionated at 300 nl/min using a segmented linear gradient 4–15% B in 30 min (where A: 0.2% formic acid, and B: 0.16% formic acid, 80% acetonitrile), 15–25% B in 40 min, 25–50% B in 44 min, and 50–90% B in 11 min. Solution B was then returned to 4% for 5 min for the next run. The scan sequence began with an MS1 spectrum (Orbitrap analysis, resolution 120,000, scan range from *m/z* 350–1,600, automatic gain control (AGC) target 1E6, maximum injection time 100 ms). The top S (3 s) and dynamic exclusion of 60 s were used for the selection of parent ions for MS/MS. Parent masses were isolated in the quadrupole with an isolation window of 1.4 *m/z*, automatic gain control (AGC) target 1E5, and fragmented with higher‐energy collisional dissociation with a normalized collision energy of 30%. The fragments were scanned in Orbitrap with a resolution of 30,000. The MS/MS scan range was determined by the charge state of the parent ion, but the lower limit was set at 100 amu.

#### Database search

LC–MS/MS data were analyzed with Maxquant (version 1.6.10.43) with the Andromeda search engine. The type of LC–MS run was set to 1 (label‐free). LC–MS data were searched against The Arabidopsis Information Resource (TAIR) plus a common contaminant database. Protease was set as trypsin, which allowed two miscuts. N‐terminal acetylation and oxidation at methionine were set as variable modifications. Maximum two variable modification was allowed. Protein and peptide false discovery rate (FDR) were set to 1%. Reverse hit and common contaminants, as well as proteins identified only by modified sites, were removed. GO term analysis was performed using a combination of the Panther database and BioConductor package in R (Bonnot *et al*. 2019).

#### Visualization of networks and analyses

Cytoscape 3.5.1 was used. Tab‐delimited files containing the input data were uploaded. Unless otherwise indicated, the default layout was an edge‐weighted spring‐embedded layout, with NormSpec used as edge weight. Nodes were manually re‐arranged from this layout to increase visibility and highlight specific proximity interactions. The layout was exported as a PDF and eventually converted to a. TIFF file with Lempel–Ziv–Welch (common name LZW) compression.

#### Agrobacterium‐mediated transient transformation of *Nicotiana benthamiana*

*N. benthamiana* plants were grown under normal light and dark regimes at 25°C and 70% relative humidity. Three‐ to four‐week‐old *N. benthamiana* plants were watered from the bottom ~ 2 h before infiltration. Transformed Agrobacterium strain C58C1 Rif^R^ (pMP90) or GV3101 Rif^R^ harboring the constructs of interest were used to infiltrate *N. benthamiana* leaves and for transient expression of binary constructs by Agrobacterium‐mediated transient infiltration of lower epidermal leaf cells. Transformed Agrobacterium colonies were grown for ~ 20 h in a shaking incubator (200 rpm) at 28°C in 5 ml of yeast extract broth (YEB) medium (5 g/l beef extract, 1 g/l yeast extract, 5 g/l peptone, 0.5 g/l MgCl_2_, and 15 g/l bacterial agar), supplemented with appropriate antibiotics (i.e., 100 g/l spectinomycin). After incubation, the bacterial culture was transferred to 15‐ml Falcon tubes and centrifuged (10 min, 5,000 *g*). The pellets were washed with 5 ml of infiltration buffer (10 mM MgCl_2_, 10 mM MES pH 5.7), and the final pellet was resuspended in infiltration buffer supplemented with 100 μM acetosyringone from a stock diluted in dimethyl formamide. The bacterial suspension was diluted with infiltration buffer to adjust the inoculum cell density to a final OD_600_ value of 0.2–1.0. The inoculum was incubated for 2 h at room temperature before infiltration into *N. benthamiana* leaves by gentle pressure infiltration of the lower epidermis of leaves (fourth and older true leaves were used, and about 4/5‐1/1 of their full size) with a 1‐ml hypodermic syringe without a needle.

#### Bimolecular fluorescence complementation (BiFC)

The BiFC assay was done in *N. benthamiana* plants. Excitation wavelengths and emission filters were 514 nm/band‐pass 530–550 nm for YFP and 488 nm/band‐pass 650–710 nm for chloroplast auto‐fluorescence. The objective used was a HC PL APO 40×/1,30 oil CS2 with NA = 1.3 (Leica SP8 confocal system).

#### TIRFM imaging and tracking analyses

TIRF microscopy images were acquired using MetaMorph software on an Olympus IX‐81 microscope. A DV2 image splitter (MAG Biosystems) was used to separate GFP and RFP emission signals. Time‐lapse movies were obtained at 100‐ms intervals. For MSD analysis, 30‐s‐long movies with 100‐ms intervals and 200‐ms exposure were used. Particle tracking was limited to the amount of time that the plasma membrane remained at the focal plane; the median track length was 2,000 frames, corresponding to 3.5 s of imaging. The tracking of particles was performed with the Mosaic suite of Fiji or NanoTrackJ/TrackMate, using the following parameters: radius 3 of fluorescence intensity, a link range of 1, cutoff of 0.1%, and a maximum displacement of 8 px, assuming Brownian dynamics.

#### Quantification of fluorescent intensity, FRAP, and FRET

To create the most comparable lines to measure the fluorescence intensity of reporters in multiple mutant backgrounds, we crossed homozygous mutant plants carrying the marker with either a wild‐type plant (to yield progeny heterozygous for the recessive mutant alleles and the reporter) or crossed to a mutant only plant (to yield progeny homozygous for the recessive mutant alleles and heterozygous for the reporter). Fluorescence was measured as a mean integrated density in regions of interest (ROIs) with the subtraction of the background (a proximal region that was unbleached and had less signal intensity than the signal of the ROI region). FRAP mode of Zeiss 780 ZEN software was set up for the acquisition of 3 pre‐bleach images, 1 bleach scan, and 96 post‐bleach scans (or more). Bleaching was performed using the 488‐, 514‐, and 561‐nm laser lines at 100% transmittance and 20–40 iterations depending on the region and the axial resolution (iterations increased in deeper tissues to compensate for the increased light scattering). In FRAP the width of the bleached ROI was set to 2–10 μm. Pre‐ and post‐bleach scans were at minimum possible laser power (0.8% transmittance) for 458 nm or 514 nm (4.7%) and 5% for 561 nm; 512 × 512 8‐bit pixel format; pinhole of 181 μm (> 2 Airy units) and zoom factor of 2.0. The background values were subtracted from the fluorescence recovery values, and the resulting values were normalized by the first post‐bleach time point and divided by the maximum fluorescent time‐point set maximum intensity as 1. The objective used was a plan‐apochromat 40× with NA = 1.2 M27 (Zeiss). The following settings were used for photobleaching DCP1: 10–20 iterations for DCP1‐GFP; 10 to 60 s per frame; 100% transmittance with the 458‐ to 514‐nm laser lines of an argon laser. Pre‐ and post‐bleach scans were at minimum possible laser power (1.4 to 20% transmittance) for the 488‐nm and 0% for all other laser lines, 512 × 512‐pixel format, and zoom factor of 5.1. The fluorescence intensity recovery values were determined, the background values were subtracted from the fluorescence recovery values, and the resulting values were normalized against the first post‐bleach time point. SE‐FRET analyses were conducted using the method described previously (preprint: Liu *et al*, 2022) and photoacceptor FRET was conducted with the method as described (Karpova *et al*, 2003). Image analyses and intensity measurements (Integrated Density) were done using Fiji v. 1.52 software (rsb.info.nih.gov/ij). For relative values, the intensities were normalized against the background signal intensity in raw images/micrographs. The calculations were done with arbitrary units (denoted as AUs). The dwell time rate of tagged proteins in FRAP experiments was calculated by the single exponential fit (Moschou *et al*, 2016). Colocalization was analyzed using Pearson statistics (Spearman or Manders analyses produced similar results or trends, with Fiji, coloc2 tool; French *et al*, 2008). Images were prepared in Adobe Photoshop version 2023. Time series movies were compressed, corrected, and exported as .avi extension files. The nonspecific fluorescence decay was corrected using Fiji and default options using the bleaching correction tool. Videos were digitally enhanced with Fiji‐implemented filters, correcting noise using the Gaussian blur option and pixel width set to 1.0.

#### Immunocytochemistry, PLA, and imaging

Immunocytochemistry was done as described previously (Moschou *et al*, 2016). The primary antibody used was rabbit α‐PAT1 (diluted 1:500; Roux *et al*, 2015), rat α‐tubulin YL1/2 (1:200; Santa Cruz Biotechnology), mouse α‐DCP1 (diluted 1:100; prepared herein), and α‐HA (diluted 1:300; Sigma‐Aldrich). In brief, specimens were washed three times for 90 min in PBS‐T and incubated overnight with anti‐rabbit fluorescein isothiocyanate‐conjugated (FITC) secondary antibody (Jackson ImmunoResearch, 711‐095‐152) diluted 1:200–250, DyLight™ 549 AffiniPure Fab Fragment Rabbit Anti‐Mouse IgG (H + L; Jackson ImmunoResearch, 315‐507‐003), Rhodamine Red™‐X (RRX) AffiniPure Donkey Anti‐Rabbit IgG (H + L; Jackson ImmunoResearch, 711‐295‐152), Tetramethylrhodamine (TRITC) Donkey Anti‐Rabbit IgG (H + L; Jackson ImmunoResearch, 711‐026‐152), Alexa Fluor® 647 AffiniPure Donkey Anti‐Mouse IgG (H + L; Jackson ImmunoResearch, 715‐605‐151) diluted 1:200–250. After washing in PBS‐T and incubation with or without DAPI (1 μg/ml), specimens were mounted in Vectashield (Vector Laboratories) medium and observed within 48 h. Root tips were imaged using a Zeiss 780 confocal laser scanning microscope. The objective used was a plan‐Apochromat 63×/1.4 oil immersion M27 (Zeiss) or an HC PL APO 40×/1.3 oil CS2 with NA = 1.3 (Leica SP8 confocal system).

Immunostaining of actin was done as described previously with minor modifications (Dyachok *et al*, 2008). Roots of 5‐day‐old Arabidopsis seedlings were fixed for 1 h in actin‐fixation buffer (50 mM PIPES, pH 7.2, with 20 mM EGTA and 20 mM MgSO_4_ containing 2% [w/v] paraformaldehyde, 0.1% [v/v] Triton X‐100 and 400 mM maleimidobenzoyl‐N‐hydroxy succinimide ester [Thermo Fisher Scientific, 22311]); the cell wall was digested for 30 min (in 0.2% [w/v] driselase and 0.15% [w/v] macerozyme). Then samples were incubated for 30 min in blocking buffer containing 2% (w/v) BSA and then incubated with Alexa Fluor 488 phalloidin (1:400 dilution, Thermo Fisher Scientific, A12379) overnight at 4°C. After washing with PBST buffer three times, samples were mounted in Vectashield (Vector Laboratories, Burlingame, CA) and root tips were imaged using a Zeiss 780 confocal laser scanning microscope. The objective used was a plan‐Apochromat 63×/1.4 oil immersion M27 (Zeiss).

PLA immunolocalization was done as described previously (Alam, 2018; preprint: Liu *et al*, 2022). Primary antibody combinations diluted 1:200 for α‐GFP mouse (Sigma‐Aldrich, SAB2702197), 1:200 for α‐FLAG mouse (Sigma‐Aldrich, F1804), 1:200 for α‐RFP mouse (Agrisera, AS15 3028), and 1:200 for α‐GFP rabbit (Millipore, AB10145) were used for overnight incubation at 4°C. Roots were then washed with microtubules‐stabilizing buffer (MTSB: 50 mM PIPES, 5 mM EGTA, 2 mM MgSO_4_, 0.1% [v/v] Triton X‐100) and incubated at 37°C for 3 h either with α‐mouse plus and α‐rabbit minus for PLA assay (Sigma‐Aldrich, Duolink). PLA samples were then washed with MTSB and incubated for 3 h at 37°C with ligase solution. Roots were then washed 2× with buffer A (Sigma‐Aldrich, Duolink) and treated for 4 h at 37°C in a polymerase solution containing fluorescent nucleotides as described (Sigma‐Aldrich, Duolink). Samples were then washed 2× with buffer B (Sigma‐Aldrich, Duolink), with 1% (v/v) buffer B for another 5 min, and then the specimens were mounted in Vectashield (Vector Laboratories) medium.

#### Statistics

The numerical data used in this publication are provided in Source Data as raw .csv files with the corresponding headings. Statistical analyses were performed in GraphPad (https://graphpad.com/) or R studio (R‐project.org). Graphs were also generated by GraphPad Prism or R. PCC is calculated via Fiji, coloc2 tool. All statistical data show the mean ± s.d. (box plots) or the distribution of values (violin plots, kernel density) of at least three biologically independent experiments or samples, or as otherwise stated. Individual data points are on the plots. For violin plots, datasets were smoothed using heavy smoothing which gives a better idea of the overall distribution. In captions, *N* denotes biological replicates, and “*n*” technical replicates or population size. Each data set was tested whether it followed normal distribution when *N* ≥ 3 by using the Shapiro normality test. The significance threshold was set at *P* < 0.05 (significance claim), and the exact *P* values are shown in the graphs or as asterisks. Details of the statistical tests applied, including the choice of the statistical method, are indicated in the corresponding figure caption. In boxplots or violin plots, upper and lower box boundaries, or lines in the violin plots when visible, represent the first and third quantiles, respectively, horizontal lines mark the median and whiskers mark the highest and lowest values. For regression analyses, the confidence intervals were calculated and are also shown (95% confidence band) of the best‐fit line. For Wilcoxon, *P* values are two‐tailed, for Kruskal–Wallis *P* values are approximate, for Welch‐ANOVA or Welch–Forsythe in multiple comparisons, *P* values are adjusted. For Kolmogorov–Smirnoff, *P* values are approximate. To increase the robustness of analyses when the normality was marginal (judged by the *P*), sometimes more than one test was used as indicated in the caption (data analyses). For acceptor photobleaching to determine the FRET‐efficiency, FDR corrections were done through two‐stage step‐up method of Benjamini, Krieger, and Yekutieli. In one crucial experiment (*dcp1‐3*/SCAR2 localization), the researcher did not know the genotype, which was determined later (blind experiment). In all other cases, the experiments were not blind.

## Author contributions

**Chen Liu:** Data curation; formal analysis; funding acquisition; investigation; visualization; methodology; writing – review and editing. **Andriani Mentzelopoulou:** Data curation; software; formal analysis; investigation; visualization; writing – review and editing. **Amna Muhammad:** Investigation. **Andriy Volkov:** Resources; formal analysis; investigation; writing – review and editing. **Dolf Weijers:** Resources; funding acquisition. **Emilio Gutierrez‐Beltran:** Resources; formal analysis; funding acquisition. **Panagiotis N Moschou:** Conceptualization; data curation; formal analysis; supervision; funding acquisition; investigation; visualization; methodology; writing – original draft; project administration; writing – review and editing.

## Disclosure and competing interests statement

The authors declare that they have no conflict of interest.

## Supporting information



Appendix

Expanded View Figures PDF

Source Data for Expanded View and Appendix

PDF+

Source Data for Figure 1

Source Data for Figure 2

Source Data for Figure 3

Source Data for Figure 4

Source Data for Figure 6

Source Data for Figure 7

Source Data for Figure 8

Source Data for Figure 9

Source Data for Figure 10

## Data Availability

The data generated or analyzed during this study are included in this published article. Additional information and materials generated for and/or reported in this article are available from the corresponding author upon request. The mass spectrometry proteomics data from this publication have been deposited to the ProteomeXchange Consortium via the PRIDE https://www.ebi.ac.uk/pride/ partner repository (Perez‐Riverol *et al*, 2022) with the dataset identifier PXD037701 (https://www.ebi.ac.uk/pride/archive/projects/PXD037701).
