# Supplementary material for: An actin remodeling role for Arabidopsis processing bodies revealed by their proximity interactome
Source: EMBO J. 2023 Feb 6;42(9):e111885. doi: 10.15252/embj.2022111885 (PMC10152145; doi:10.15252/embj.2022111885)
Supplement: Supplementary file 1 — Appendix [file EMBJ-42-e111885-s009.pdf]

EMBOJ-2022-111885_Appendix.PDF

The "Proxitome" of Arabidopsis Processing Bodies Reveals a Membrane Interface Remodeling Actin

Chen Liu[1], Andriani Mentzelopoulou[2,3], Amna Muhammad[1,4], Andriy Volkov[5], Dolf Weijers[5], Emilio Gutierrez-Beltran[6,7], Panagiotis N. Moschou[1,2,3*]

[1]Department of Plant Biology, Uppsala BioCenter, Swedish University of Agricultural Sciences and Linnean Center for Plant Biology, Uppsala, 75007, Sweden
[2]Department of Biology, University of Crete, Heraklion, 71500, Greece
[3]Institute of Molecular Biology and Biotechnology, Foundation for Research and Technology-Hellas, Heraklion, 70013, Greece
[4]University Institute of Biochemistry and Biotechnology, PMAS-Arid Agriculture University Rawalpindi, 46300, Pakistan
[5]Laboratory of Biochemistry, Wageningen University and Research, Wageningen, 6700, the Netherlands
[6]Instituto de Bioquímica Vegetal y Fotosíntesis, Universidad de Sevilla and Consejo Superior de Investigaciones Científicas, Seville, 41004, Spain
[7]Departamento de Bioquímica Vegetal y Biología Molecular, Facultad de Biología,Universidad de Sevilla, Sevilla, 41012, Spain

***Corresponding Author:**

Panagiotis.moschou@slu.se ,

Panagiotis.moschou@imbb.forth.gr ,

Panagiotis.moschou@uoc.gr

Table of Contents

Appendix Tables *3*

Appendix Table S1. DNA oligonucleotides for cloning 3

Appendix Table S2. DNA oligonucleotides used for genotyping 4

Appendix Table S3. DNA oligonucleotides used for CRISPR 5

Appendix Figures *6*

Appendix Fig S1. Unlike DCP1, other core PB components localize at the PM only transiently.... 6

Appendix Fig S2. DCP1/DCP2 or DCP1/PAT1 spatiotemporal interaction by PLA and SE-FRET..... 8

Appendix Fig S3. DCP1 is not strongly accumulating in BFA bodies. 10

Appendix Fig S4. DCP1 is likely not recruited to the cell edge by Rab-A5c or SOK3. 11

Appendix Fig S5. The colocalization and interaction of DCP1 with ARPC5. 13

Appendix Fig S6. Cytochalasin D and Latrunculin B effects on DCP1 localization. 15

Appendix Fig S7. A microtubule-depleted zone confines DCP1 at the edge/vertex. 16

Appendix Fig S8. DCP1 can affect ARPC5 localization and spatial restriction at the edge. 17

Appendix Fig S9. Effect of isoxaben treatment on isotropic expansion of phosphovariant DCP1 lines and differential expansion of DCP1 phosphovariants, PBs core mutants or SCAR-WAVE mutants. 18

Appendix text *19*

Subnetwork 1: RNA metabolism 19

Subnetwork 2: Defense Regulation and Metabolism 19

Subnetwork 3: Membrane remodeling/trafficking 19

Subnetwork 4: Novel Interactors: Actin polymerization and cytoskeleton remodeling 20

References *20*

Appendix Tables

Name	Sequence 5'-3'
Appendix Table S1. DNA oligonucleotides for cloning	
DCP1 F	GTGGTCTCAAATGTCTCAAAACGGGAAGA
DCP1 R	GTGGTCTCACGAACCTTGTTGAAGTGCAT
6*HIS-3*FLAG F	GTGGTCTCACCATGGGTTCCGGAAGAGGATCGCA
6*HIS-3*FLAG R	GTGGTCTCACATTCCCTTGTCATCGTCATCCTTG
35Spro F	GTGGTCTCAGGAGGGAGGAATTCCAATCCCACA
35Spro R	GTGGTCTCACATTGCGTGTCCTCTCCAAATGAA
sGFP F	GTGGTCTCAATGGGCCCCGACATCGT
sGFP R	GTGGTCTCACGAACCACCTTTGTAGAGCTC
ARPC5 F	CACCATGGCAGAATTCGTTGAAGCT
ARPC5 R	AACGGTGTTGATGGTATCAGTAAGAC

Appendix Table S2. DNA oligonucleotides used for genotyping					
**Gene AGI**	**Gene name**		**Mutant**	**Name**	**Sequence 5'-3'**
-	-	-	-	SALK_LBb1.3	ATTTTGCCGATTTCGGAAC
-	-	-	-	SAIL_LB	GCCTTTTCAGAAATGGATAAATAGCCTTGCTTCC
-	-	-	-	GABI_LB_08760	GGGCTACACTGAATTGGTAGCTC
AT1G08370	*DCP1*	*dcp 1-1*	GK_844B03	GK_844B03-LP	TGTTGGTTTCAATTGTTGGATC
				GK_844B03-RP	CAACAGGGATAAAAGGGCTTC
AT1G08370	*DCP1*	*dcp 1-3*	SAIL_377_B10	SAIL_377_B10-RP	CCTCCAGCTCTCACTTCACAG
				SAIL_377_B10-RP	TACAATCACCACCGCCTCTAC
AT5G13570	*DCP2*	*dcp 2-1*	SALK_000519.52.10.x	SALK_000519.52.10.x-LP	TTTGCTATTCTTTGACCTCCG
				SALK_000519.52.10.x-RP	CTCCTTGATGATCTTTGCAGG
AT1G26110	*DCP5*	*dcp 5-1*	SALK_008881	SALK_008881-LP	CCATCAGCAGAGGATGAAGAG
				SALK_008881-RP	GTCCCAAAATTCAAGGCCTAG
AT1G54490	*XRN4*	*xrn4-5*	SAIL_681_E01	SAIL_681_E01-LP	GTTTCTTGGTTGTTGCAGCTC
				SAIL_681_E01-RP	TCATGACGAATTCCTTTGAGG
AT1G05577	*SOK1*	*sok 1*	CRISPR	Forward	CTCGCTGACCGGAAACTGTA
				Reverse	AGCATGGAGTGGCATAAAGATGAAAGAG
AT2G28150	*SOK3*	*sok 3*	CRISPR	Forward	AGGAAGCCCGATTCATTCAGA
				Reverse	AACCGAAATGGCATGGATTC

Appendix Table S3. DNA oligonucleotides used for CRISPR					
**Gene AGI**	**Gene name**	**Mutant**		**Name**	**Sequence 5'-3'**
AT1G05577	*SOK1*	*sok1*	CRISPR	Forward	ATTGCCGGACGGTCGGATGGGAGA
				Reverse	AAACTCTCCCATCCGACCGTCCGG
AT1G05577	*SOK1*	*sok1*	CRISPR	Forward	ATTGTCTCTGCCTCTGGTCAAATA
				Reverse	AAACTATTTGACCAGAGGCAGAGA
AT1G05577	*SOK1*	*sok1*	CRISPR	Forward	ATTGGTTTCTAATCAGACAGTCGC
				Reverse	AAACGCGACTGTCTGATTAGAAAC
AT2G28150	*SOK3*	*sok3*	CRISPR	Forward	ATTGGATCTGGCAGAGAACAATGT
				Reverse	AAACACATTGTTCTCTGCCAGATC
AT2G28150	*SOK3*	*sok3*	CRISPR	Forward	GTCAGATGGACCACTGTTACTGAT
				Reverse	AAACATCAGTAACAGTGGTCCATC
AT2G28150	*SOK3*	*sok3*	CRISPR	Forward	ATTGAGGTCGCCGAGATAAAAACA
				Reverse	AAACTGTTTTTATCTCGGCGACCT

Appendix Figures

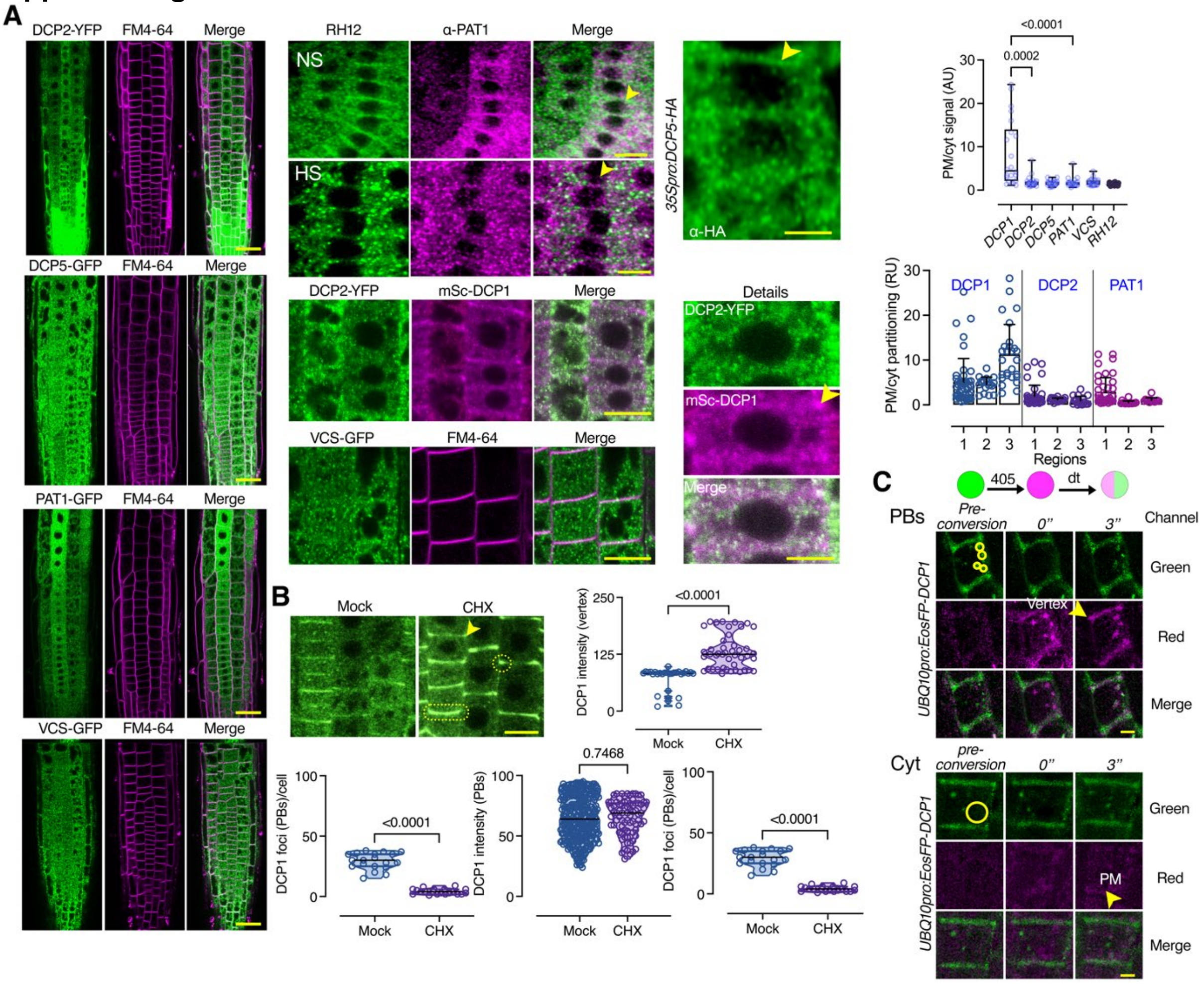

Appendix Fig S1. Unlike DCP1, other core PB components localize at the PM only transiently.

A Left panel: representative confocal micrographs of lines expressing core PB components (DCP2, DCP5, PAT1 and VCS) counterstained with FM4-64 staining (2 µm, 30 min; 4 to 5-day-old seedlings, epidermal cells). Scale bars: 50 µm. Middle panel: representative confocal micrographs showing the transient PM localization of RH12-GFP and PAT1 (detected by α-PAT1) under NS or HS conditions and DCP5 (detected by α-HA, NS condition). Note the absence of DCP1/DCP2 colocalization at the PM in lines coexpressing *RPS5apro:HF-mScarlet-DCP1* and *35Spro:DCP2-YFP* (epidermis cells, region 2). A detailed inset on the right is also shown. Scale bar: 3 µm. The arrowhead points to the likely localization of DCP1 at the PM. No obvious PM/Vertex localization for VCS-GFP (*VCSpro:VCS-GFP*) with FM4-64. Scale bars: 10 µm. Upper right: box plot showing the PM-to-cytoplasmic signal ratio. The ratio of the signal here is approximate, as the PM was defined as a line along the cell contours (*N* = 3, *n* = 15–50 cells). Lower right: box plot showing PM-to-cytoplasmic signal ratio in three root regions of DCP1 (from the *DCP1pro:DCP1-GFP* transgene), DCP2 (*35Spro:DCP2-YFP*) and PAT1 (*PAT1pro:PAT1-GFP* (*N* = 3, *n* = 13–42 cells).

B Representative confocal micrographs showing that cycloheximide (CHX, 50 µm, 30min) treatment does not result in the release of DCP1-GFP from the PM, edges/vertices (region 2, epidermis). Scale bar, 10 µm. The boxplots show DCP1-GFP intensity on edge/vertex, PM, PBs, and DCP1-positive PBs number under control or CHX conditions (50 µM, 30 min; *N* = 3, *n* = 33 cells,). DCP1 intensity in PBs remaining after CHX was calculated in DCP1-positive foci/remnants.

C EosFP-DCP1 tracking in live cell imaging. The monomeric EosFP (Eos: in Greek mythology the personification of the dawn), emits strong green fluorescence (516 nm) that changes to red (581 nm) upon near-UV irradiation at 405 nm because of a photo-induced modification involving a break in the peptide backbone next to the chromophore. The possibility to locally change the emission wavelength makes EosFP a superb marker for experiments aimed at tracking the movements of biomolecules within the living cell. The cartoon shows a PB that is decorated with EosFP-DCP1 and is photoconverted to red. If the signal turns green again, it means that DCP1 molecules are exchanged. Representative confocal micrographs showing the signal conversion of PBs or cytosolic DCP1 (*UBQ10pro:EosFP-DCP1*) (*N, biological replicates* = 2). The circles denote the regions illuminated with 405 nm laser (visible PBs or cytoplasmic residing DCP1). As a cautionary note, the cytoplasmic pool of DCP1 may not be in a dilute phase but could form diffraction-limited PBs. Note the high levels of EosFP-DCP1 signal (red) that shuttle from PBs to the PM, when PBs are photoconverted but not when the dilute phase is photoconverted. This result indicates that DCP1 can shuttle between PBs and the PM, although does not exclude that the cytoplasmic fraction of DCP1 can also shuttle. The offset of PBs post-conversion is due to their movement. Arrowheads denote DCP1 shuffling between the vertex/edge (see later for definition). Scale bars: 5 µm.
Data information: In A, *P* values were determined with nested one-way ANOVA, and in B Wilcoxon. In boxplots or violin plots, upper and lower box boundaries, or lines in the violin plots when visible, represent the first and third quantiles, respectively, horizontal lines mark the median and whiskers mark the highest and lowest values.

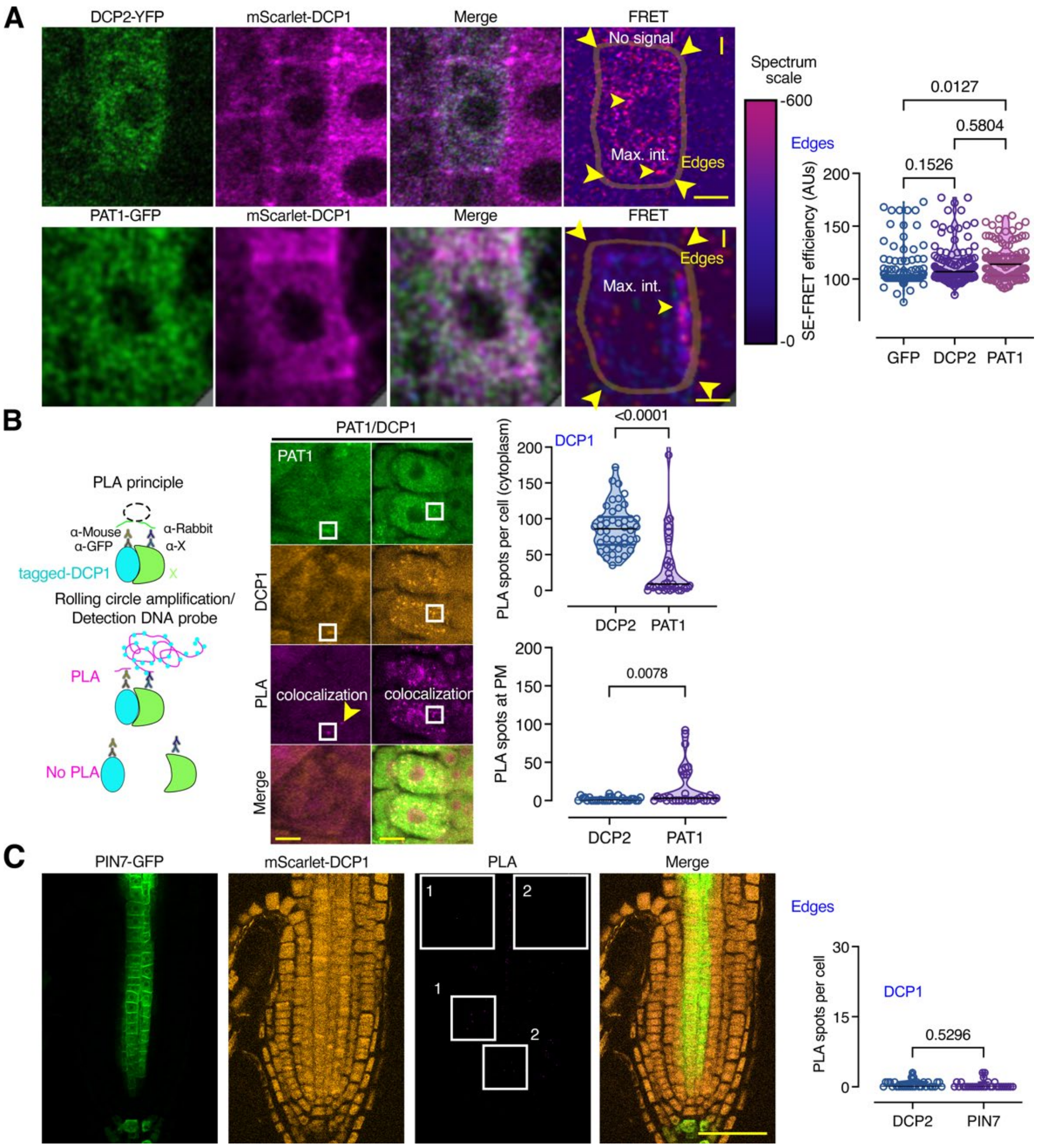

Appendix Fig S2. DCP1/DCP2 or DCP1/PAT1 spatiotemporal interaction by PLA and SE-FRET.

A Sensitized emission (SE)-FRET efficiency between DCP2-YFP and mScarlet-DCP1 in lines co-expressing *RPS5apro:HF-mScarlet-DCP1* and *35Spro:DCP2-YFP* or *PAT1pro:PAT1-GFP* (cell vertices/edges; region 2, epidermal cells, 4 to 5-day-old, *N*=3, *n*=6–8). The cell contours are shown. Right: signal quantification of SE-FRET efficiency between the indicated combinations (DCP1/DCP2 and DCP1/PAT1). As a negative control, lines co-expressing *GFP* and *mScarlet-DCP1* were used. Note also that FRET was observed for puncta in the cytoplasm. Max. int., maximum FRET intensity observed (spectrum scale on the right). Scale bars: 5 µm.

B Left: the principle of the PLA method (see also main text). Duolink® Proximity Ligation Assay (PLA) allows *in situ* detection of endogenous protein interactions with high specificity and sensitivity (theoretical PPI distance <40 nM). Protein targets can be readily detected and localized with single-molecule resolution. Typically, two primary or antibodies against epitope tags raised in different species are used to detect two unique protein targets. A pair of oligonucleotide-labeled secondary antibodies (PLA probes) bind to the primary antibodies. Next, hybridizing connector oligos join the PLA probes only if they are near each other, and ligase forms a closed, circle DNA template that is required for rolling-circle amplification (RCA). The PLA probe then acts as a primer for a DNA polymerase, which generates concatemeric sequences during RCA. This allows up to a 1,000-fold signal amplification that is still tethered

to the PLA probe, allowing localization of the signal and detection of interactions *in situ* with high sensitivity. Last, labeled oligos hybridize to the complementary sequences within the amplicon, which are then visualized and quantified as discrete spots (PLA signals). Middle and right: representative confocal micrographs and a graph showing PLA-positive signal (as PLA spots per cell), produced by α-FLAG/α-GFP for the *HF-mScarlet-DCP1* + PAT1-GFP combination at the PM (all root regions including edge and vertex) and in the cytoplasm (puncta), respectively. Scale bars: 5 µm.

C Representative confocal micrographs and a graph showing PLA signal produced by anti-FLAG/anti-GFP for *the HF-mScarlet-DCP1* + *PIN7-GFP* combination (*N* = 5, *n* = 11, edges/vertices and cytoplasm). This combination represents an additional negative control. PIN7 is a PM protein lacking from the APEAL dataset. The insets (1, 2), show the lack of PLA spots. Some background noise is evident. Scale bar: 100 µm.

Data information: In A, *P* values were determined by Kruskal-Wallis*,* and in B, C unpaired *t*-test (Mann-Whitney produced similar results). Upper and lower lines in the violin plots when visible, represent the first and third quantiles, respectively, horizontal lines mark the median and whiskers mark the highest and lowest values.

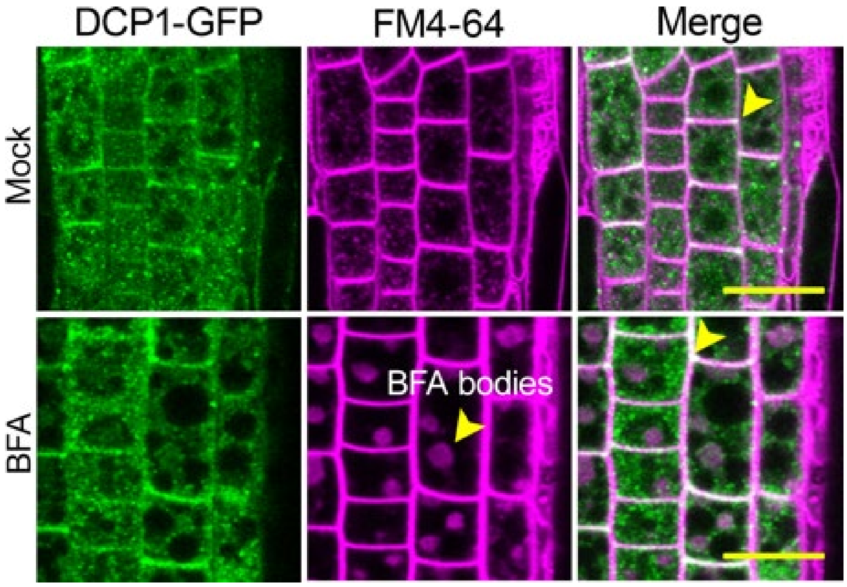

Appendix Fig S3. DCP1 is not strongly accumulating in BFA bodies.

Representative confocal micrographs showing the lack of effect from brefeldin A (BFA; 50 µM for 1 h) treatment on the DCP1-GFP (*DCP1pro:DCP1-GFP*). Scale bars: 20 µm. Arrowhead (top) denotes the edge/vertex, and BFA bodies (stained by FM4-64) are at the bottom. The experiment was performed three times with similar results irrespective of the tissue/cell type. BFA treatment in Arabidopsis roots causes the formation of TGN/endosomal agglomerates called BFA-induced bodies/compartments (Geldner *et al*, 2003). In “Mock”, the endosomes in the cell are stained with FM4-64, and they do not represent small BFA bodies.

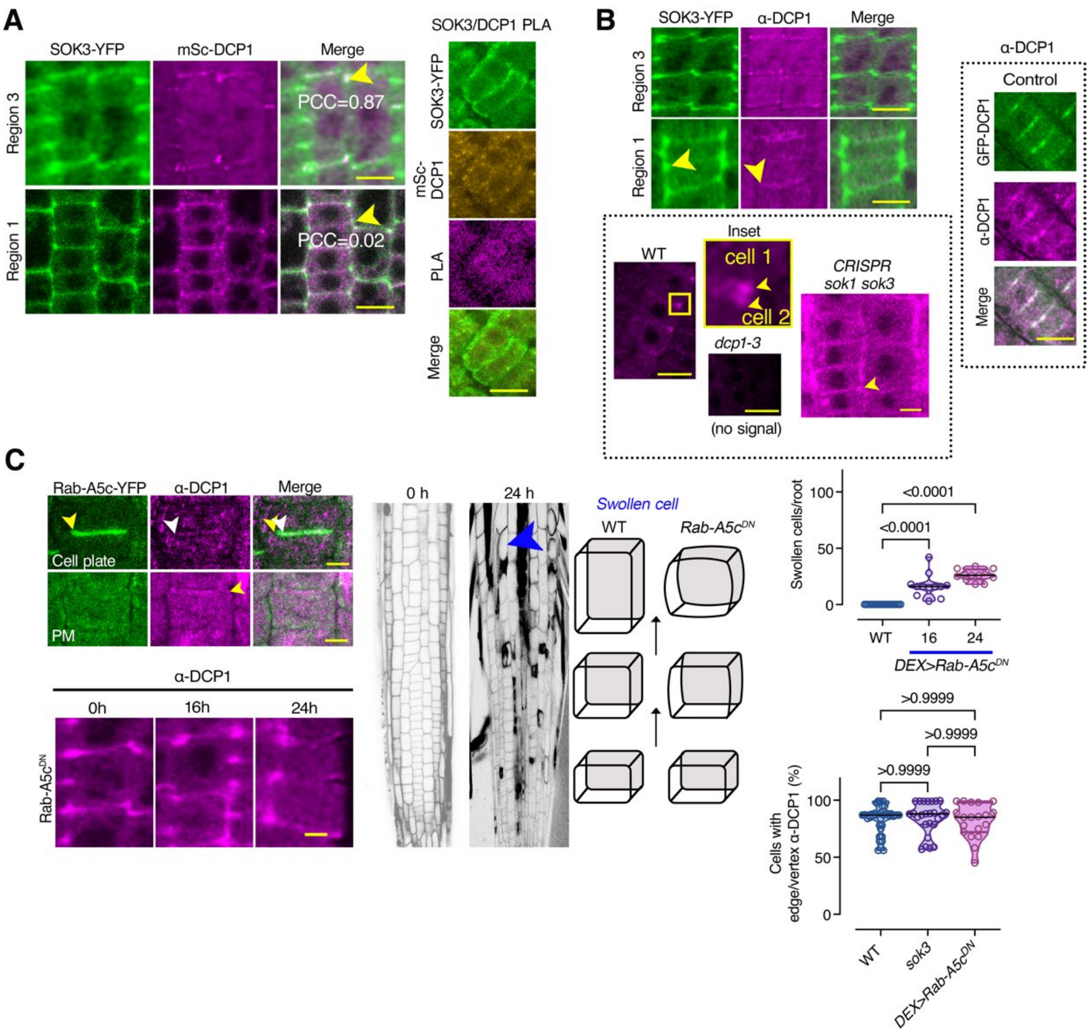

Appendix Fig S4. DCP1 is likely not recruited to the cell edge by Rab-A5c or SOK3.

A Representative confocal micrographs showing the localization of mScarlet-DCP1 (*RPS5apro:HF-mScarlet-DCP1*) and SOK3-YFP (*SOK3pro:SOK3-YFP*) from two regions as indicated (SOK3; left, no abnormal phenotype was observed for the root even for lines expressing *SOK3pro:SOK3-YFP*). The experiment was performed three times with similar results. Scale bars, 10 µm. Right: PLA showing a lack of interaction between DCP1 and SOK3 at the PM (*HF-mScarlet-DCP1* + *SOK3-YFP*; α-FLAG + α-GFP, *N, biological replicates* = 2*;* Arrowheads indicate edges/vertices were DCP1 and SOK3 are not colocalized (in region 1) and colocalized (in region 3)). Scale bars: 10 µm.

B Representative confocal micrographs showing the localization of DCP1, as detected by immunostaining with α-DCP1 (raised against full-length DCP1 protein purified from *Escherichia coli*; details in Materials and methods) and SOK3-YFP (*SOK3pro:SOK3-YFP* transgene; left, no phenotype was observed for the root) from two regions corresponding to 1 and 3. Scale bars: 7 µm. The confocal micrographs in boxes, show the detection of DCP1 using an α-DCP1 antibody in root meristematic epidermal cells of WT seedlings, *dcp1-3* seedlings (with no specific signal), *sok1 sok3 CRISPR*, or seedlings expressing *35Spro:GFP-DCP1* (positive control) (*N, biological replicates* = 2). Note that the loss of SOK3 function does not affect DCP1 localization. The inset shows a detail of the vertex localization of DCP1; the yellow arrowheads point to the gap between two vertices in adjacent cells. Scale bars: 5 µm.

C DCP1 and Rab-5Ac do not colocalize in dividing cells. Representative confocal micrographs showing the localization of DCP1 (detected by anti-DCP1) and Rab5-Ac-YFP in cytokinetic or mesophase cells (*N*=2). The white arrowhead denotes the accumulation of Rab5-Ac-YFP at the leading edge. Note the lack of DCP1 localization at the leading edge (offset of the yellow

arrowhead in “Merge”). In the micrographs below, the edge/vertex is denoted by an arrowhead. Scale bars, 5 µm. Middle: propidium iodide (PI, 10 min)-stained roots showing the radial swelling of a whole root expressing the dexamethasone (DEX)-inducible line *Dex>>RAB-A5c*^{DN} (2 µM DEX, 24 h; blue arrowhead). Under the same conditions, WT roots did not show any swelling. The diagram shows the progressive (an)isotropic growth between WT and *Rab-A5c* inducible lines, respectively. Upper right: a quantification of swollen cells in lines expressing RAB-A5cDN (N = 2, n = 10) is also shown. Lower right: DCP1 detection with the α-DCP1 antibody shows no differential localization of the protein upon dexamethasone induction (2 µM for up to 48 h). Scale bars, 5 µm. The graph indicates the percentage of cells with edge/vertex localization of DCP1 (N = 3, n = 5).
Data information: In C, P were determined by an unpaired t-test. Upper and lower lines in the violin plots when visible, represent the first and third quantiles, respectively, horizontal lines mark the median and whiskers mark the highest and lowest values.

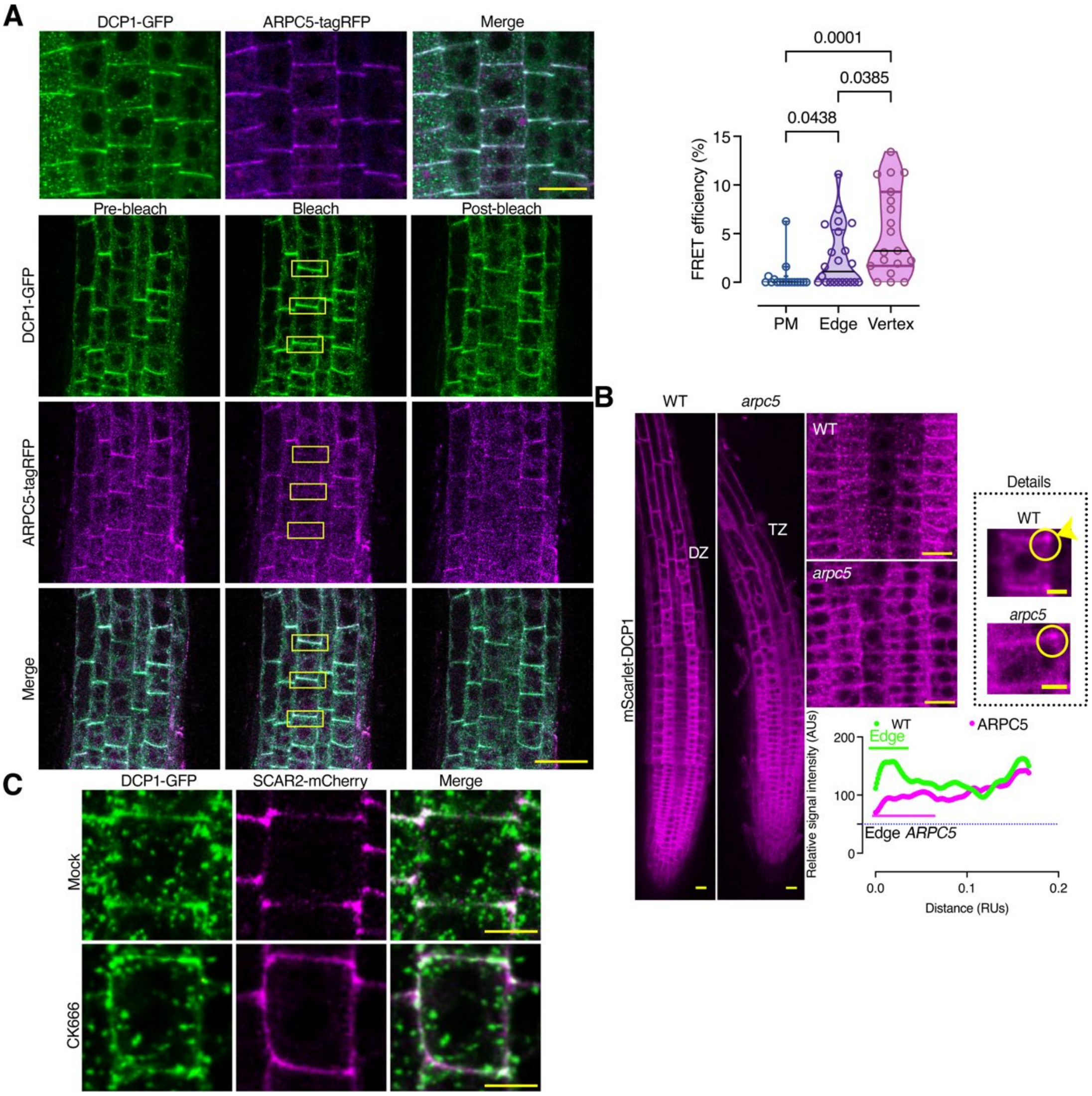

Appendix Fig S5. The colocalization and interaction of DCP1 with ARPC5.

A Representative confocal micrographs showing DCP1-GFP (*DCP1pro:DCP1-GFP*) colocalization with ARPC5-tagRFP with a PCC=0.89 (*RPS5apro:ARPC5-tagRFP*; root region 2 to 3, epidermal cells, 5-day-old seedlings, *N* = 2, *n* = 3). Note the high colocalization at the edges/vertices but not in PBs. Scale bars: 20 µm. Bottom: representative confocal micrographs showing acceptor photobleaching (AB)-FRET efficiency between DCP1-GFP and ARPC5-tagRFP (in lines co-expressing *RPS5apro:HF-mScarlet-DCP1* and ARPC5-tagRFP (cell vertices/edges; region 3, epidermal cells, 4 to 5-day-old, *N* = 4, *n* = 6–8). Right: signal quantification of AB-FRET efficiency at the PM, the edge, or the vertex. Note the overall low FRET efficiency, while in the vertex DCP1 and ARPC5 showed higher but variable interaction. FRET was not observed for puncta in the cytoplasm (was below values for the PM). Scale bars: 50 µm.

B Representative confocal micrographs showing mScarlet-DCP1 localization in WT and the *arpc5* mutant background (region 1 to 4, epidermal cells, 5-day-old seedlings). Note that vertex localization of mScarlet-DCP1 (*RPS5apro:HF-mScarlet-DCP1*) is not significantly reduced by the lack of the ARPC5 (*arpc5*; mutant of ARP2–ARP3), yet the signal is less confined as shown by the plot profile, suggesting that ARP2–ARP3 is required for DCP1 domain restriction at the vertex/edge *(N* = 2, *n* = 6). Here, the *arpc5* allele *crooked* was used, which showed a reduced transition zone (TZ). Scale bars: 10 µm (roots), 3 µm (details).

C Representative confocal micrographs showing the effect of the specific ARP2–ARP3 inhibitor CK-666 (200 µM, 2 h) on DCP1-GFP and SCAR2-mCherry (root region 2, epidermal

cells, 5-day-old seedlings, *N=3, n* = 3). Note the vertex localization of DCP1 did not change much upon the treatment, although the signal was less confined. Scale bars: 7 μm.
Data information: The *P* values in A were determined by Kruskal-Wallis with FDR corrections through a two-stage step-up method of Benjamini, Krieger, and Yekutieli. Upper and lower lines in the violin plots when visible, represent the first and third quantiles, respectively, horizontal lines mark the median and whiskers mark the highest and lowest values.

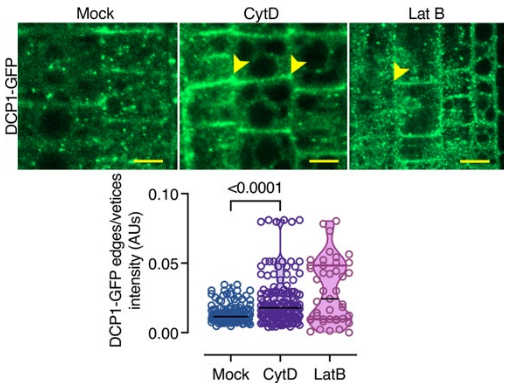

Appendix Fig S6. Cytochalasin D and Latrunculin B effects on DCP1 localization.

Representative confocal micrographs showing the effect of cytochalasin D (Cyt D: binds to the barbed end of actin filaments inhibiting both the association and dissociation of subunits, 5 µM, 1 h) and latrunculin B (Lat B: binds actin monomers near the nucleotide binding cleft with 1:1 stoichiometry and prevents them from polymerizing; 1 µM, 1 h) treatment on DCP1-GFP (*DCP1pro:DCP1-GFP*) localization (*N*=3, *n=4*). SCAR–WAVE increases at the PM in animals upon treatment with actin-depolymerizing drugs (Millius *et al*, 2009). Note that Cyt D reduces DCP1-positive PBs, in contrast to Lat B, which might relate to their differences in function. Arrowheads denote the edge/vertex. Bottom: relative signal intensity of DCP1-GFP in edges/vertices upon Cyt D or Lat B treatment (*N* = 3, *n* = 129–140 edges or vertices, regions 2–3 averaged). Scale bars: 7 µm.

Data information: The *P* values were determined by Wilcoxon. Upper and lower lines in the violin plots when visible, represent the first and third quantiles, respectively, horizontal lines mark the median and whiskers mark the highest and lowest values.

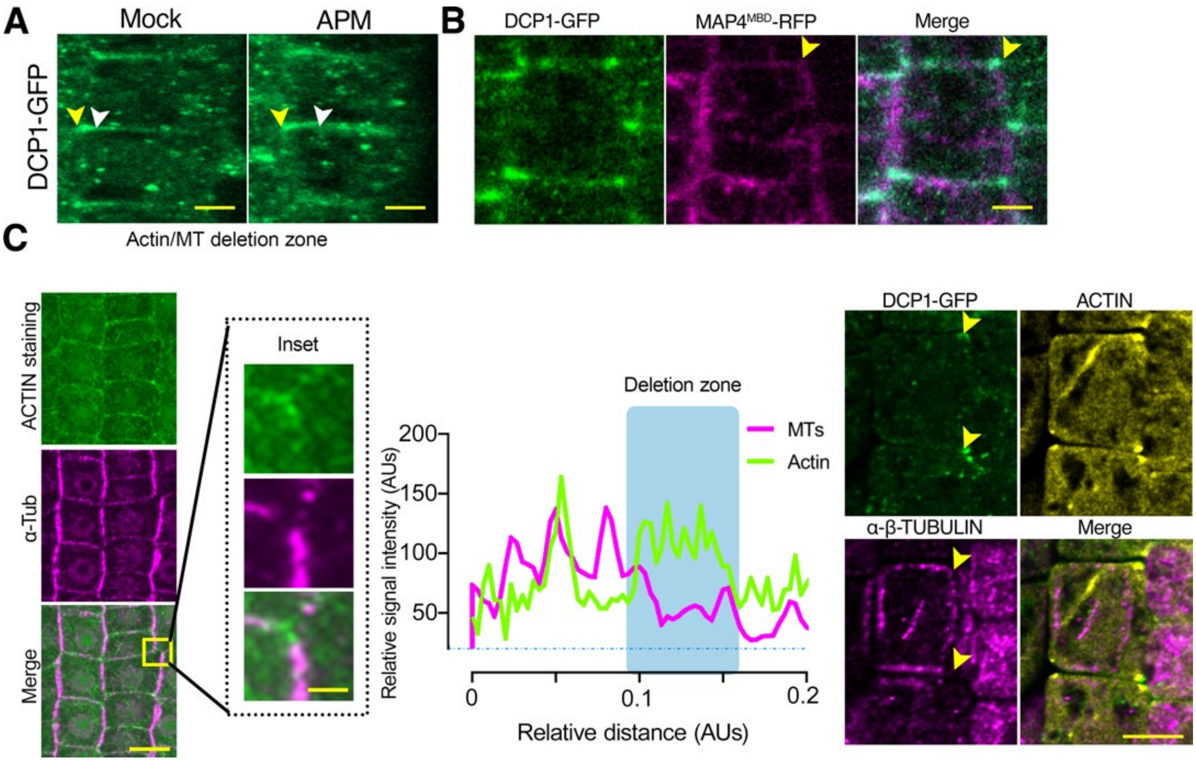

Appendix Fig S7. A microtubule-depleted zone confines DCP1 at the edge/vertex.

A Effect of APM (microtubule depolymerizing drug, 10 μM, 1 h) on DCP1 localization at edges/vertices. Representative confocal image showing the expansion of the DCP1 domain at edges/vertices (the white arrow indicates the expanding edge; epidermal cells of region 2). The expansion was 2.8±1.6 μm (*N* = 5, *n* = 10;). Scale bars: 5 μm.

B Representative confocal micrographs showing DCP1-GFP/MAP-RFP exclusion from lines co-expressing *DCP1pro:DCP1-GFP* and the *MAP4^MBD-RFP* microtubule marker line (*N* = 2, *n* = 10; epidermal cells of region 2). Scale bar, 5 μm.

C Representative confocal micrographs showing root meristematic epidermal cells counterstained simultaneously with phalloidin and α-*β*-tubulin in WT (left side) and α-*β*-tubulin in DCP1-GFP/LifeAct-mCherry line (right side). Scale bars: 10 μm. The inset shows details of the edge (Scale bar, 1 μm). The arrows in the right panel indicated the edge. Note the cell vertex MT-exclusion zone. Middle: plot profile indicating the relative signal intensity of actin and MT staining along the cell edge. The MT deletion zone enriched in actin at the cell edges, as described previously for the trichome apex (Riedl *et al*, 2008). This finding is important, as the loss of ARP2–ARP3 function leads to more variable MT depletion zones in other contexts (Yanagisawa *et al*, 2018), and may explain the variable DCP1 domain size at edges/vertices in the absence of ARPC5 (*arpc5*; mutant of ARP2–ARP3) (Appendix Fig S5).

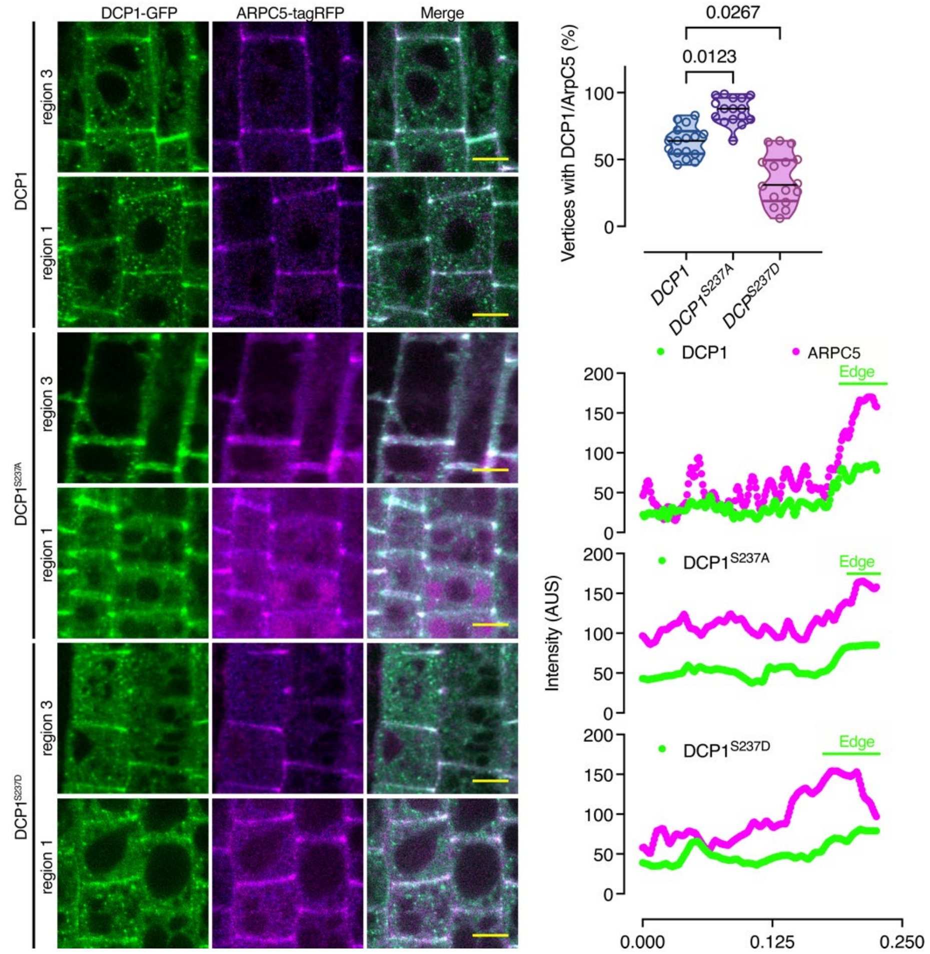

Appendix Fig S8. DCP1 can affect ARPC5 localization and spatial restriction at the edge.

Representative confocal micrographs showing the effect of DCP1 phosphovariants (as indicated in the images) in the localization of ARPC5-tagRFP (*RPS5apro:ARPC5-tagRFP*) in epidermal cells of region 2 or region 3 (meristematic zone and transition zone, respectively, *N* = 3, *n* = *8* cells). Upper right: calculation of the vertices which has both the signal of DCP1-GFP (phosphovariants) and ARPC5-tagRFP. Lower right: plot profiles indicating the relative signal intensity of DCP1-GFP (phosphovariants) and ARPC5-tagRFP signal along the cell edge and vertices. The edge size is shown by the green bar. Scale bars: 10 µm.

Data information: The *P* values here were determined by Friedman (ordinary one-way ANOVA produced even lower *P* values <0.001). Upper and lower lines in the violin plots when visible, represent the first and third quantiles, respectively, horizontal lines mark the median and whiskers mark the highest and lowest values.

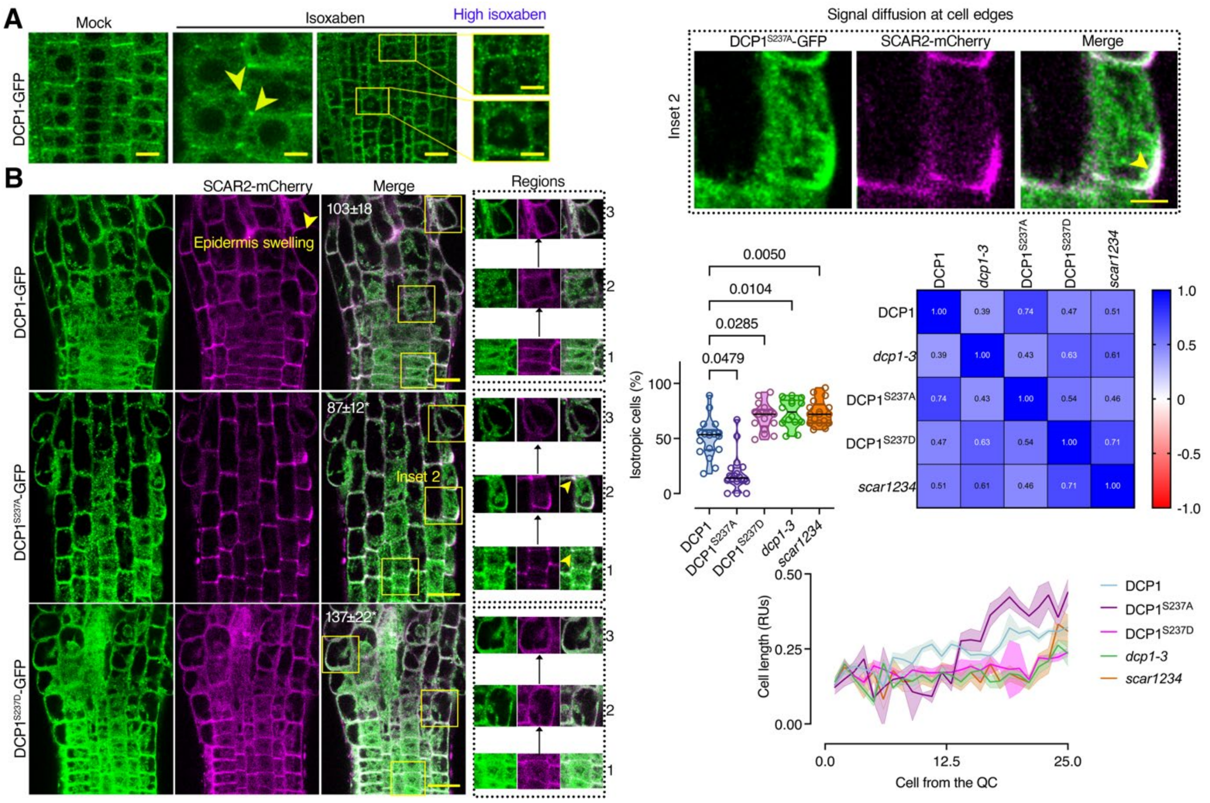

Appendix Fig S9. Effect of isoxaben treatment on isotropic expansion of phosphovariant DCP1 lines and differential expansion of DCP1 phosphovariants, PBs core mutants or SCAR-WAVE mutants.

A Isoxaben ("high isoxaben": IXB, 30 µM, 8 h) slightly affected the vertex localization of DCP1 in some cells, while PBs increased significantly. Arrowheads denote vertex localization of DCP1 (*DCP1pro:DCP1-GFP*; epidermal cells of 5-day-old root, region 3). Scale bars: 10 µm, 5 µm (insets).

B Representative confocal micrographs showing DCP1-GFP (and phosphovariants) localization in the corresponding root meristematic cells treated with isoxaben (IXB, 10 µM, 8 h). Insets indicate treated root cells along the different regions. Note the diffusion of SCAR2-mcherry and DCP1-GFP in cells that due to IXB treatment transition from anisotropic to isotropic expansion (arrowhead in the inset "signal diffusion at the cell edges", $N=3$, $n=9$ roots) of root width. Scale bars: 20 µm. Furthermore, note the detail of DCP1/SCAR2 colocalization (denoted by the arrowhead, scale bar: 10 µm). Right: percentage of cells showing isotropic growth (upper), and their distance from the quiescent center (QC) compared to their length, upon IXB treatment (N = 3, n = 11–21 cells) (lower XY line plot).

Data information: In B, $P<0.05$ was determined by Kruskal-Wallis. The values on micrographs represent mean ± s.d.. The shaded regions in XY line plot (bottom) correspond to the s.d. and the line to the mean. The heat map corresponds to the Pearson r correlation between all X and Y values. Upper and lower lines in the violin plots when visible, represent the first and third quantiles, respectively, horizontal lines mark the median and whiskers mark the highest and lowest values.

Appendix text

Subnetwork 1: RNA metabolism

Nuclear pore complex (NPC) proteins engage in processing, capping, splicing, and polyadenylation; NPC proteins were enriched in PDL/HS. For example, DRACULA (DRA2) is a homolog of mammalian NUP98 and a likely NPC component in Arabidopsis (Gallemi *et al*, 2016). In *Caenorhabditis elegans*, perinuclear P granules contain components of PBs and SGs; P granules at NPC clusters might interact with nascent RNAs (Sheth *et al*, 2010). As the interaction of PBs with NPC occurs under HS conditions, PBs may be recruited there to store nascent RNAs or block them from nuclear export. Furthermore, mRNAs undergo several modifications; one of the most prevalent is N^6-methyladenosine (m^{6}A), which is of key importance in development. Both AP/PDL datasets (mainly HS; Supplementary File 6) contained the YTH (YT521-B homology)-domain ECT (EVOLUTIONARILY CONSERVED C-TERMINAL REGION) proteins, which are praise *bona fide* m^{6}A readers controlling leaf development (Arribas-Hernandez *et al*, 2018). The ECT animal homologs, YTHDF1/2/3, undergo LLPS induced by polymethylated mRNAs acting as a multivalent scaffold for their binding, juxtaposing their low-complexity domains and thereby leading to LLPS(Zaccara *et al*, 2019). We identified components of the adenylation regulatory machinery, including the CARBON CATABOLITE REPRESSION 4 (CCR4)–NOT complex and POLYADENYLATE BINDING PROTEINs (PABs). For PDL/HS, we identified the PAB-interacting protein CTC-INTERACTING DOMAIN 3 (CID3), a homolog of ataxin-2 identified in mammalian PBs (Nonhoff *et al*, 2007). At3g45630 is a putative ubiquitin (Ub) E3 ligase with an RRM (RNA-recognition motif) (de Meaux *et al*, 2008), showing architectural similarity to the RING E3 ligase NOT4; we confirmed through BLAST-P analysis its orthology to human NOT4 (p=6e^{-59}), which catalyzes the removal of the poly(A) tail. Likewise, CCR4 localizes to plant PBs(Zhang *et al*, 2020). Ub, as well as the proteasome itself, enters condensates (Yasuda *et al*, 2020), and thus it is not surprising that E3 ligases are recruited to PBs. Furthermore, we identified argonaute1 (AGO1) in the PDL (Fig 1B), involved in RNA metabolism. As AGO1 is involved in nascent peptide ubiquitination (Gao *et al*, 2022), this function could also involve PBs and associated E3 ligases.

Subnetwork 2: Defense Regulation and Metabolism

The NF-X1-type zinc finger protein NFXL1 (PDL/HS) functions as a negative regulator of defense-related genes via a salicylate (SA)-dependent signaling pathway. Likewise, NECROTIC SPOTTED LESIONS 1 (NSL1), a MACPF domain-containing protein, negatively controls the SA-mediated cell death (Noutoshi *et al*, 2006). We also identified three proteins with glycine-tyrosine-phenylalanine (GYF; PDL/HS) domains; one, Essential for poteXvirus Accumulation 1 (EXA1), recognizes Pro-rich sequences, as do other GYF domain-containing proteins found in humans and yeast (Matsui *et al*, 2017). EXA1 is indispensable for *Plantago asiatica* mosaic virus infection and negatively regulates levels of plant immune receptors via translational repression. EXA1 also interacts with SMG7 (also enriched in the PDL/HS), which plays a role in the nonsense-mediated RNA decay (NMD) (Matsui *et al.*, 2017). Two additional GYF-containing proteins identified are of unknown function (At1g24300 and At1g27430). These findings suggest that PBs restrain cell death via immune signaling and may function as a hub in SA perception. We identified proteins involved in translational regulation, e.g., deoxyhypusine synthase (At5g05920; AP)(Moschou & Roubelakis-Angelakis, 2014). This enzyme mediates the post-translational activation of eIF5A, which facilitates the translation of specific mRNAs. This confinement in PBs may partition deoxyhypusine synthase into an inactive pool to reduce translation and minimize energy squandering during stress.

Subnetwork 3: Membrane remodeling/trafficking

Unexpectedly, we detected significant enrichment for membrane-associated proteins in the PDL proximitome. This subnetwork was further divided into four major dense clades consisting of 1) ESCRT (endosomal sorting complex required for transport), 2) dynamin-related proteins

(DRPs) and 3) the plant-specific endocytic TPLATE complex (see also Fig 2A). DRPs are force-generating proteins targeted to membranes that regulate membrane remodeling pathways (Fujimoto *et al*, 2010). We observed an enrichment of DRPs (i.e., 2A/B/E/C/E, ARC5 and RSW9) for both AP and PDL. Furthermore, DRPs are physically linked to the TPLATE complex (Arora *et al*, 2020), and enriched under HS conditions (e.g., AtEH2, $\log_2FC_{NS/HS}$=0/3.1). Recently AtEH1 was shown to phase-separate to induce endocytosis(Dragwidge *et al*, 2022). We hypothesize that during stress, DCP1 may cooperate with the endocytotic machinery. This hypothesis is possible, considering that eisosomes, static structures that arguably modulate endocytosis, contain the XRN4 yeast homolog XRN1 (Grousl *et al*, 2015). The complexes ESCRT-0 -I, -II, and -III drive Ub-mediated cargo sorting from the PM (Xia *et al*, 2020). ESCRT components were enriched in the PDL: e.g., ELC (Vps23p/TSG101, multivesicular bodies [MVB] sorting), VPS28/37 (ESCRTI complex) containing a non-canonical RNA-binding motif, and the VPS15, which sort endocytic cargos into MVBs. In animals, ESCRT components have been found in condensates (von Appen *et al*, 2020). DCP1 or the PBs may aid in the formation of MVBs or load them with specific RNA molecules. Furthermore, we identified proteins residing in interorganellar contact sites: for example, Annexin 1 (ANNAT1; PDL/NS; 17 peptides vs. 0 in the control). In animals, Annexin A11 (ANXA11) is an RNA granule-associated phosphoinositide-binding protein that functions as a molecular tether between RNPs and membranes(Liao *et al*, 2019). Likewise, in the endosome-vesicular trafficking proteins, the ORP2A, OSBP (OXYSTEROL BINDING PROTEIN)-RELATED PROTEIN ORP-related Osh lipid exchange proteins were found enriched in PDL. ORPs create a potential nanoscale membrane lipid environment (Nishimura *et al*, 2019) controlling PM organization and dynamics and marking ER-PM contact sites (ERPC), while in animal cells LLPS in ORP-decorated sites marks contacts with peroxisomes and lysosomes(King *et al*, 2020; Wang *et al*, 2019).

Subnetwork 4: Novel Interactors: Actin polymerization and cytoskeleton remodeling

With regards to actin, we identified SCAR–WAVE components (cluster score $p=5.41e^{-5}$; FDR=0.05) and accessory regulators like PIR121 and NAP125 (NAP1; both in PDL/HS, see also below), which collectively modulate actin/microtubules dynamics (Li *et al*, 2004). Likewise, for microtubules (MTs), we identified nucleators like TONNEAU1 (TON1) that share similarities with the human centrosomal protein FOP (FGFR1 Oncogene Partner). TON1 interacts with a superfamily of 34 proteins conserved in land plants that harbor the TON1A/B Recruiting Motif (TRM) involved in spindle and phragmoplast organization (Drevensek *et al*, 2012). TRM proteins involve the evolutionarily conserved γ-tubulin complex (TuRC); in agreement with this, we identified NEDD1, which interacts with TuRC (Zeng *et al*, 2009). In animals and fungi, γ-tubulin is a nucleator localizing in the MT organizing center (the centrosome in animal cells and the spindle pole body in yeast).

References

Arora D, Abel NB, Liu C, Van Damme P, Yperman K, Eeckhout D, Vu LD, Wang J, Tornkvist A, Impens F *et al* (2020) Establishment of Proximity-Dependent Biotinylation Approaches in Different Plant Model Systems. *Plant Cell* 32: 3388-3407

Arribas-Hernandez L, Bressendorff S, Hansen MH, Poulsen C, Erdmann S, Brodersen P (2018) An m(6)A-YTH Module Controls Developmental Timing and Morphogenesis in Arabidopsis. *Plant Cell* 30: 952-967

de Meaux J, Hu J-Y, Tartler U, Goebel U (2008) Structurally different alleles of the ath-MIR824 microRNA precursor are maintained at high frequency in Arabidopsis thaliana. *Proceedings of the National Academy of Sciences* 105: 8994-8999

Dragwidge JM, Wang Y, Brocard L, De Meyer A, Hudeček R, Eeckhout D, Chambaud C, Pejchar P, Potocký M, Vandorpe M *et al* (2022) AtEH/Pan1 proteins

drive phase separation of the TPLATE complex and clathrin polymerisation during plant endocytosis. *bioRxiv*: 2022.2003.2017.484738
Drevensek S, Goussot M, Duroc Y, Christodoulidou A, Steyaert S, Schaefer E, Duvernois E, Grandjean O, Vantard M, Bouchez D *et al* (2012) The Arabidopsis TRM1-TON1 interaction reveals a recruitment network common to plant cortical microtubule arrays and eukaryotic centrosomes. *Plant Cell* 24: 178-191
Fujimoto M, Arimura S, Ueda T, Takanashi H, Hayashi Y, Nakano A, Tsutsumi N (2010) Arabidopsis dynamin-related proteins DRP2B and DRP1A participate together in clathrin-coated vesicle formation during endocytosis. *Proc Natl Acad Sci U S A* 107: 6094-6099
Gallemi M, Galstyan A, Paulisic S, Then C, Ferrandez-Ayela A, Lorenzo-Orts L, Roig-Villanova I, Wang X, Micol JL, Ponce MR *et al* (2016) DRACULA2 is a dynamic nucleoporin with a role in regulating the shade avoidance syndrome in Arabidopsis. *Development* 143: 1623-1631
Gao Y, Zhu Y, Wang H, Cheng Y, Zhao D, Sun Q, Chen D (2022) Lipid-mediated phase separation of AGO proteins on the ER controls nascent-peptide ubiquitination. *Mol Cell*
Geldner N, Anders N, Wolters H, Keicher J, Kornberger W, Muller P, Delbarre A, Ueda T, Nakano A, Jurgens G (2003) The Arabidopsis GNOM ARF-GEF mediates endosomal recycling, auxin transport, and auxin-dependent plant growth. *Cell* 112: 219-230
Grousl T, Opekarová M, Stradalova V, Hasek J, Malinsky J (2015) Evolutionarily Conserved 5'-3' Exoribonuclease Xrn1 Accumulates at Plasma Membrane-Associated Eisosomes in Post-Diauxic Yeast. *Plos One* 10: e0122770
King C, Sengupta P, Seo AY, Lippincott-Schwartz J (2020) ER membranes exhibit phase behavior at sites of organelle contact. *Proc Natl Acad Sci U S A* 117: 7225-7235
Li Y, Sorefan K, Hemmann G, Bevan MW (2004) Arabidopsis NAP and PIR regulate actin-based cell morphogenesis and multiple developmental processes. *Plant Physiol* 136: 3616-3627
Liao Y-C, Fernandopulle MS, Wang G, Choi H, Hao L, Drerup CM, Patel R, Qamar S, Nixon-Abell J, Shen Y *et al* (2019) RNA Granules Hitchhike on Lysosomes for Long-Distance Transport, Using Annexin A11 as a Molecular Tether. *Cell* 179: 147-164.e120
Matsui H, Nomura Y, Egusa M, Hamada T, Hyon G-S, Kaminaka H, Watanabe Y, Ueda T, Trujillo M, Shirasu K *et al* (2017) The GYF domain protein PSIG1 dampens the induction of cell death during plant-pathogen interactions. *PLoS genetics* 13: e1007037-e1007037
Millius A, Dandekar SN, Houk AR, Weiner OD (2009) Neutrophils Establish Rapid and Robust WAVE Complex Polarity in an Actin-Dependent Fashion. *Current Biology* 19: 253-259
Moschou PN, Roubelakis-Angelakis KA (2014) Polyamines and programmed cell death. *J Exp Bot* 65: 1285-1296
Nishimura T, Gecht M, Covino R, Hummer G, Surma MA, Klose C, Arai H, Kono N, Stefan CJ (2019) Osh Proteins Control Nanoscale Lipid Organization Necessary for PI(4,5)P2 Synthesis. *Mol Cell*
Nonhoff U, Ralser M, Welzel F, Piccini I, Balzereit D, Yaspo M-L, Lehrach H, Krobitsch S (2007) Ataxin-2 interacts with the DEAD/H-box RNA helicase DDX6 and interferes with P-bodies and stress granules. *Molecular biology of the cell* 18: 1385-1396

Noutoshi Y, Kuromori T, Wada T, Hirayama T, Kamiya A, Imura Y, Yasuda M, Nakashita H, Shirasu K, Shinozaki K (2006) Loss of Necrotic Spotted Lesions 1 associates with cell death and defense responses in Arabidopsis thaliana. *Plant Mol Biol* 62: 29-42

Riedl J, Crevenna AH, Kessenbrock K, Yu JH, Neukirchen D, Bista M, Bradke F, Jenne D, Holak TA, Werb Z *et al* (2008) Lifeact: a versatile marker to visualize F-actin. *Nature methods* 5: 605-607

Sheth U, Pitt J, Dennis S, Priess JR (2010) Perinuclear P granules are the principal sites of mRNA export in adult C. elegans germ cells. *Development* 137: 1305-1314

von Appen A, LaJoie D, Johnson IE, Trnka MJ, Pick SM, Burlingame AL, Ullman KS, Frost A (2020) LEM2 phase separation promotes ESCRT-mediated nuclear envelope reformation. *Nature* 582: 115-118

Wang P, Pleskot R, Zang J, Winkler J, Wang J, Yperman K, Zhang T, Wang K, Gong J, Guan Y *et al* (2019) Plant AtEH/Pan1 proteins drive autophagosome formation at ER-PM contact sites with actin and endocytic machinery. *Nat Commun* 10: 5132

Xia F-N, Zeng B, Liu H-S, Qi H, Xie L-J, Yu L-J, Chen Q-F, Li J-F, Chen Y-Q, Jiang L *et al* (2020) SINAT E3 Ubiquitin Ligases Mediate FREE1 and VPS23A Degradation to Modulate Abscisic Acid Signalling. *The Plant Cell*: tpc.00267.02020

Yanagisawa M, Alonso JM, Szymanski DB (2018) Microtubule-Dependent Confinement of a Cell Signaling and Actin Polymerization Control Module Regulates Polarized Cell Growth. *Curr Biol* 28: 2459-2466 e2454

Yasuda S, Tsuchiya H, Kaiho A, Guo Q, Ikeuchi K, Endo A, Arai N, Ohtake F, Murata S, Inada T *et al* (2020) Stress- and ubiquitylation-dependent phase separation of the proteasome. *Nature* 578: 296-300

Zaccara S, Ries RJ, Jaffrey SR (2019) Reading, writing and erasing mRNA methylation. *Nat Rev Mol Cell Bio* 20: 608-624

Zeng CJ, Lee YR, Liu B (2009) The WD40 repeat protein NEDD1 functions in microtubule organization during cell division in Arabidopsis thaliana. *Plant Cell* 21: 1129-1140

Zhang Z-J, Gao Q, Fang X-D, Ding Z-H, Gao D-M, Xu W-Y, Cao Q, Qiao J-H, Yang Y-Z, Han C *et al* (2020) CCR4, a RNA decay factor, is hijacked by a plant cytorhabdovirus phosphoprotein to facilitate virus replication. *eLife* 9: e53753
