# Supplementary material for: An actin remodeling role for Arabidopsis processing bodies revealed by their proximity interactome
Source: EMBO J. 2023 Feb 6;42(9):e111885. doi: 10.15252/embj.2022111885 (PMC10152145; doi:10.15252/embj.2022111885)
Supplement: Supplementary file 2 — Expanded View Figures PDF [file EMBJ-42-e111885-s003.pdf]

Expanded View Figures

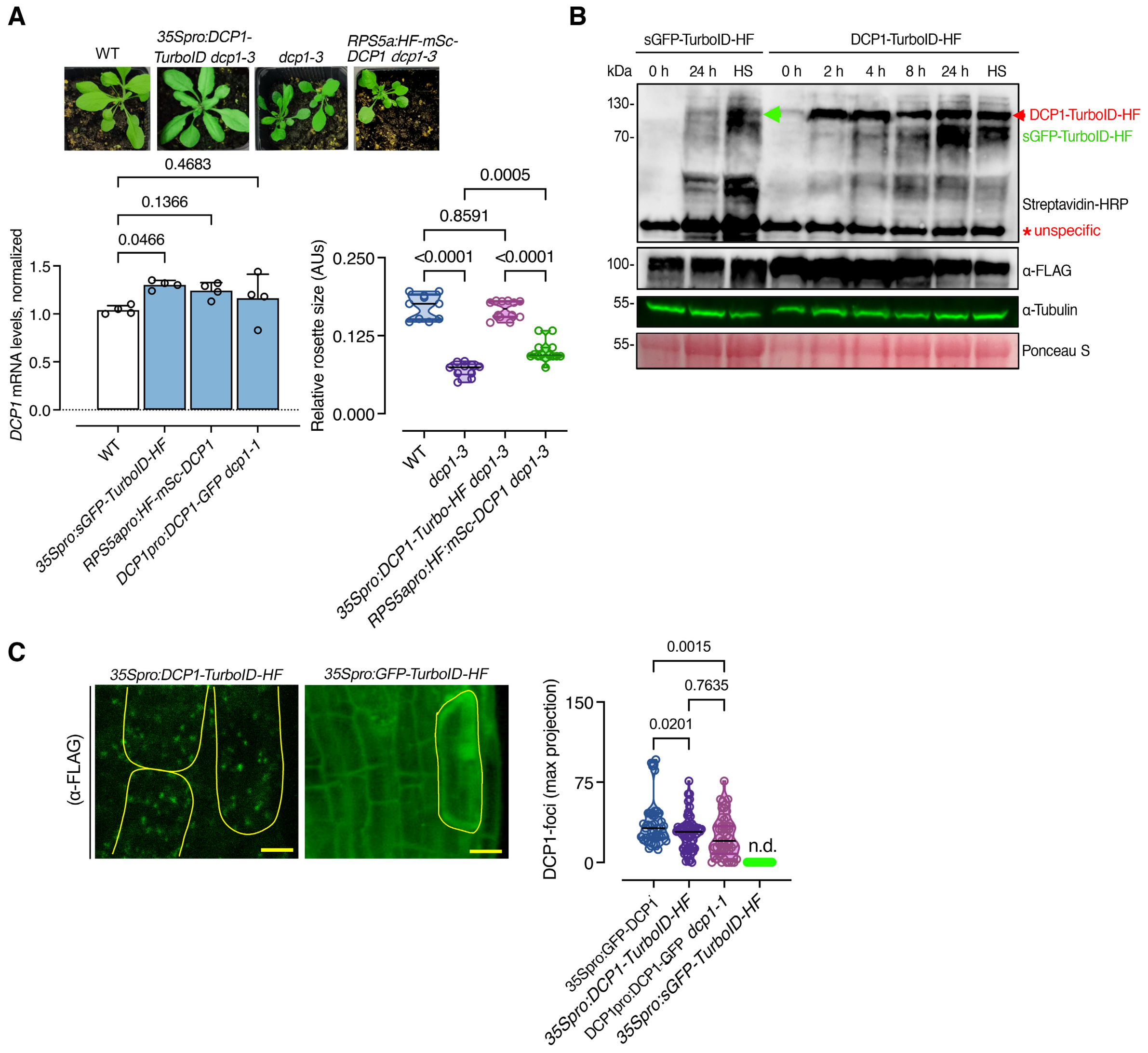

Figure EV1.

© 2023 The Authors

◀ **Figure EV1. Establishment of a functional DCP1 bait for PDL.**

A The *DCP1-TurboID-HF* construct efficiently rescues the adult *dcp1-3* mutant phenotype. Phenotypes (3-week-old) of adult plants expressing *35Spro:DCP1-TurboID-HF* and *RPS5apro:HF-mScarlet-DCP1* in the *dcp1-3* mutant background. In a semi-controlled greenhouse setting (temperature control 22°C), the *dcp1-3* mutant showed a smaller adult stature. Lower: complementation quantification (rosette diameter), *N*, *biological replicates* = 4, *n* = (*pooled data of 3 biological replicates*) 5–7 rosettes, error bars are (mean + SD) and RT-qPCR analyses for the quantification of *DCP1* expression levels. *PP2A* and *Actin7* were used for normalization (1-week-old) seedlings (*N*, *biological replicates* = 2, *n* (*pooled data of 3 biological replicates*) = 3, error bars are mean + SD). When DCP1 was driven by the RPS5a promoter (stem cell-specific promoter), we observed partial complementation, suggesting that DCP1 is important also in non-meristematic cells.

B Immunoblot analyses of lines expressing sGFP-TurboID-HF or DCP1-TurboID-*HF* upon NS or HS conditions (same as used for APEAL). The immunoblots also show the accumulation of auto-biotinylated sGFP-TurboID-HF or DCP1-TurboID-HF in a time course of biotin administration (as detected with streptavidin-HRP, which captures biotinylated proteins). 50 μM Biotin was delivered in leaves by syringe infiltration and diffusion. Note that HS did not increase the biotinylation efficiency of DCP1-TurboID-HF: at 24 h, compare samples “2” with “4” in the “DynaIP”. HF, 6xhis-3xFLAG. The red arrowhead indicates the position of DCP1-TurboID-HF and green for sGFP-TurboID-HF (*N*, *biological replicates* = 2). We used the same scheme for biotin application as determined in (Arora *et al*, 2020). The 2 h NS/HS corresponds to the timing after the administration of biotin for 24 h (*t* = 0 corresponds to 24 h biotin administration in NS conditions). The red asterisk indicates non-specific band. α-FLAG was used for the detection of DCP1-TurboID-HF and sGFP-TurboID-HF (similar size ~ 130 kDa). α-Tubulin and ponceau staining were used for loading control.

C Representative confocal micrographs showing that the localization of DCP1 (α-FLAG, 5-day-old seedlings, root cap cells) to PBs is retained in lines expressing *DCP1-TurboID-HF*. As a control, the *GFP-TurboID-HF* line was used that does not show localization to PBs. Right: number of DCP1-positive foci in the corresponding lines expressing *DCP1* (*N*, *biological replicates* = 1, *n* = 32–60 cells).

Data information: In (A and B), *P* values were determined by ordinary one-way ANOVA. Upper and lower lines in the violin plots when visible, represent the first and third quantiles, respectively, horizontal lines mark the median and whiskers mark the highest and lowest values.
Source data are available online for this figure.

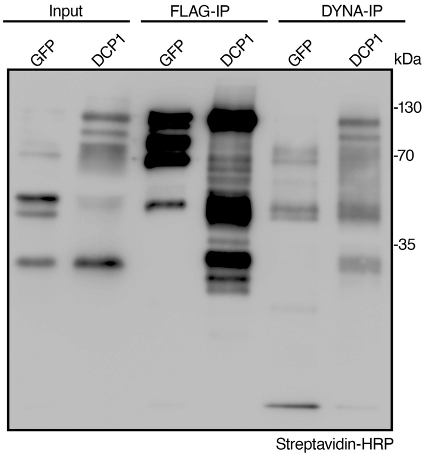

Figure EV2. Biotinylated proteins evade purification from the AP step in APEAL.

Immunoblot analyses from lines expressing *GFP-TurboID-HF* or *DCP1-TurboID-HF* (denoted as GFP or DCP1, respectively) showing the presence of biotinylated proteins in the flow-through after the AP-step (for flow-through 1, see DYNA-IP here). The blot was overexposed to detect the faint streptavidin smear in flow-through 1. The baits in these experiments undergo auto-biotinylation, as reported previously. The results are representative of one experiment performed three times. Note that the immunodetectable biotinylation levels did not correlate well with the hits identified in Fig EV5 (compare GFP to DCP1). GFP and DCP1 are of similar molecular weight, while the bands below the upper band (~ 130 kDa) likely correspond to proteolytic cleavage products.

 © 2023 The Authors

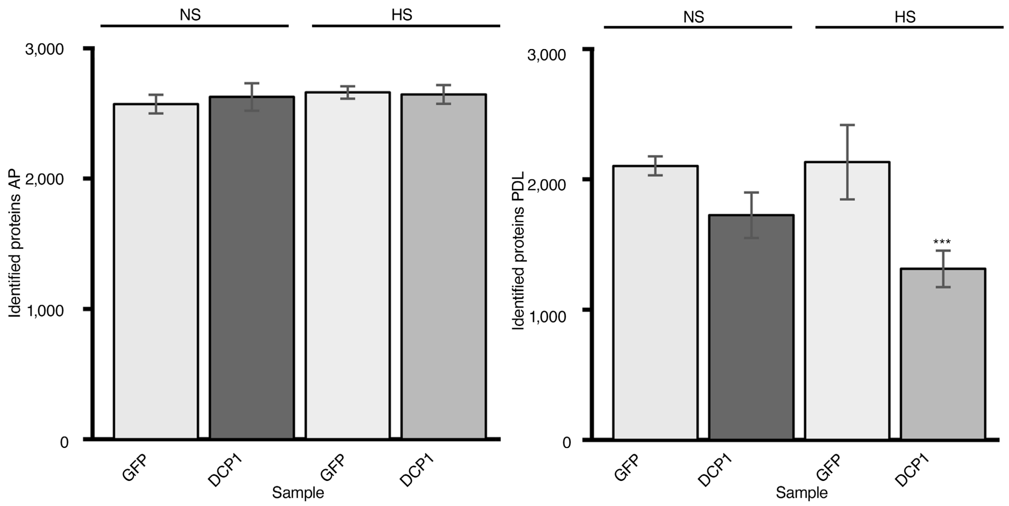

Figure EV3. Hits from the APEAL approach (AP and PDL steps).

Total protein hits from mass spectrometry under NS or HS conditions following the AP (left) or PDL (right) steps of APEAL. We used the same scheme for biotin application, as described (Arora *et al*, 2020). The results presented are unfiltered, containing the noisy portion of the proteome. Note that the free diffusion of GFP *in vivo* led to increased proteins identified. *sGFP-TurboID-HF*, GFP; *DCP1-TurboID-HF*, DCP1 (*N*, *biological replicates* = 3, error bars are mean ± SD). Note the increased numbers of hits in GFP/PDL reflect the noisy proteome. GFP is expected to produce more noise (translated as hits in the context of the proteome), due to the increased diffusion over the specifically and topologically restricted DCP1 (confirmed in Fig EV1, localizations). The decreased number of hits for HS conditions corresponds mainly to proteins of signal transduction and metabolism, as well as vesicle trafficking proteins (see also Fig 2 and below for an explanation: HS reduces the DCP1 association with the PM). Furthermore, DCP1, as described below, loses localization at the PM during HS.

Data information: AP/PDL GFP samples did not differ at $P < 0.005$ (determined by an unpaired *t*-test); ***$P \leq 0.05$, as determined by an unpaired *t*-test for comparison between HS and NS GFP samples.

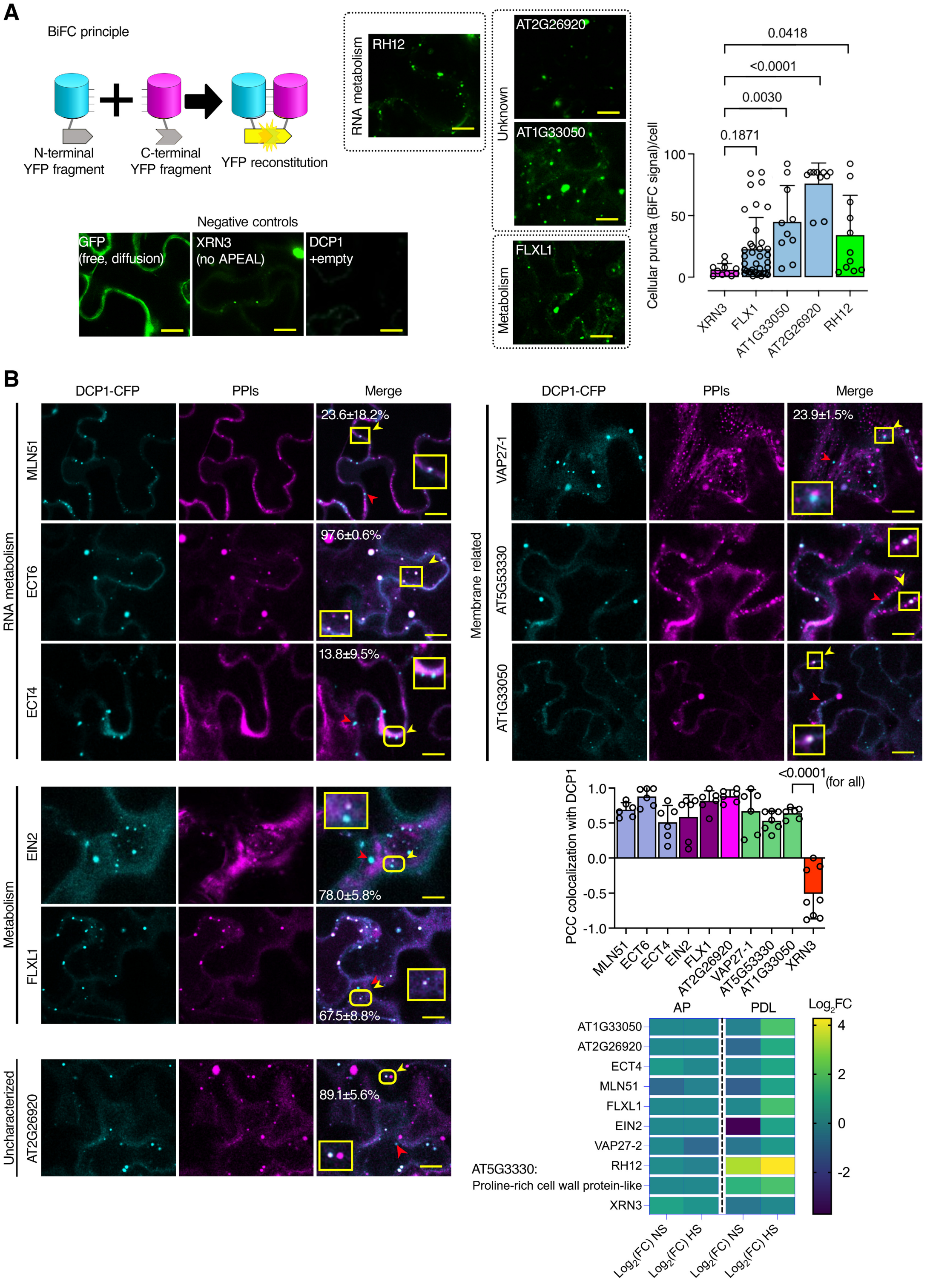

Figure EV4.

 © 2023 The Authors

◀ **Figure EV4. DCP1 colocalization and association with novel and known interactors in transient expression of N. benthamiana leaves.**

A Bimolecular fluorescence complementation (BiFC) assay of the indicated proteins. Left: the cartoon depicts the BiFC concept and YFP reconstitution caused by protein–protein interaction *in vivo*. When two proteins interact, the cYFP and nYFP halves are brought in proximity and produce a fluorescent signal. For protein selection, we classified the associations with DCP1, in both AP and PDL steps, according to their relative enrichment (log_2FC). We selected proteins presenting moderate relative enrichment in the AP step, while having high predictability in the PDL step ($log_2FC > 0$ in both steps). As an additional filter, we selected proteins rich in IDRs (half of the proteins from the list have high prion-like domain (PRD) scores and high Finum [aa]/total [aa] ratios as defined through the PLAAC algorithm; Source File 8), and as such proteins would be likely to localize to condensates. We tested the association between PBs (using DCP1 as one representative component) and selected five proteins from these bins using BiFC and colocalization assays (Source File 8). These results suggested that the APEAL approach may help identify PB components or DCP1 interactors. These interactions should be further studied in *Arabidopsis*. BiFC efficiency was estimated from the reconstituted YFP raw signal intensity and YFP-positive puncta per cell in maximum projections (*N*, *biological replicates* = 3, *n* = 4–12 cells). Lower left: representative confocal micrographs showing that YFP signal is reconstituted at cellular puncta that most likely correspond to PBs. XRN3 represents negative control (see also B), as it localizes in the nucleus. Right: number of cellular puncta per total cell volume (in maximum projection images; *N*, *biological replicates* = 3, *n* (*pooled data of 3 biological replicates*) = 20 cells, error bars are mean + SD). Scale bars: 10 μm.

B Colocalization of selected proteins with DCP1-CFP-positive puncta (*35Spro:DCP1-CFP* transgene). The coding sequences of the corresponding “interactors” (PPIs; direct or indirect, defined in APEAL) were driven by the *35Spro* and cloned in frame with *mCherry* at their 5′ end. Two-color colocalizations were estimated by Pearson’s correlation coefficients (PCC) using ultra-fast super-resolution microscopy combined with image deconvolution (~ 120 nm axial resolution). Numbers in “merge” indicate colocalization frequency between DCP1-CFP and the corresponding interacting protein (*N*, *biological replicates* = 2, *n* = 5 cells). Yellow arrowheads indicate colocalization and red arrowheads lack of colocalization. Lower right: PCC of pixel intensities between DCP1-CFP and the corresponding putative interacting protein (*N*, *biological replicates* = 3, *n* = 4–12 cells, error bars are mean + SD). We confirmed the ECT domain-containing proteins, MLN51, FLXL1, EIN2, VAP27-1, uncharacterized AT1G33050 (hypothetical protein), and AT5G53330/AT2G26020 (ubiquitin-associated/translation elongation factors EF1B) as novel PB components. By applying a pre-selection criterion of enrichment ($log_2FC > 0.5$) for the selection of prey, we significantly increased the probability of identifying successful binary interactions between PBs (i.e., cYFP-DCP1) and the identified proteins. All preys were confirmed as PB components, while PDL had higher interaction predictive power than the AP step irrespective of whether proteins were enriched in NS or HS. XRN3 was used as a threshold control ($log_2FC = -0.58$, PDL/NS conditions). The heatmap shows the enrichment of these proteins in the different conditions; the scale at right shows log_2FC.

Data information: In (A and B), *P* values were determined by ordinary one-way ANOVA (differences were all calculated compared to XRN3).
Source data are available online for this figure.

© 2023 The Authors

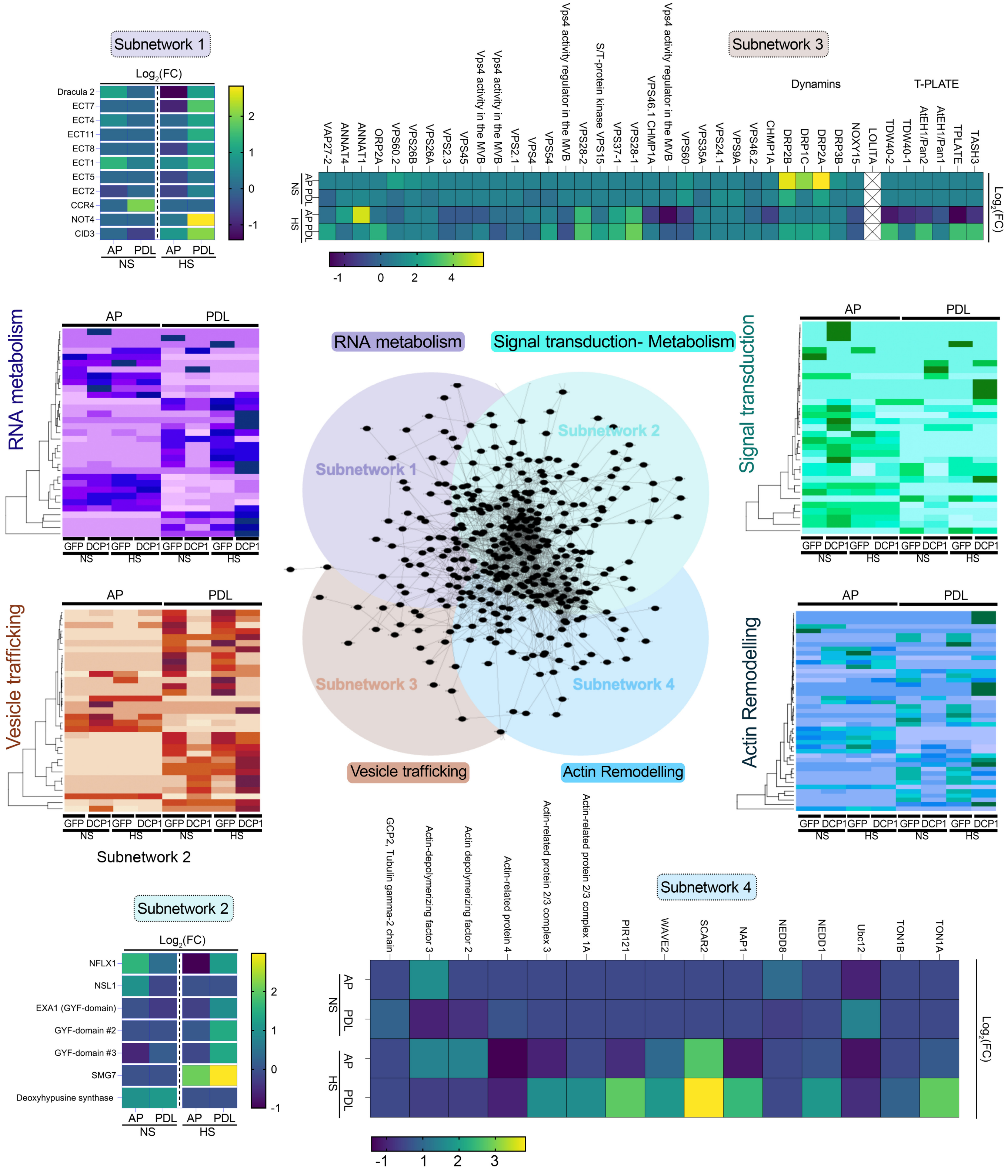

Figure EV5.

© 2023 The Authors

◀ **Figure EV5. Structure and density of the four interconnected networks.**

Subcluster analyses of APEAL reveal four interconnected subnetworks (center). The four networks were as follows: proteins related to RNA metabolism; signal attenuation, translation and metabolism; vesicle trafficking and actin remodeling. Heatmaps depict the abundance of selected proteins from the four subnetworks. Notably, the AP step in the “purple” heatmap did not lead to the identification of RNA metabolism proteins. In Appendix text, we summarize interesting hits from each network.

Source data are available online for this figure.

© 2023 The Authors
