# Supplementary material for: An actin remodeling role for Arabidopsis processing bodies revealed by their proximity interactome
Source: EMBO J. 2023 Feb 6;42(9):e111885. doi: 10.15252/embj.2022111885 (PMC10152145; doi:10.15252/embj.2022111885)
Supplement: Supplementary file 14 [file EMBJ-42-e111885-s002.pdf]

THE EMBO JOURNAL

An actin remodeling role for Arabidopsis processing bodies revealed by their proximity interactome

Chen Liu, Andriani Mentzelopoulou, Amna Muhammad, Andriy Volkov, Dolf Weijers, Emilio Gutierrez-Beltran, Panagiotis N. Moschou

DOI: 10.15252/embj.2022111885

Corresponding author: Panagiotis Moschou (Panagiotis.moschou@uoc.gr)

Review Timeline:

Submission Date:	15th Jun 22
Editorial Decision:	1st Aug 22
Revision Received:	30th Oct 22
Editorial Decision:	22nd Dec 22
Revision Received:	10th Jan 23
Accepted:	18th Jan 23

Editor: Ieva Gailite

Transaction Report:

(Note: With the exception of the correction of typographical or spelling errors that could be a source of ambiguity, letters and reports are not edited. Depending on transfer agreements, referee reports obtained elsewhere may or may not be included in this compilation. Referee reports are anonymous unless the Referee chooses to sign their reports.)

Thank you for submitting your manuscript for consideration by the EMBO Journal. We have now received comments from three reviewers, which are included below for your information.

As you will see from the reports, in particular reviewers #1 and #3 find the proposed role of DCP1 in regulation of actin dynamics and cell shape of interest. However, they also find that substantial additional evidence would be needed to convincingly support the proposed mechanism. All reviewers also find that the various parts of the manuscript are rather disjointed and do not form a cohesive story, and reviewer #2 with expertise in LLPS finds the link between DCP1 phase separation and its ability to regulate actin nucleation unclear. Additionally, reviewers #1 and #3 find that further support for the polar behaviour of DCP1 and its function in actin nucleation is needed.

If you find that you are able to address the main issues raised by the reviewers, I would be happy to consider a revised version of the manuscript. I think it would be helpful to discuss the revision in more detail via email or phone/videoconferencing - please let me know which option you prefer. I should also add that it is The EMBO Journal policy to allow only a single major round of revision and that it is therefore important to resolve the main concerns at this stage.

We generally allow three months as standard revision time. As a matter of policy, competing manuscripts published during this period will not negatively impact on our assessment of the conceptual advance presented by your study. However, please contact me as soon as possible upon publication of any related work to discuss the appropriate course of action. Should you foresee a problem in meeting this three-month deadline, please let us know in advance and we may be able to grant an extension.

When preparing your letter of response to the referees' comments, please bear in mind that this will form part of the Review Process File and will therefore be available online to the community. For more details on our Transparent Editorial Process, please visit our website: https://www.embopress.org/page/journal/14602075/authorguide#transparentprocess. Please also see the attached instructions for further guidelines on preparation of the revised manuscript.

Please feel free to contact me if you have any further questions regarding the revision. Thank you for the opportunity to consider your work for publication. I look forward to discussing your revision.

Referee #1:

The manuscript by Liu et al. described the localization of DCP1 on both p-bodies and the edge/vertex regions of the plasma membrane. The connection of DCP1 with plasma membrane was inferred initially via proximity labeling of DCP1 interaction proteins, experimentally validated by multiple high-resolution microscopic observations and quantitative assessments. The authors claimed that the membrane localization of DCP1 is of biological relevance by genetic analyses to show an association of membrane-localized DCP1 with cell shape determination in Arabidopsis roots. Overall the observation of DCP1 with the plasma membrane is new in plants. The evidence supporting the DCP1-SCAR-WAVE in determining the actin nucleation and subsequently leading to the anisotropic cell shape is intriguing. The authors clearly spent considerable effort. However, this study could benefit from a more focused presentation of the key results and tuning down conclusions not supported by the data

presented. Major concerns include:

1. The examination of the LLPS nature of DCP1-containing puncta in plant cells shown in Supplementary Figs 1 and 2 are remotely relevant to the central theme of this study and does not add new information to the current understanding of p-bodies in plants. Removing them from the manuscript should not impact the integrity of this present study.
2. For TurboID, it wasn't clear whether the log2FC>1 includes those with FC<1/2? According to Supplementary file 3, those with reduced peptides identified in DCP1 lines than in GFP lines were also included, but these proteins should not be classified as DCP1-interacting proteins, especially since the results will impact the network analyses shown in Fig. 2, and be used to infer the plasma membrane association and actin remodeling (subnetworks 3 and 4). Details for data analyses/filtering and the color codes in supplementary files should be better elaborated to facilitate understanding and evaluation of the data quality.
3. TIRFM results in Fig. 3 were representative images from relatively small numbers of biological/technical replicates. Based on the selected images and data analyses, the results presented do not firmly support the conclusion of increasing edge/vertex association from zone 1 to zone 3 (zone 1 and 2 images have many "edge-associated" DCP1 signals, not reflected by the model cells in Fig. 3c). The relevance of p-body sizes in Fig. 3c was unclear. The y-axis "relative signal partition PM/cytosol" unit was unclear. Does it mean the PM signals are 10-60 times higher than those in the cytosol? The polar behavior of DCP1 in Fig. 3d was also less than obvious. Also, the authors claimed there is "competition" between PB and plasma membranes, and PBs undergo fissions on the membrane interface. The authors should consider the alternative possibility of independent targeting of DCP1 to PBs and the plasma membrane, especially since DCP1 seems to be the only one detected on the plasma membrane. The co-localization data of DCP1 and PAT was not entirely unequivocal (Fig. 4b). Was the "fission at plasma membrane" more of a hypothetical inference from the animal PBs fission on ER as referenced (Lee 2020)?
4. Fig. 4 includes results showing mixed information of increasing association of DCP1 with edges in 2D that was shown in 2D in Fig. 3, as well as the lack of co-localization of DCP1 and DCP2 on the plasma membrane. The latter was used to infer that the membrane-associated DCP1 does not possess decapping activities. It was unclear why decapping is relevant here in this study. Also, is undetectable PLA for DCP1 and DCP2 equal to no decapping activities on the plasma membrane?
5. Compared to Fig. 3 and 4, results showing the DCP1-SCAR-WAVE edge/vertex localization and the actin nucleation were supported with better evidence (Fig. 5, 6, and 7). High-quality images and improved organization/elaboration of the results will further enhance readability.
6. The connection of DCP1-SCAR-WAVE-actin nucleation and the anisotropy is an interesting observation (Fig. 8) and flows nicely after Figs 5-7. Genetic data seem to support the cause-effect relationship between DCP1-SCAR-WAVE-actin nucleation with anisotropy growth. However, whether the increased isotropic growth in dcp1 or higher order scar mutants results from reduced cell elongation (therefore more isotropic) could be investigated or at least discussed.

Minor points:
1. Figure legends for Supplementary Fig. 3b and 3c were reversed?
2. Supplementary File 4, the headers "common, gene name, description" are missing for GFP-DCP2, AP22. Literature for the published proteomics datasets should be referenced (lines 138-140)
3. Fig. 2a is not particularly informative or helpful. It's also unclear whether the heatmap shows the average results of the replicates?
4. The yellow arrowhead in Fig. 3d indicates?

Referee #2:

In this work Liu et al use proximity proteomics and imaging approaches to study the composition of processing bodies in plant cells. They find that PBs are interacting with the plasma membrane and actin cortex components. Based on genetic perturbations experiments they conclude that the PB component DCP1 controls polarized actin nucleation at cell corners, which is important for anisotropic cell growth.
Overall, I found the manuscript quite difficult to read. The manuscript touches on multiple different stories: phase separation properties of PBs, the protein interactome of PBs during heat stress, membrane interactions of PBs, actin regulation of PBs and finally polarized cell growth. Even though the authors deployed a tour de force of many different methods to address the different stories, I find that the experimental support for many of the main conclusions remains shallow. For example, I don't see a clear connection between phase separation of PB and the membrane localization of DCP1. I do not understand what the material properties of PB have to do with polarized actin nucleation. I think, it may help to split this paper into smaller stories, in which some of the hypothesis can be explored in more depth and more rigorously.

Referee #3:

This study embarks on a proteomics and cell biological investigation of the formation an dynamics of a key molecular condensate, the processing body (PB). Their APEAL pipeline identifies four subnetworks of interacting proteins (Fig. 2 and Suppl. Fig. 7) involved in RNA metabolism; defense regulation and metabolism; membrane trafficking; and cytoskeletal function of both actin and microtubules. The paper also includes a wealth of cell biological characterization on DCP1 association with one of its putative interactors, the SCAR/WAVE nucleation promoting factor. The strongest conclusion that can be made is that DCP1

appears to be recruited by the SCAR/WAVE complex to cell edges and vertices during development, but this does not appear to include other components of the PB, like DCP2. Further, the ability of DCP1-SCAR/WAVE complex to recruit or stabilize actin at cell edges/vertices appears to depend on the phosphorylation status of DCP1 at S237. As such, the general layout of the experiments and conclusions drawn seem overambitious and perhaps misleading. The title linking PB to a module for instructing actin-driven directional growth is not well supported by the experiments. I suggest they choose something more accurate linking DCP1 specifically to SCAR/WAVE, and potentially actin remodelling and frame their experimental description to fit this context.

Most experiments are expertly conducted and critically analyzed, however, a few areas might be expanded or clarified.

1) To better understand the network of protein interactions, and validate association with key cytoskeletal players in subnetwork 4, I suggest they conduct the APEAL analysis with DCP1-S237D and DCP1-S237A.
2) Given that they want to link DCP1 to actin filament nucleation at cell edges/vertices through SCAR/WAVE, I suggest that they do more to include fluorescent ARP2/3 complex subunits in their colocalization and SE-FRET experiments (Fig. 5). The reason being that this is indeed the nucleator of actin filaments, not SCAR/WAVE. Indeed, SCAR/WAVE might be localized to cell edges/vertices without activating ARP2/3 complex. Indeed, the data with arpc5 (Suppl. Fig. 10d) seem to support this conclusion
3) Finally, rather than using Cytochalasin D, a more appropriate inhibitor to disrupt actin nucleation would be CK-666, a small molecule inhibitor of ARP2/3 complex. At a minimum, they should use the polymerization inhibitor latrunculin A or B, in addition to CytD which stabilizes actin filaments through binding barbed ends.
4) The data in Supplementary Fig. 11 on actin localization in dcp1 and other mutants are central to linking the DCP1-SCAR/WAVE axis to actin-based function and perhaps cell growth and deserve to be in the main text. Similarly the MT-depleted cell vertices that are enriched for actin, should be expanded to include analysis of DCP1 and/or SCAR/WAVE. (I am not worried so much about pleiotropic effects if the authors were to use acute treatments with APM or oryzalin). More comparison and discussion of the similarities between these findings and those for the apex of trichome branches where a similar microtubule-dependent ARP2/3 confinement zone has been demonstrated by Yanagisawa et al. 2018 is certainly merited.

Minor comments:
1) It would be important to have quantitative assessment of the ability of tagged DCP1 proteins (e.g. GFP-tagged, TurboID-6XHis-Flag, etc.) to partially of fully complement dcp1 mutants.
2) The assay described in Fig. 7d does not demonstrate actin nucleation as claimed, just recovery of the LifeAct-mCherry which could occur through a myriad of mechanisms.
3) Protein condensate dynamics (stable, fusion, fission) shown in Fig. 3b, it needs to be stated what was the developmental stage of epidermal cells examined? Did they do identical analyses in all three developmental regions?
4) The legend for Fig. 4b is mostly methods for PLA assay. This should be moved to appropriate section of manuscript.
5) Ideally, the authors would test the association of DCP1 with SCAR/WAVE in vitro to promote the complexes' ability to activate ARP2/3 nucleation, as demonstrated by others for SCAR2 (D. Basu et al., 2005. Plant Cell 17: 502-24).
6) The descriptions for subparts b and c in Fig. 6 seem to be callouts to wrong experiments. Please correct.
7) It's not clear how they measure relative intensity of actin at cell edges in Fig. 5, but the representative image for DCP1-S237D doesn't appear to have reduced abundance compared with DCP1-WT. Perhaps that is because these are confocal projections?

Revision for EMBOJ-2022-111885

We want to thank the reviewers for their constructive criticism and useful comments on our manuscript. We have performed many additional experiments and thoroughly revised the manuscript by including new figures, as well as by making extensive textual amendments. The additional experiments resulting in new or revised sets of data are summarized in the table below:

Additional experiments or analyses	Action taken
Reviewer 1	
LLPS nature of DCP1	Data removed as requested
log2FC>1 versus those with FC<1/2	Source Files 1-4, Source File 2 for enriched and 1 without filtering
Edge/vertex association and TIRFM data	Fig 4A and several other places in the manuscript. Additional 3D rendering along the proximodistal axis (on the right). Appendix Fig S1 further includes the VCS subunit, to improve readability.
Competition between PBs and PM	Appendix Fig S1A and C. CHX and photoconversion experiments.
Increased isotropic growth in dcp1 or higher order scar mutants results from reduced cell elongation (therefore more isotropic) could be investigated or at least discussed.	Fig 8B and Appendix Fig S9, new experiments
Reviewer 3	
Include fluorescent ARP2/3 complex subunits in colocalization and FRET experiments	Appendix Fig S5
a more appropriate inhibitor to disrupt actin nucleation would be CK-666	Appendix Fig S5 and S6
MT-depleted cell vertices that are enriched for actin, should be expanded to include analysis of DCP1 and/or SCAR/WAVE.	Appendix S7
Quantitative assessment of the ability of tagged DCP1 proteins to partially of fully complement dcp1 mutants.	Appendix S1
Improved measurements for DCP1-S237D actin	Fig 6A, chart

Below, please find our detailed point-by-point responses to the reviewers' comments.

Referee #1:

The manuscript by Liu et al. described the localization of DCP1 on both p-bodies and the edge/vertex regions of the plasma membrane. The connection of DCP1 with plasma membrane was inferred initially via proximity labeling of DCP1 interaction proteins, experimentally validated by multiple high-resolution microscopic observations and quantitative assessments. The authors claimed that the membrane localization of DCP1 is of biological relevance by genetic analyses to show an association of membrane-localized DCP1 with cell shape determination in Arabidopsis roots. Overall the observation of DCP1 with the plasma membrane is new in plants. The evidence supporting the DCP1-SCAR-WAVE in determining the actin nucleation and subsequently leading to the anisotropic cell shape is intriguing. The authors clearly spent considerable effort. However, this study could benefit from a more focused presentation of the key results and tuning down conclusions not supported by the data presented.

We have now toned down the conclusions not well supported by the data or have removed them, according to the reviewer's request:

1) almost all references to material properties have been removed. We keep parts of the discussion with relevance to the material properties, lines 447-459.

2) We focus on the following: a) the PBs protein composition and why the TurboID should be used for capturing condensates' proteomes. Other approaches, such for example FAPS (Hubstenberger *et al*, 2017) or gradient centrifugation may enrich larger condensates or the core lacking the liquified shell, which has "client" proteins as the one we define here that belong to the SCAR–WAVE complex (and other complexes). Hence, TurboID approaches in plants will complement or even surpass current approaches, providing "proxitomes" with important new information; b) the link of SCAR/WAVE to ARP2/3 through DCP1, and the effects on actin nucleation. We further support these links, using drugs and genetic tools. Below, we provide more details.

Major concerns include:

1. The examination of the LLPS nature of DCP1-containing puncta in plant cells shown in Supplementary Figs 1 and 2 are remotely relevant to the central theme of this study and does not add new information to the current understanding of p-bodies in plants. Removing them from the manuscript should not impact the integrity of this present study.

We agree with the reviewer that the material properties of PBs (DCP1-positive puncta in the cytoplasm) are a marginally relevant theme here. We thus have now removed these datasets.

2. For TurboID, it wasn't clear whether the log2FC>1 includes those with FC<1/2? According to Supplementary file 3, those with reduced peptides identified in DCP1 lines than in GFP lines were also included, but these proteins should not be classified as DCP1-interacting proteins, especially since the results will impact the network analyses shown in Fig. 2, and be used to infer the plasma membrane association and actin remodeling (subnetworks 3 and 4). Details for data analyses/filtering and the color codes in supplementary files should be better elaborated to facilitate understanding and evaluation of the data quality.

We have changed the presentation accordingly. FC<1/2 peptides were not included to infer the "proxitome". More specifically, in Supplementary file 3, we included all the peptides captured in all samples tested via TurboID. To avoid confusion and misinterpretation of this important dataset, we have now created two separate lists; Source File 1, containing all the genes captured in the APEAL dataset with first-row filtering to facilitate data searching; Source File 2 containing genes enriched in PBs in NS conditions (AP and PDL with a [Log2(FC)⩾1]) and genes enriched in PBs in HS conditions (AP and PDL with a (Log2(FC)⩾1), respectively. Although all our data have been submitted to public depositories (i.e., PRIDE: 1-20221024-52704 /nfs/pride/drop/pride-drop-003/andriani_mentzelopoulou_20221024_163053), we still think that we should provide the "noisy" part of our proteome through unmodified datasets, as the TurboID approaches in plants are in their infancy, and thus, our raw data can help to develop them further.

3. TIRFM results in Fig. 3 were representative images from relatively small numbers of biological/technical replicates. Based on the selected images and data analyses, the results presented do not firmly support the conclusion of increasing edge/vertex association from zone 1 to zone 3 (zone 1 and 2 images have many "edge-associated" DCP1 signals, not reflected by the model cells in Fig. 3c).

We agree with the reviewer that additional regions should ideally have been tested with TIRFM, but the TIRFM inherent properties do not allow the examination of epidermal cell regions surrounded by root cap cells (i.e., zone 1). TIRFM in other regions (1 and 2) would examine the surface of root cap cells, which have completely different properties than epidermal cells and they are not relevant to our study. For better examination though, we have used confocal high-resolution imaging at a super-resolution setting described in materials and methods, as edge association is not visible by TIRFM. We should also note, that TIRFM was used to provide evidence that PBs indeed attach to the PM, and not for other reasons (TIRFM can only "see"

proteins or complexes at the PM plain due to the shallow illumination depth and its exponential signal decay). This is clarified in more detail in the text (Lines 174-184), along with technicalities considering the method. We have obtained a very large number of datasets with TIRFM, as N=3 x 8 cells (PMs; as explained in the caption), with 500 images each to ensure that the PBs can attach to the PM.

We provide more data showing the localization of DCP1 in much more detail (Figs 3C and 4A, B and elsewhere, e.g., in Figs 6B, 5A, and 7C). Indeed, the robustness of edge localization was not very high, as also stated in our text (Line 482-497: *"Perhaps the most puzzling contradiction in our data is the variable edge/vertex decoration by SCAR–WAVE or DCP1. The lack of robustness of this process may relate to a seemingly stochastic condensation of SCAR–WAVE–DCP1 at regions closer to the QC, which can bring about local asymmetries in anisotropy at the cellular level. We accordingly show that cells with edges/vertices well defined by this complex follow a highly predictable anisotropic growth pattern, while cells with less determined SCAR–WAVE–DCP1 vertices have more diffusible growth patterns."*). What was clear from our data though was that zones 1-4 differ in the probability of having stable DCP1 on edge, which we quantify in detail and provide better images (Fig 4A, plot profile and circular plots). Furthermore, this localization should not be confused with the transient localization of DCP1 at the edge of the cell plate, which can mix the results from region 1 (e.g., Fig 3C and in the clearer image in Appendix Fig S4A). The corresponding model has been removed, as perhaps is confusing.

4. The relevance of p-body sizes in Fig. 3c was unclear.

Thank you very much for raising this issue. In our study, we focused on cells of the same lineage, the root epidermis to obtain coherent results. Innermost tissues also showed similar localizations but would be more challenging to link TIRFM-confocal imaging-isoxaben effects in this work, as we focused on isoxaben concentrations mainly affecting the epidermis. The sizes of PBs decrease with the developmental stage in the epidermis, suggesting that PBs may lose material to the PM or get denser due to processes such as condensate aging (e.g., through Ostwald ripening or other processes associated with an increase in viscosity; (Jawerth *et al*, 2020)). This is consistent with our model in which we suggest that SCAR/WAVE sequesters DCP1 from PBs (now shown also using EosFP-DCP1 photoconversion: Appendix Fig S1C). Yet, this is not true for distal stem cell lineages that do not undergo cell division, e.g., the root cap cells. We would not like to include analyses for root cap cells, as it is beyond our scope here (as the focus is on the interface at the PM), and we plan to address the fate of PBs during cell division or in this distal stem cell type in detail in future work, as we believe it represents an excellent *in vivo* model for studying condensates' aging. We thus removed these datasets from the current version.

5. The y-axis "relative signal partition PM/cytosol" unit was unclear. Does it mean the PM signals are 10-60 times higher than those in the cytosol? The polar behavior of DCP1 in Fig. 3d was also less than obvious.

We thank the reviewer for bringing these issues to our attention. The integrated overall PM signal intensity was indeed much higher than the average intensity in the bulk cytoplasm and not in PBs (areas with or without PBs; the latter would give zero signal). Furthermore, in the caption of Appendix S1, we include the statement "As a cautionary note, the cytoplasmic pool of DCP1 may not be in a dilute phase but could form diffraction-limited PBs." We thus believe that measuring the bulk cytoplasm might lead to more accurate results.

The apicobasal polar behavior of DCP1 is now shown in more detail in the following images: Fig. 3C, right panel, 4A and B, and 6B (with FM4-64 counterstaining). Note that in 3C we include additional promoters, to make sure the effect of polarity and localization of DCP1 is not due to changes in expression levels. Overall, the polarity index (around 2 in 3C, and with high variation) shows that DCP1 apicobasal accumulation is not exclusive.

6. Also, the authors claimed there is "competition" between PB and plasma membranes, and PBs undergo fissions on the membrane interface. The authors should consider the alternative possibility of independent targeting of DCP1 to PBs and the plasma membrane, especially since DCP1 seems to be the only one detected on the plasma membrane. The co-localization data of DCP1 and PAT was not entirely unequivocal (Fig. 4b). Was the "fission at plasma membrane" more of a hypothetical inference from the animal PBs fission on ER as referenced (Lee 2020)?

We thank the reviewer for bringing this issue to our attention. Please note, that CHX induced the rapid dissolution of PBs and the re-localization of DCP1 at the PM, implying that the same molecules localized there (as translation is blocked) (Appendix Fig S1B). Yet, as suggested by the reviewer, we now add a cautionary note that indeed some of the molecules of DCP1 may directly be targeted there toning down our conclusions (Lines 198-204). The levels of DCP1 at the cell plate are also low and DCP1 only appears at the cell plate transiently during its fusion stage with the cell PM. This result speaks against a general targeting of DCP1 at the plasma membrane but implies the binding of DCP1 at $PI4,5P_2$ lipids (the cell plate's edge is rich in this lipid before fusion). We have data showing that DCP1 can indeed bind this lipid *in vitro* (Appendix 1 in this file-Response to reviewers). Yet, we feel that this dataset is beyond the scope of this work. We further managed to establish photoconvertible stable lines with green-to-red shifted EosFP-DCP1, which strengthen our suggestion of direct transfer of DCP1 molecules from PBs to the PM (Appendix Fig S1C and caption). The fission at the plasma membrane relates to the observation of DCP1-positive puncta split at the PM, suggesting that there is a PM-localized mechanism that directs the removal of DCP1 from the PBs (Figs 4C and the diffusion in the 3B kymograph). We believe that this mechanism entails the function of SCAR/WAVE, although further work is needed to address the exact mechanism of the tug-of-war between SCAR/WAVE and PBs for DCP1. As this might be confusing, distracting, and speculative as to which is the underlying mechanism, we have accordingly discussed in detail this point (Line 222-239). Indeed, the work from Lee et al., (2020) was inspirational to this end, although the mechanism in this work by which PBs are split is unclear.

7. Fig. 4 includes results showing mixed information of increasing association of DCP1 with edges in 2D that was shown in 2D in Fig. 3, as well as the lack of co-localization of DCP1 and DCP2 on the plasma membrane. The latter was used to infer that the membrane-associated DCP1 does not possess decapping activities. It was unclear why decapping is relevant here in this study. Also, is undetectable PLA for DCP1 and DCP2 equal to no decapping activities on the plasma membrane?

We thank the reviewer for spotting this omission which would otherwise have led to reasonable confusion; we have modified the text from lines 161-166, and we believe that it fits nicely with the proposed effects of only DCP1 to the observed phenotypes and the linkage to actin. Other decapping mutants did not show the same defects (e.g., Fig 8). To this end, we improved our introduction, discussing the major components of the decapping machinery. The PLA and FRET together imply that a minimal and competent decapping complex (i.e., DCP1-DCP2) is not formed at the PM. This DCP1-DCP2 linkage is an indirect criterion for decapping activity and shows that DCP1 likely is most of the time alone on the PM (without components of PBs, especially DCP2; we provide more and improved data and analyses in this direction in the revised manuscript, including the VCS component, Appendix Fig. S1A). When the SCAR/WAVE-DCP1 condensate is formed at the edge/veretx, DCP2, PAT1 and other proteins of PBs are not there anymore. Our data likely suggest that PBs split on the PM and DCP1 has an affinity for the SCAR/WAVE and PM and stays on it (DCP1 has a pleckstrin homology domain [PH domain], see also above response). The decapping activity is exerted by DCP2, and this interaction has been used as a criterion before in animal cells (Lin *et al*, 2006; Luo *et al*, 2021; Tibble *et al*, 2021). The observed cell polarity defects (e.g., Fig. 8) could have been brought about by mRNA decapping which can alter the proteomic landscape. We refute this possibility by using the relevant decapping mutants *dcp2, dcp5,* and *xrn4* mutants, and we

streamlined our manuscript along these lines (e.g., session: DCP1 interfaces with plasma membrane domains at the cellular face independently of its role in decapping).

8. Compared to Fig. 3 and 4, results showing the DCP1-SCAR-WAVE edge/vertex localization and the actin nucleation were supported with better evidence (Fig. 5, 6, and 7). High-quality images and improved organization/elaboration of the results will further enhance readability.

We agree with the reviewer and now we present an improved Fig. 4 version; some Fig. 3 data have not been included as they produced redundant or confusing information (see above comments). In the previous version, many images were 3D projections that did not reproduce what we could see under the microscope, while, at the same time, hiding information. We have now selected Z-decompositions in Fig. 4A, right panel, as a criterion of edge vs. vertex localization.

9. The connection of DCP1-SCAR-WAVE-actin nucleation and the anisotropy is an interesting observation (Fig. 8) and flows nicely after Figs 5-7. Genetic data seem to support the cause-effect relationship between DCP1-SCAR-WAVE-actin nucleation with anisotropy growth. However, whether the increased isotropic growth in dcp1 or higher order scar mutants results from reduced cell elongation (therefore more isotropic) could be investigated or at least discussed.

We thank the reviewer for highlighting what we should highlight further. We thus have addressed this point and built our story around it; we now provide more detailed information. A relevant chart (lower right) is the one in Appendix Fig. S9B in which we show that cell anisotropic expansion is reduced (cell elongation reduced). We discuss this point in more detail as the information was rather cryptic and could have been easily lost and included also the *scar1234* and *dcp1* mutants in our analyses. We have expanded the relevant analyses though to make sure this key message, which is well-supported by genetics, is efficiently conveyed. Our results rather show that *scar1234* and *dcp1* can also show slightly reduced expansion (Appendix Fig S9B), which should in principle lead to reduced ISX sensitivity (lines 421-437)- that is not though the case, as was also observed for the S237D mutant. We have also changed the analyses here to improve robustness (see the line chart in the corresponding figure, Appendix Fig S9B along with the improved statistics).

Minor points:

1. Figure legends for Supplementary Fig. 3b and 3c were reversed?

Yes, we apologize for that. Now fixed (Figure EV1).

2.Supplementary File 4, the headers "common, gene name, description" are missing for GFP-DCP2, AP22. Literature for the published proteomics datasets should be referenced (lines 138-140)

We apologize for this serious omission of proper citations. The citations were in the supplemental info but were too cryptic. We now provide the missing information in lines 133-140 and Source File 6.

3. Fig. 2a is not particularly informative or helpful. It's also unclear whether the heatmap shows the average results of the replicates?

We fully agree. This panel has now been removed.

4. The yellow arrowhead in Fig. 3d indicates?

The cell plate. Now added in the fig. caption (3C).

Referee #2:

In this work Liu et al use proximity proteomics and imaging approaches to study the composition of processing bodies in plant cells. They find that PBs are interacting with the plasma membrane and actin cortex components. Based on genetic perturbations experiments they conclude that the PB component DCP1 controls polarized actin nucleation at cell corners, which is important for anisotropic cell growth. Overall, I found the manuscript quite difficult to read. The

manuscript touches on multiple different stories: phase separation properties of PBs, the protein interactome of PBs during heat stress, membrane interactions of PBs, actin regulation of PBs and finally polarized cell growth. Even though the authors deployed a tour de force of many different methods to address the different stories, I find that the experimental support for many of the main conclusions remains shallow.

We thank the reviewer for the suggestion to provide a more focused story. Now we study the proposed mechanisms in detail. According to the suggestions from this and other reviewers, we have simplified our story and highlighted two areas: 1. The plant PBs proximtome ± HS. The HS was used here as a tool rather than as a biological readout, for example, to show that DCP1/SCAR nucleate more extensively which can explain the APEAL results and the increased colocalizations we observed under HS (also the increased formation of PBs, and the increased interactions among the core PB components; Fig 1B). 2. The discovery of membrane interactions and actin regulation (HS treatments which promote interactions with SCAR/WAVE, phosphovariants with reduced or increased nucleation activities due to differential interaction with the SCAR/WAVE. We provide critical data in support of this nucleation, by further discovering links to Arp2/3 and DCP1.

For example, I don't see a clear connection between phase separation of PB and the membrane localization of DCP1. I do not understand what the material properties of PB have to do with polarized actin nucleation. I think, it may help to split this paper into smaller stories, in which some of the hypothesis can be explored in more depth and more rigorously.

We reinforce our analyses using photoconvertible DCP1 molecules, which clearly suggest that the inclusion of DCP1 in PBs counteracts its localization to the PM. Yet, as these data may need further support with *in vitro* experiments and perhaps too much space to address further material properties, as suggested, we toned down our conclusions and have removed the relatively few links to material properties.

Referee #3:

This study embarks on a proteomics and cell biological investigation of the formation an dynamics of a key molecular condensate, the processing body (PB). Their APEAL pipeline identifies four subnetworks of interacting proteins (Fig. 2 and Suppl. Fig. 7) involved in RNA metabolism; defense regulation and metabolism; membrane trafficking; and cytoskeletal function of both actin and microtubules. The paper also includes a wealth of cell biological characterization on DCP1 association with one of its putative interactors, the SCAR/WAVE nucleation promoting factor. The strongest conclusion that can be made is that DCP1 appears to be recruited by the SCAR/WAVE complex to cell edges and vertices during development, but this does not appear to include other components of the PB, like DCP2. Further, the ability of DCP1-SCAR/WAVE complex to recruit or stabilize actin at cell edges/vertices appears to depend on the phosphorylation status of DCP1 at S237. As such, the general layout of the experiments and conclusions drawn seem overambitious and perhaps misleading. The title linking PB to a module for instructing actin-driven directional growth is not well supported by the experiments. I suggest they choose something more accurate linking DCP1 specifically to SCAR/WAVE, and potentially actin remodelling and frame their experimental description to fit this context.

We agree and we have chosen something more relevant, taking into consideration space restrictions (100 characters including spaces), such as "**The "Proxitome" of Arabidopsis Processing Bodies Reveals a Membrane Interface Remodeling Actin**". Accordingly, we have toned down many parts of our work and framed our experimental description along these lines.

Most experiments are expertly conducted and critically analyzed, however, a few areas might be expanded or clarified.

1) To better understand the network of protein interactions, and validate association with key cytoskeletal players in subnetwork 4, I suggest they conduct the APEAL analysis with DCP1-S237D and DCP1-S237A.

We thank the reviewer, and we are on the same page on this. Yet, we feel that re-doing the APEAL for the DCP1 variants is beyond the scope of this work or what could be achieved in a reasonable timeframe. The DCP1 variants have similar localization with WT DCP1 in most tissues and this endeavor would require extensive efforts which likely fall beyond the major goals of this work. The APEAL should be conducted from very few cells with different localization of the variants which is not easy (please see Fig. 6B for the spatial nature of changes in localization which confirms that they are in a very narrow developmental zone). As this is an excellent suggestion, we plan to conduct this work in the future, setting single-cell proteomics and/or the less risky tissue-specific proteomics. Please also note that SCAR2, the major interacting protein from the SCAR/WAVE with DCP1, had reduced/increased FRET efficiency and colocalization with the phosphomimetic or phosphomutant, respectively, as shown in Fig. 7A-C, which confirms the differences of association. In addition, we now include analyses of SCAR2 in *dcp1-3* mutants which confirms the mutual potentiation of SCAR/WAVE-DCP1 localization, which could impart actin remodeling, as suggested (Fig. 7C).

2) Given that they want to link DCP1 to actin filament nucleation at cell edges/vertices through SCAR/WAVE, I suggest that they do more to include fluorescent ARP2/3 complex subunits in their colocalization and SE-FRET experiments (Fig. 5). The reason being that this is indeed the nucleator of actin filaments, not SCAR/WAVE. Indeed, SCAR/WAVE might be localized to cell edges/vertices without activating ARP2/3 complex. Indeed, the data with arpc5 (Suppl. Fig. 10d) seem to support this conclusion

We fully agree with this suggestion, and we have now conducted these experiments. We cloned ARPC5 and generated new lines co-expressing DCP1 (and different phosphovariants) with ARPC5 fluorescently tagged. Although the exact localization of the active ARP2/3 is still a lingering question (Zhang *et al*, 2013), and SCAR/WAVE may execute independent functions (Ali *et al*, 2020), we were able to show that DCP1and ARPC5 partially interact at edges/vertices. We though provide evidence that ARPC5 shows reduced specificity for vertices when compared to SCAR/WAVE or DCP1(Appendix Figs S5A and S8). We discuss these points in more detail, improving our APEAL subnetwork 4 analyses on SCAR/WAVE components in Lines 323-337 and Source File 7. Together with the suggested experiment in point 3, our data, as the reviewer suggested indicate a link to ARP2/3.

3) Finally, rather than using Cytochalasin D, a more appropriate inhibitor to disrupt actin nucleation would be CK-666, a small molecule inhibitor of ARP2/3 complex. At a minimum, they should use the polymerization inhibitor latrunculin A or B, in addition to CytD which stabilizes actin filaments through binding barbed ends.

We fully agree with the reviewer here and this is a very relevant suggestion. At the beginning of our revision, we thought that SCAR/WAVE may function independently of ARP2/3. We used Lat B which produced similar results and is now included in the figures (Appendix Fig S6), and we expanded our screening using CK-666 as a specific ARP2/3 inhibitor (Appendix Fig S5C). The activation of the ARP2/3 complex through the SCAR/WAVE is required to induce cellular actin nucleation, although other pathways can function to this end (Ali *et al.*, 2020). CK-666 (40 to 200 μM) stabilizes the inactive state of the ARP2/3 complex, blocking the movement of the ARP2/3 subunits into the activated filament-like conformation (Nolen *et al*, 2009). Intriguingly, SCAR2 was the most prominent interactor for DCP1 among the SCAR/WAVE components (Fig EV5, subnetwork 4 and Source File 7). Together with the *in-situ* localization of ARP2/3 and FRET analyses (Appendix Fig S5 and Fig S8), our new data support the reviewer's suggestion that ARP2/3 is downstream of SCAR/WAVE in this pathway. Hence, SCAR-WAVE recruits DCP1 which potentiates ARP2/3 localization.

4) The data in Supplementary Fig. 11 on actin localization in dcp1 and other mutants are central to linking the DCP1-SCAR/WAVE axis to actin-based function and perhaps cell growth and deserve to be in the main text.

We agree with the reviewer, and we moved the data into the main figure, now the data is presented in Fig 6C.

Similarly the MT-depleted cell vertices that are enriched for actin, should be expanded to include analysis of DCP1 and/or SCAR/WAVE. (I am not worried so much about pleiotropic effects if the authors were to use acute treatments with APM or oryzalin). More comparison and discussion of the similarities between these findings and those for the apex of trichome branches where a similar microtubule-dependent ARP2/3 confinement zone has been demonstrated by Yanagisawa et al. 2018 is certainly merited.

We agree with the reviewer, and we have conducted these experiments, with the data presented in Appendix Fig S7. We stated the following in the text line 349-352: *"Intriguingly, in the apex of trichome branches, a similar MT-depletion zone confines ARP2–ARP3 and actin (Yanagisawa et al, 2018), implying that narrow membrane domains tend to accumulate these complexes. Whether DCP1 accumulates in the trichome apex remains to be shown."*

Minor comments:

1) It would be important to have quantitative assessment of the ability of tagged DCP1 proteins (e.g. GFP-tagged, TurboID-6XHis-Flag, etc.) to partially of fully complement dcp1 mutants.

The GFP-tagged lines (WT variant) fully complement the *dcp1-1* phenotype (Yu *et al*, 2019). The TurboID-6XHis-Flag was introduced into the *dcp1-3* background and can fully complement *dcp1-3* mutant. We now provide the corresponding comparisons of complementation. When DCP1 was driven by the RPS5a promoter (stem cell-specific promoter), we observed partial complementation, suggesting that DCP1 is important also in non-meristematic cells. We include this statement in Figure EV1, along with the corresponding quantifications (Fig. EV1A).

2) The assay described in Fig. 7d does not demonstrate actin nucleation as claimed, just recovery of the LifeAct-mCherry which could occur through a myriad of mechanisms.

We fully agree with this statement. In the current version, we have excluded datasets with relevance to FRAPs. As per the reviewer's suggestion, LifeAct-mCherry recovery or DCP1 molecules is not a suitable readout here as it could have occurred through multiple mechanisms.

3) Protein condensate dynamics (stable, fusion, fission) shown in Fig. 3b, it needs to be stated what was the developmental stage of epidermal cells examined? Did they do identical analyses in all three developmental regions?

We refer the reviewer to the response to point 3 raised by reviewer 1. We agree with the reviewer that additional regions should have been tested, but the TIRFM inherent properties do not allow the examination of regions surrounded by root cap cells, and where DCP1 is not found on the PM (and they lack edges). TIRFM would then examine the surface of root cap cells, which have completely different properties than epidermal cells. The region of interest, as defined in a is region 3. We now clarify this point in more detail in the figure legend and in the text, and we followed the dynamics of DCP1 further through high-resolution imaging.

4) The legend for Fig. 4b is mostly methods for PLA assay. This should be moved to appropriate section of manuscript.

Done, thanks; now moved to the appendix fig S2 caption.

5) Ideally, the authors would test the association of DCP1 with SCAR/WAVE in vitro to promote the complexes' ability to activate ARP2/3 nucleation, as demonstrated by others for SCAR2 (D. Basu et al., 2005. Plant Cell 17: 502-24).

We agree with the reviewer on this point, yet this *in vitro* assay is beyond the scope of this manuscript or what could be achieved in a reasonable and timely manner. Condensates are non-stoichiometric assemblies and therefore a significant amount of optimization would be required here. Furthermore, the exact components for recapitulating the *in vivo* condition

(SCAR/WAVE-ARP2/3-DCP1 and perhaps other accessory proteins), are currently unknown. For example, we refer the reviewer to the work of Rosen's group, in which this question was examined in detail in a paper showing that the condenser in this instance was the WASP (an IDR-rich protein). We believe that our condenser here is the DCP1, which counteracts the lack of WASP in plants. As of note, DCP2 in plants is lacking IDRs, unlike the situation in other systems, which could explain why DCP1 is an important scaffold of PBs in plants.

6) The descriptions for subparts b and c in Fig. 6 seem to be callouts to wrong experiments. Please correct.

Corrected, thanks.

7) It's not clear how they measure relative intensity of actin at cell edges in Fig. 5, but the representative image for DCP1-S237D doesn't appear to have reduced abundance compared with DCP1-WT. Perhaps that is because these are confocal projections?

We thank the reviewer for bringing to our attention this omission. The reviewer is right, and we now provide a better image for the WT, and we updated the corresponding statistical comparisons (Fig. 6A-C). The relative intensity was calculated by comparing the signal ratio between PM localized actin (or the actin at cell contours as we are not certain whether this pool of actin molecules is indeed attached to the PM) versus the one at the edge (at single spots on tricellular junctions). We now provide the description to the figure caption, that the signal is normalized against the overall signal at the PM.

APPENDIX:

Figure for reviewers removed

Hubstenberger A, Courel M, Benard M, Souquere S, Ernoult-Lange M, Chouaib R, Yi Z, Morlot JB, Munier A, Fradet M *et al* (2017) P-Body Purification Reveals the Condensation of Repressed mRNA Regulons. *Mol Cell* 68: 144-157 e145

Jawerth L, Fischer-Friedrich E, Saha S, Wang J, Franzmann T, Zhang X, Sachweh J, Ruer M, Ijavi M, Saha S *et al* (2020) Protein condensates as aging Maxwell fluids. *Science* 370: 1317-1323

Lin MD, Fan SJ, Hsu WS, Chou TB (2006) Drosophila decapping protein 1, dDcp1, is a component of the oskar mRNP complex and directs its posterior localization in the oocyte. *Dev Cell* 10: 601-613

Luo Y, Schofield JA, Na Z, Hann T, Simon MD, Slavoff SA (2021) Discovery of cellular substrates of human RNA-decapping enzyme DCP2 using a stapled bicyclic peptide inhibitor. *Cell Chem Biol* 28: 463-474.e467

Nolen BJ, Tomasevic N, Russell A, Pierce DW, Jia Z, McCormick CD, Hartman J, Sakowicz R, Pollard TD (2009) Characterization of two classes of small molecule inhibitors of Arp2/3 complex. *Nature* 460: 1031-1034

Snead WT, Hayden CC, Gadok AK, Zhao C, Lafer EM, Rangamani P, Stachowiak JC (2017) Membrane fission by protein crowding. *Proceedings of the National Academy of Sciences*: 201616199

Tibble RW, Depaix A, Kowalska J, Jemielity J, Gross JD (2021) Biomolecular condensates amplify mRNA decapping by biasing enzyme conformation. *Nat Chem Biol*

Yu X, Li B, Jang GJ, Jiang S, Jiang D, Jang JC, Wu SH, Shan L, He P (2019) Orchestration of Processing Body Dynamics and mRNA Decay in Arabidopsis Immunity. *Cell Rep* 28: 2194-2205 e2196

Zhang C, Mallery EL, Szymanski DB (2013) ARP2/3 localization in Arabidopsis leaf pavement cells: a diversity of intracellular pools and cytoskeletal interactions. *Front Plant Sci* 4: 238

Thank you for submitting a revised version of your manuscript. I sincerely apologise for the protracted review process due to delayed submission of referee reports. Your study has now been seen by one of the original referees, who now finds that their main concerns have been addressed. There remain only a few minor editorial points that have to be addressed before I can extend formal acceptance of the manuscript:

1. Please address the minor points from referee #1.

Please let me know if you have any further questions regarding any of these points. You can use the link below to upload the revised files.

Thank you again for giving us the chance to consider your manuscript for The EMBO Journal. I look forward to receiving the final version.

Referee #1:

This reviewer appreciates the authors' efforts in improving the manuscript, particularly by focusing on the key points, reanalyzing the protein data, and providing new data to support the claimed points. In general, conclusions drawn in the revised manuscript were backed up by more reliable data. What remains to be strengthened is the way the data is presented and described to push for the readability of these potentially novel discoveries. A few primary examples are given below.

Figure 4D is very challenging to understand. It's unclear where the insets at the bottom of 4D were relative to the top figures. What were the yellow arrowheads pointing at? The PCC=0.77 refers to just the co-localized spots shown, or is it calculated and averaged from multiple spots? Were the spots included in the analysis filtered by specific sizes? What was "spot signal" intended for? And the right panel for the "-PLA" should be blank (no PLA signal), not "merged" signals of DCP2-YFP and mScarlet-DCP1? The very brief description of the results in the text and the less-than-clear legend added the difficulties in understanding the key points here....

Figure 6 needs some work to improve the presentation and to separate the subfigures better for clarity. Figure 6B, the labels for regions 1-3, DCP1-GFP, and its two phosphovariants on the right were misplaced? What were the two asterisks for? Color for the "relative signal" bar is missing? The insets 1-3 were challenging to follow. Adding the magenta color or circling the regions on the figure above so the relative positions could be identified? Figure 6C needs some work to rearrange the display, resize some of the subfigures for clarity, and for the bar chart to be shown clearly, at least not with the labels of the genotypes all clustered/overlapped together.

Same as above, Figures 7B and 7C are challenging to separate, and the plots' organization is far from intuitive for readers...

Figure 8B, FM4-64 images were for WT, dcp1, and dcp2 mutants or WT, dcp1, DCP1, and dcp2 mutants (figure and legend were inconsistent).

More careful editing of the text might also help. For example, in line 93, ... "interacting with"?

RESPONSE TO REVIEWERS_EMBOJ-2022-111885R

Referee #1:
This reviewer appreciates the authors' efforts in improving the manuscript, particularly by focusing on the key points, reanalyzing the protein data, and providing new data to support the claimed points. In general, conclusions drawn in the revised manuscript were backed up by more reliable data. What remains to be strengthened is the way the data is presented and described to push for the readability of these potentially novel discoveries. A few primary examples are given below.
Thanks!
Figure 4D is very challenging to understand. It's unclear where the insets at the bottom of 4D were relative to the top figures. What were the yellow arrowheads pointing at? The PCC=0.77 refers to just the co-localized spots shown, or is it calculated and averaged from multiple spots? Were the spots included in the analysis filtered by specific sizes? What was "spot signal" intended for? And the right panel for the "-PLA" should be blank (no PLA signal), not "merged" signals of DCP2-YFP and mScarlet-DCP1? The very brief description of the results in the text and the less-than-clear legend added the difficulties in understanding the key points here....
To address this, we changed the image layout to make it clearer. The PCC refers to the colocalized DCP1-DCP2 shown (now described in the legend). The spot signal refers to DCP1-DCP2 interaction. To improve clarity, we have removed the arrowhead and labelled the insets as “1” and “2”. We made a new figure (Figure 4D is now Figure 5) and labelled the insets in the upper image with yellow rectangles.
Figure 6 needs some work to improve the presentation and to separate the subfigures better for clarity. Figure 6B, the labels for regions 1-3, DCP1-GFP, and its two phosphovariants on the right were misplaced? What were the two asterisks for? Color for the "relative signal" bar is missing? The insets 1-3 were challenging to follow. Adding the magenta color or circling the regions on the figure above so the relative positions could be identified? Figure 6C needs some work to rearrange the display, resize some of the subfigures for clarity, and for the bar chart to be shown clearly, at least not with the labels of the genotypes all clustered/overlapped together.
The mistake has been corrected (misplacement). To address this comment, we split the image layout to make it clear. Now Figure 6B is Figure 7B. We added labels for the region. The asterisks showed a significant change in the signal ratio of PM and cytosol among the WT and two phosphovariants. Figure6C corresponds to Figure 8B, and we changed the display and resized the bar chart to make it clearer.
Same as above, Figures 7B and 7C are challenging to separate, and the plots' organization is far from intuitive for readers...
To address this, we split the image to make it clearer. For clarity, Figures 7B and C correspond to Figure 9, panels B-D.
Figure 8B, FM4-64 images were for WT, dcp1, and dcp2 mutants or WT, dcp1, DCP1, and dcp2 mutants (figure and legend were inconsistent).
Thanks, this has been amended and the legend changed accordingly.
More careful editing of the text might also help. For example, in line 93, ... "interacting with"?
Thanks! We have edited the text, accordingly.

2nd Revision - Editorial Decision **18th Jan 2023**

Thank you for fixing the final minor issues. I am now pleased to inform you that your manuscript has been accepted for publication in the EMBO Journal.

EMBO Press Author Checklist

Corresponding Author Name: Panagiotis Moschou	
Journal Submitted to: The EMBO Journal	
Manuscript Number: EMBOJ-2022-111885	

USEFUL LINKS FOR COMPLETING THIS FORM
The EMBO Journal - Author Guidelines
EMBO Reports - Author Guidelines
Molecular Systems Biology - Author Guidelines
EMBO Molecular Medicine - Author Guidelines

Reporting Checklist for Life Science Articles (updated January 2022)
This checklist is adapted from Materials Design Analysis Reporting (MDAR) Checklist for Authors. MDAR establishes a minimum set of requirements in transparent reporting in the life sciences (see Statement of Task: 10.31222/osf.io/9sm4x). Please follow the journal's guidelines in preparing your manuscript.
Please note that a copy of this checklist will be published alongside your article.

Abridged guidelines for figures
1. Data
The data shown in figures should satisfy the following conditions:
- the data were obtained and processed according to the field's best practice and are presented to reflect the results of the experiments in an accurate and unbiased manner.
- ideally, figure panels should include only measurements that are directly comparable to each other and obtained with the same assay.
- plots include clearly labeled error bars for independent experiments and sample sizes. Unless justified, error bars should not be shown for technical replicates.
- if n<5, the individual data points from each experiment should be plotted. Any statistical test employed should be justified.
- Source Data should be included to report the data underlying figures according to the guidelines set out in the authorship guidelines on Data Presentation.

2. Captions
Each figure caption should contain the following information, for each panel where they are relevant:
- a specification of the experimental system investigated (eg cell line, species name).
- the assay(s) and method(s) used to carry out the reported observations and measurements.
- an explicit mention of the biological and chemical entity(ies) that are being measured.
- an explicit mention of the biological and chemical entity(ies) that are altered/varied/perturbed in a controlled manner.
- the exact sample size (n) for each experimental group/condition, given as a number, not a range;
- a description of the sample collection allowing the reader to understand whether the samples represent technical or biological replicates (including how many animals, litters, cultures, etc.).
- a statement of how many times the experiment shown was independently replicated in the laboratory.
- definitions of statistical methods and measures:
  - common tests, such as t-test (please specify whether paired vs. unpaired), simple χ2 tests, Wilcoxon and Mann-Whitney tests, can be unambiguously identified by name only, but more complex techniques should be described in the methods section;
  - are tests one-sided or two-sided?
  - are there adjustments for multiple comparisons?
  - exact statistical test results, e.g., P values = x but not P values < x;
  - definition of 'center values' as median or average;
  - definition of error bars as s.d. or s.e.m.

Please complete ALL of the questions below.
Select "Not Applicable" only when the requested information is not relevant for your study.

Materials

Newly Created Materials	Information included in the manuscript?	In which section is the information available? (Reagents and Tools Table, Materials and Methods, Figures, Data Availability Section)
New materials and reagents need to be available; do any restrictions apply?	Not Applicable	

Antibodies	Information included in the manuscript?	In which section is the information available? (Reagents and Tools Table, Materials and Methods, Figures, Data Availability Section)
For **antibodies** provide the following information:<br>- Commercial antibodies: RRID (if possible) or supplier name, catalogue number and or/clone number<br>- Non-commercial: RRID or citation	Yes	Materials and methods

DNA and RNA sequences	Information included in the manuscript?	In which section is the information available? (Reagents and Tools Table, Materials and Methods, Figures, Data Availability Section)
**Short novel DNA or RNA including primers, probes**: provide the sequences.	Yes	Appendix Tables 1-3

Cell materials	Information included in the manuscript?	In which section is the information available? (Reagents and Tools Table, Materials and Methods, Figures, Data Availability Section)
**Cell lines:** Provide species information, strain. Provide accession number in repository **OR** supplier name, catalog number, clone number, and/**OR** RRID.	Not Applicable	
**Primary cultures:** Provide species, strain, sex of origin, genetic modification status.	Not Applicable	
Report if the cell lines were recently **authenticated** (e.g., by STR profiling) and tested for mycoplasma contamination.	Not Applicable	

Experimental animals	Information included in the manuscript?	In which section is the information available? (Reagents and Tools Table, Materials and Methods, Figures, Data Availability Section)
**Laboratory animals or Model organisms:** Provide species, strain, sex, age, genetic modification status. Provide accession number in repository **OR** supplier name, catalog number, clone number, **OR** RRID.	Not Applicable	
**Animal observed in or captured from the field:** Provide species, sex, and age where possible.	Not Applicable	
Please detail **housing and husbandry conditions**.	Not Applicable	

Plants and microbes	Information included in the manuscript?	In which section is the information available? (Reagents and Tools Table, Materials and Methods, Figures, Data Availability Section)
**Plants:** provide species and strain, ecotype and cultivar where relevant, unique accession number if available, and source (including location for collected wild specimens).	Yes	Materials and Methods
**Microbes:** provide species and strain, unique accession number if available, and source.	Yes	Materials and Methods

Human research participants	Information included in the manuscript?	In which section is the information available? (Reagents and Tools Table, Materials and Methods, Figures, Data Availability Section)
If collected and within the bounds of privacy constraints report on age, sex and gender or ethnicity for all study participants.	Not Applicable	

Core facilities	Information included in the manuscript?	In which section is the information available? (Reagents and Tools Table, Materials and Methods, Figures, Data Availability Section)
If your work benefited from core facilities, was their service mentioned in the acknowledgments section?	Not Applicable	

Design

Study protocol	Information included in the manuscript?	In which section is the information available? (Reagents and Tools Table, Materials and Methods, Figures, Data Availability Section)
If study protocol has been **pre-registered, provide DOI in the manuscript**. For clinical trials, provide the trial registration number **OR** cite DOI.	Not Applicable	
Report the **clinical trial registration number** (at ClinicalTrials.gov or equivalent), where applicable.	Not Applicable	

Laboratory protocol	Information included in the manuscript?	In which section is the information available? (Reagents and Tools Table, Materials and Methods, Figures, Data Availability Section)
Provide DOI OR other citation details if **external detailed step-by-step protocols** are available.	Yes	Main text, Arora et al., 2021

Experimental study design and statistics	Information included in the manuscript?	In which section is the information available? (Reagents and Tools Table, Materials and Methods, Figures, Data Availability Section)
Include a statement about **sample size** estimate even if no statistical methods were used.	Yes	Materials and Methods, Figure captions, Source Data ZIP folders structured
Were any steps taken to minimize the effects of subjective bias when allocating animals/samples to treatment (e.g. **randomization procedure**)? If yes, have they been described?	Yes	Materials and methods, statistics, dcp1-3 only one case for the SCAR2 localization
Include a statement about **blinding** even if no blinding was done.	Yes	Materials and methods, statistics, dcp1-3 only one case for the SCAR2 localization
Describe **inclusion/exclusion criteria** if samples or animals were excluded from the analysis. Were the criteria pre-established?<br><br>If sample or data points were omitted from analysis, report if this was due to attrition or intentional exclusion and provide justification.	Yes	No data have been removed or excluded. The raw data can be found in this article (Source Data)
For every figure, are **statistical tests** justified as appropriate? Do the data meet the assumptions of the tests (e.g., normal distribution)? Describe any methods used to assess it. Is there an estimate of variation within each group of data? Is the variance similar between the groups that are being statistically compared?	Yes	Materials and methods, Figure captions, Source Data ZIP folders structured

Sample definition and in-laboratory replication	Information included in the manuscript?	In which section is the information available? (Reagents and Tools Table, Materials and Methods, Figures, Data Availability Section)
In the figure legends: state number of times the experiment was **replicated** in laboratory.	Yes	Captions, designated a N
In the figure legends: define whether data describe **technical or biological replicates**.	Yes	Technical replicates and range denoted as n, in captions

Ethics

Ethics	Information included in the manuscript?	In which section is the information available? (Reagents and Tools Table, Materials and Methods, Figures, Data Availability Section)
Studies involving **human participants**: State details of **authority granting ethics approval** (IRB or equivalent committee(s), provide reference number for approval.	Not Applicable	
Studies involving **human participants**: Include a statement confirming that **informed consent** was obtained from all subjects and that the experiments conformed to the principles set out in the WMA Declaration of Helsinki and the Department of Health and Human Services Belmont Report.	Not Applicable	
Studies involving **human participants:** For publication of **patient photos**, include a statement confirming that consent to publish was obtained.	Not Applicable	
Studies involving experimental **animals**: State details of **authority granting ethics approva**l (IRB or equivalent committee(s), provide reference number for approval. Include a statement of compliance with ethical regulations.	Not Applicable	
Studies involving **specimen and field samples:** State if relevant **permits** obtained, provide details of authority approving study; if none were required, explain why.	Not Applicable	

Dual Use Research of Concern (DURC)	Information included in the manuscript?	In which section is the information available? (Reagents and Tools Table, Materials and Methods, Figures, Data Availability Section)
Could your study fall under dual use research restrictions? Please check biosecurity documents and list of **select agents and toxins** (CDC): https://www.selectagents.gov/sat/list.htm	Not Applicable	
If you used a select agent, is the security level of the lab appropriate and reported in the manuscript?	Not Applicable	
If a study is subject to dual use research of concern regulations, is the name of the **authority granting approval and reference number** for the regulatory approval provided in the manuscript?	Not Applicable	

Reporting

The MDAR framework recommends adoption of discipline-specific guidelines, established and endorsed through community initiatives. Journals have their own policy about requiring specific guidelines and recommendations to complement MDAR.

Adherence to community standards	Information included in the manuscript?	In which section is the information available? (Reagents and Tools Table, Materials and Methods, Figures, Data Availability Section)
State if relevant guidelines or checklists (e.g., **ICMJE, MIBBI, ARRIVE, PRISMA**) have been followed or provided.	Not Applicable	
For **tumor marker prognostic studies,** we recommend that you follow the **REMARK** reporting guidelines (see link list at top right). See author guidelines, under 'Reporting Guidelines'. Please confirm you have followed these guidelines.	Not Applicable	
For **phase II and III randomized controlled trials**, please refer to the **CONSORT** flow diagram (see link list at top right) and submit the CONSORT checklist (see link list at top right) with your submission. See author guidelines, under 'Reporting Guidelines'. Please confirm you have submitted this list.	Not Applicable	

Data Availability

Data availability	Information included in the manuscript?	In which section is the information available? (Reagents and Tools Table, Materials and Methods, Figures, Data Availability Section)
Have **primary datasets** been deposited according to the journal's guidelines (see 'Data Deposition' section) and the respective accession numbers provided in the Data Availability Section?	Yes	Data Availability Section: The data generated or analyzed during this study are included in this published article. Additional information and materials generated for and/or reported in this article are available from the corresponding author upon request. The mass spectrometry proteomics data
Were **human clinical and genomic datasets** deposited in a public access-controlled repository in accordance to ethical obligations to the patients and to the applicable consent agreement?	Not Applicable	
Are **computational models** that are central and integral to a study available without restrictions in a machine-readable form? Were the relevant accession numbers or links provided?	Not Applicable	
If publicly available data were reused, provide the respective **data citations in the reference list**.	Yes	Main text, designated as dataset ref
